# Drivers of human papillomavirus vaccine uptake in migrant populations and interventions to improve coverage: a systematic review and meta-analysis

**DOI:** 10.1016/S2468-2667(25)00148-3

**Published:** 2025-08-01

**Authors:** Michiyo Iwami, Oumnia Bouaddi, Mohammad S Razai, Rania Mansour, Beatriz Morais, Nafeesa Mat Ali, Alison F Crawshaw, Sainabou Bojang, Farah Seedat, Anna Deal, Sophie Webb, Jessica Carter, Nathaniel Aspray, Nuria Sanchez Clemente, Juan Arroyo-Laguna, Sanjeev Krishna, Yolanda Augustin, Henry M Staines, Sally Hargreaves

**Affiliations:** The Migrant Health Research Group, Institute for Infection and Immunity, School of Health and Medical Sciences, City St George’s, University of London, London, UK; Mohammed VI International School of Public Health, Mohammed VI University of Sciences and Health, Casablanca, Morocco; Department of Public Health and Clinical Research, Mohammed VI Center for Research and Innovation, Rabat, Morocco; Barcelona Institute for Global Health, Hospital Clinic – University of Barcelona, Barcelona, Spain; The Migrant Health Research Group, Institute for Infection and Immunity, School of Health and Medical Sciences, City St George’s, University of London, London, UK; Primary Care Unit, Department of Public Health and Primary Care, University of Cambridge, Cambridge, UK; The Migrant Health Research Group, Institute for Infection and Immunity, School of Health and Medical Sciences, City St George’s, University of London, London, UK; Department of General Surgery, Mayo Clinic, Phoenix, AZ, USA; Institute for Infection and Immunity, School of Health and Medical Sciences, City St George’s, University of London, London, UK; The Migrant Health Research Group, Institute for Infection and Immunity, School of Health and Medical Sciences, City St George’s, University of London, London, UK; Prevention, Inequalities and Commissioning, City and Hackney Public Health Team, Hackney Council, London, UK; The Migrant Health Research Group, Institute for Infection and Immunity, School of Health and Medical Sciences, City St George’s, University of London, London, UK; Institute for Infection and Immunity, School of Health and Medical Sciences, City St George’s, University of London, London, UK; The Migrant Health Research Group, Institute for Infection and Immunity, School of Health and Medical Sciences, City St George’s, University of London, London, UK; Institute of Social Analytics and Strategic Intelligence Pulso PUCP, Faculty of Social Sciences, Pontifical Catholic University of Peru, Lima, Peru; Institute for Infection and Immunity, School of Health and Medical Sciences, City St George’s, University of London, London, UK; Institut Für Tropenmedizin, Eberhard Karls Universität Tübingen and German Center for Infection Research, Tübingen, Germany; Institute for Infection and Immunity, School of Health and Medical Sciences, City St George’s, University of London, London, UK; The Migrant Health Research Group, Institute for Infection and Immunity, School of Health and Medical Sciences, City St George’s, University of London, London, UK

## Abstract

**Background:**

WHO’s Cervical Cancer Elimination Initiative has set a target for 90% of girls to be fully vaccinated against human papillomavirus (HPV) by the age of 15 years by 2030, to substantially reduce deaths from cervical and other HPV-related cancers. However, progress has been slow, with only 27% global vaccine coverage in 2023. Migrants are an under-immunised group globally for many vaccine-preventable diseases, with data showing that they experience a high burden of HPV infection and widespread HPV under-immunisation. We aimed to identify drivers of HPV vaccine uptake in migrants, as well as assess uptake and explore recommended approaches, strategies, and best practices to promote uptake in migrant communities.

**Methods:**

In this systematic review and meta-analysis, we searched seven databases and several grey literature sources for information published in any language between Jan 1, 2006, and Dec 4, 2024, on the drivers of HPV vaccine uptake among migrants globally. Defining migrants as foreign-born nationals, we included qualitative and quantitative cross-sectional studies, cohort studies, and randomised controlled trials focused on first-generation and second-generation migrants and excluded studies of internal migrants. Outcomes were frequency and percentage of HPV vaccine uptake; factors positively or negatively influencing uptake; and recommended approaches, strategies, and best practices to promote uptake as reported by study authors or participants. We conducted a hybrid thematic analysis using the WHO Behavioural and Social Drivers of Vaccination model to map drivers of uptake, and a random-effects meta-analysis to calculate pooled estimates of uptake. Risk of bias was assessed using Joanna Briggs Institute checklists. This study is registered with PROSPERO, CRD42022347513.

**Findings:**

Of 3562 records returned by the search, 117 studies were included in the analysis, involving 5 638 838 participants across 16 countries and one territory, of whom 933 189 were first-generation and second-generation migrants. The pooled estimates of HPV vaccine uptake were 23·0% (95% CI 10·0–44·0; *I*^2^=99·3%; n=7614) among female migrants, 21·0% (5·0–58·0; *I*^2^=99·3%; n=2764) among male migrants, and 17·0% (8·0–33·0; *I*^2^=98·0%; n=3583) among male and female migrants combined. 79 (68%) studies were considered at low risk of bias, 32 (27%) were considered at moderate risk, and six (5%) were considered at high risk. Factors negatively influencing vaccine uptake included concerns about vaccine safety, cultural beliefs, uncertainty and low levels of knowledge about HPV vaccines or infection, exposure to negative information, and lack of recommendations from health-care providers. Practical barriers to uptake included little information on services, language barriers, logistical challenges, and the high cost of the vaccine. Enablers mainly included positive perceptions and trust in the vaccine and health-care providers, realistic expectations from parents regarding adolescents’ sexual activity, a sense of responsibility, recommendations from health-care providers, and support from social networks. Recommended strategies and interventions to improve uptake included culturally sensitive messaging and tailored communication for different target groups (eg, parents or caregivers and adolescents). Deploying trusted mediators (eg, peer school health promoters, religious champions, and community health workers) was key, alongside implementing practical solutions to address missed opportunities (eg, bundling HPV vaccination with other services), implementing eHealth initiatives, ensuring strong provider recommendations, reducing access barriers (eg, through walk-in, mobile, and outreach services), and strengthening vaccination monitoring systems.

**Interpretation:**

We show that migrants globally face complex individual, family and social, and provider-level and system-level barriers to HPV vaccination, resulting in low uptake of HPV vaccines and missed opportunities for protection. In many low-income and middle-income countries, there is little to no availability of vaccines and/or the recipient must pay for them. Achieving global commitments to universal and equitable immunisation across the life course—and making progress towards cervical cancer elimination—requires these barriers to be addressed through multipronged strategies. Collaborative efforts with migrant communities are essential to co-develop effective, tailored delivery models that meet their unique needs.

## Introduction

Human papillomavirus (HPV) causes multiple cancers (eg, cervical, oropharyngeal, vaginal, penile, and anal) and genital warts.^1^ HPV is responsible for more than 95% of cervical cancer cases globally,^2^ and is preventable through screening and vaccination.^3^ 662 301 new cases of cervical cancer were reported worldwide in 2022, with 94% of associated deaths in low-income and middle-income countries (LMICs).^2,4^ HPV vaccination has been a primary prevention strategy since 2006. Various HPV vaccines (eg, bivalent, quadrivalent, and nonavalent vaccines) have been developed to prevent different HPV-associated cancers and are reported to be safe and highly effective.^5^

WHO prioritises girls aged 9–14 years to receive an HPV vaccine before becoming sexually active,^2^ and set a global target for 90% of girls to receive a vaccine by the age of 15 years before 2030.^6^ Progress towards this target has been slow. In 2023, global coverage of the HPV vaccination programme in girls (defined as receiving at least one dose) was estimated at 27%—ranging from 68% in the WHO region of the Americas to 1% in the Eastern Mediterranean region^7^—with LMICs lagging behind considerably. Less than 25% of low-income countries had introduced HPV vaccination into their Essential Programmes on Immunization by 2022.^8^ A new low-dose vaccine is anticipated to accommodate vaccination for other populations, including boys, adults (eg, aged 27–45 years), and girls aged 9–14 years in low-income countries who are not in school, and is a means by which to increase vaccine supply among LMICs.

Migrants, defined by the International Organization for Migration as individuals who move away from their usual place of residence between or within a country,^9^ are disproportionately vulnerable to HPV infection and associated cancers. In Europe, studies in southern and central Italy reported significantly higher HPV infection rates and incidence of invasive cervical cancer among migrants than among native Italians, making migrants a priority group for HPV vaccine interventions.^10,11^

Despite global calls for equitable and universal access to life-course immunisation,^12^ migrants (including refugees and asylum seekers) are under-immunised for vaccine-preventable diseases.^13^ This under-immunisation is due to missed vaccines, doses, and boosters; unavailability of some vaccines in their countries of origin;^14,15^ and documented barriers to routine and catch-up vaccination.^16,17^ Existing literature shows disparities in access to and coverage of HPV vaccination between migrants and their host communities globally.^18^ A 2024 global systematic review including 31 442 migrants and refugees reported low HPV vaccination initiation rates (31·6% [95% CI 22·3–40·9]), with disparities by sex, region, and migration status.^19^ Similarly, a 2019 systematic review in the USA reported low initiation (~30%) and completion (14%) of HPV vaccination among children of migrant parents,^20^ and foreign-born individuals were 38% less likely to receive HPV vaccination than those born in the USA (odds ratio [OR] 0·62 [95% CI 0·56–0·69], *I*^2^=0%).^21^ Similar findings were observed in the UK, where a significant difference in the completion of HPV vaccination was reported between those who were born in the UK (87·2–89·8%) and migrants who were born in Poland (69·7–77·2%; p<0·01).^22^

Multiple studies have investigated barriers to uptake; however, no comprehensive effort has been made to synthesise this information to identify drivers of HPV vaccine uptake in migrants worldwide. Such a synthesis is key to identifying intervention targets aimed at increasing vaccine uptake and to achieving cervical cancer elimination and global HPV vaccination goals.^6^ We aimed to identify drivers of HPV vaccine uptake in migrants—using WHO’s Behavioural and Social Drivers of Vaccination (BeSD) framework^23^—and to explore HPV vaccine uptake as well as approaches, strategies, and best practices to promote uptake.

## Methods

### Search strategy and selection criteria

For this systematic review and meta-analysis, eligibility criteria were developed using the population, intervention, comparison, outcome, and study design (PICOS) framework (appendix p 7). We included studies published in any language from Jan 1, 2006 (the year in which the first HPV vaccine became available^24^), to Dec 4, 2024. We focused on migrants who were defined as foreign-born nationals^9^ (ie, we excluded internal migrants) and included both first-generation and second-generation migrants. These studies reported on factors influencing HPV vaccine uptake among adolescent and adult migrants; children of migrant parents eligible for HPV vaccination programmes irrespective of gender, sex, or age; and other stakeholders, including health-care providers. We included qualitative and quantitative cross-sectional studies, cohort studies, and randomised controlled trials. Studies were excluded if they did not disaggregate data for migrants, meet our definition of a migrant, or report on factors influencing HPV vaccination.

We searched seven databases (MEDLINE, Embase, American Psychological Association PsycINFO, Global Health, Cumulative Index to Nursing and Allied Health Literature, Scopus, and the Cochrane Library [Cochrane Database of Systematic Reviews and Cochrane Central Register of Controlled Trials]) for global literature published between Jan 1, 2006, and Dec 4, 2024, without language restrictions. The search combined free-text and subject heading terms for migrant, vaccination, and HPV separated by Boolean operators (see appendix pp 8–9 for full search strategy). We conducted an extensive search of the grey literature through websites of relevant international organisations (ie, WHO; Gavi, the Vaccine Alliance; International Organization for Migration; United Nations High Commissioner for Refugees; ReliefWeb; and Refworld) and Google Scholar. We searched the reference lists of the identified relevant systematic reviews by hand. All records were uploaded onto Covidence. Duplicate records were removed, and three authors (MI, MSR, and RM) conducted title and abstract screening and full-text review. Disagreements were resolved through discussion and corroborated by a senior author (SH).

This review was guided by the PRISMA guidelines 2020,^25^ and the protocol has been registered in PROSPERO (CRD42022347513).^26^

### Data analysis

Two authors (MI and MSR) extracted the data using a predefined form that was piloted and refined. We extracted information on study characteristics (eg, study design, country of study, year of study, and setting), participant characteristics (ie, participant numbers, participant groups, gender and/or sex, age, nativity, country or region of origin, race and/or ethnicity, and migrant status), aims, methods (ie, data collection and analysis and participant recruitment), interventions or HPV vaccination programmes, outcomes (ie, HPV vaccine uptake and factors influencing uptake), and recommendations (by authors or participants). Discrepancies were resolved by consensus with input from a senior author (SH).

Outcomes were frequency and percentage of HPV vaccine uptake (disaggregated by sex where possible); factors positively or negatively influencing uptake; and recommended approaches, strategies, and best practices to promote uptake as reported by study authors or participants. Comparison groups (eg, host communities) were included where possible.

Three authors (MI, RM, and OB) did the risk of bias assessment using Joanna Briggs Institute (JBI) critical appraisal tools. Two of these three authors conducted this assessment independently for each study. Each study type was assessed using the corresponding JBI checklist. Mixed-methods studies were appraised using combinations of both qualitative and cross-sectional quantitative JBI checklists. Items within checklists were rated yes (score 1), no or not sure (score 0), or not applicable (excluded from the total item count). The risk of bias in each study was presented as the mean percentage of yes scores. Studies scoring below 60% were considered high-risk, those scoring 60–80% were considered moderate-risk, and those scoring 80% and over were considered low-risk. No studies were excluded from the systematic review and meta-analysis on the basis of risk of bias assessments, but these assessments were considered in the sensitivity analysis.

Given the anticipated high levels of heterogeneity, we conducted random-effects meta-analysis to assess HPV vaccine uptake among migrants globally, focusing on studies conducted since 2014. We pooled studies with similar definitions of the receipt of HPV vaccination that were aligned with more current HPV vaccination policies (eg, where initiation was defined as the percentage of migrants who received at least one dose of the vaccine or as the receipt of HPV vaccination but excluding completion), and where there were at least three studies. We included only first-generation migrants, and did separate meta-analyses for uptake in female migrants, male migrants, and male and female migrants combined. We used Metaprop function in R software (version 4.3.0) to calculate the pooled percentage of uptake and corresponding 95% CIs. We quantified heterogeneity between study results using the *I*^2^ statistic. To explore the influence of study quality on our findings, we conducted sensitivity analyses. Results were presented in forest plots.

For drivers of uptake, we conducted hybrid thematic analysis^27,28^ using the WHO BeSD model as an a priori framework to systematically organise and structure the data synthesis.^23^ This framework contains four domains that influence the uptake of recommended vaccines: what people think and feel, social processes, motivation, and practical issues (appendix p 3). MI generated the preliminary codes deductively, which were grouped as subthemes mapped onto relevant priori constructs of the BeSD framework. Codes emerged inductively were added and new subthemes and domains were generated accordingly. Emergent coding structure was developed and iteratively refined through constant comparison. Influencing factors from quantitative data were coded considering the name of variables, followed by further categorising them into significant (p<0·05) or not significant (p≥0·05) negatively or positively influencing factors. Results were refined and validated by one author (OB) and a senior author (SH). Frequency counts of codes were considered in the interpretation of findings and discussion.

### Role of the funding source

The funders of the study had no role in study design, data collection, data analysis, data interpretation, or writing of the report.

## Results

**Figure 1:**
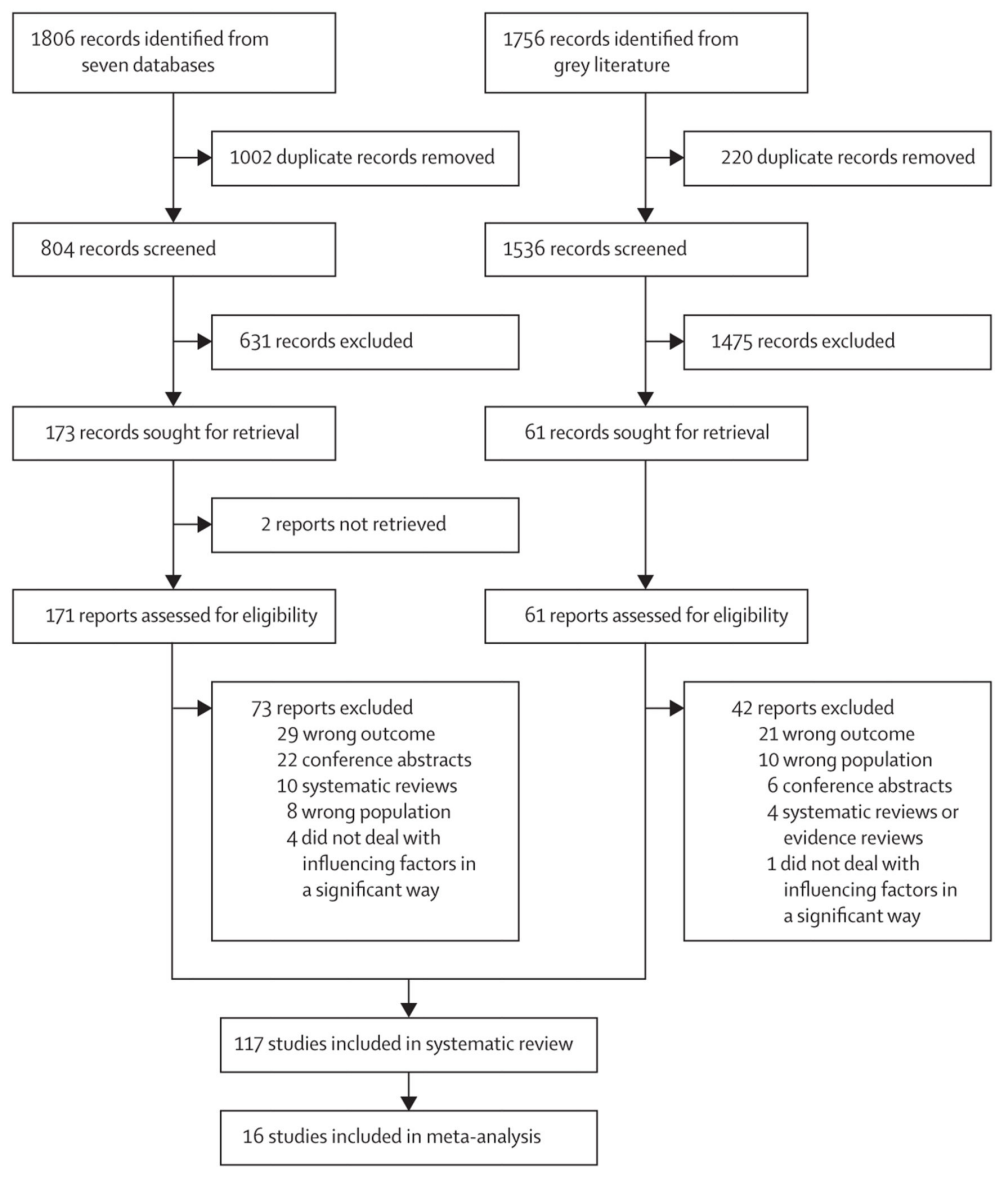
Study selection

Our search returned 3562 records (1806 database records and 1756 records from websites), of which 2340 underwent title and abstract screening. After full-text assessment, we included 117 studies in the systematic review, involving 5 638 838 participants across 16 countries and one territory (including 933 189 first-generation and second-generation migrants; figure 1; table).^29–145^ 16 unique studies were included in the meta-analysis.^48,80,88,93,94,104,113,115,119,124,127,131,134,137,143,145^ Most studies were from high-income countries, with 77 (66%) conducted in the USA or US territory^29,30,42,49,52–56,58–60,62,63,65–69,71,73,74,76,79,81–84, 89–91,93–114,116,117,120–123,125–128,130,132–141,143–145^ and most of the remainder (28; 24%) conducted in Europe^31–33,35–41,43–45,48,50,51,64,70,72,75,78, 85–88,115,118,119^—particularly in Scandinavian countries (16 [57%] of 28 studies).^32,33,35–41,43–45,48,51,88,118^ Only one study was conducted in an LMIC (Nepal).^92^ For a map of study locations, see the appendix (p 4). Only one study was conducted in the Eastern Mediterranean region,^131^ and none were conducted in Latin America or Africa. A few studies reported specific migrant status, such as refugee (n=13),^37,46,47,58,61,66,80,92,96,100,115,124,138^ student (n=7),^55,60,131,137,140,143,145^ or migrant farm worker (n=3);^63,71,81^ however, the majority of studies provided either no information or unclear information on migrant status. The most frequent country of origin of migrants was Mexico (n=17),^42,56,59,63,76,79, 81–84,100,104,105,108,116,120,125^ followed by China (n=12)^34,36,55,85,99,110,117,124,130, 140,142,145^ and Somalia (n=9).^30,36,37,48,52,53,73,111,138^ The most frequent region of origin was the region of the Americas, followed by the European region; the Eastern Mediterranean region was the least frequent region of origin. The most represented ethnic groups of migrants were Hispanic^30,58,63,67,79,93–96,98,100,104,108,121,127,128,133,137^ or Latina, Latino, or Latinx (n=32),^49,54,58,68,71,91,96,98–110,114,116,122,130,132,137^ White and non-Hispanic or Latino White (n=27),^30,42,63,68,79,85, 93–95,99,104,107–110,114,121,122,127,128,130,133,134,137,138,143,145^ Asian (n=22),^30,46,68,95, 99,104,107–110,114,117,122,127,130,133,134,137,138,143–145^ and Black and non-Hispanic Black (n=21).^30,52,73,79,93–95,98,104,105,108,114,121,122,124,127,134,137, 138,143,145^ For more details about the country or region of origin and the race and/or ethnicity of migrants, see the appendix (pp 10–13). The studies included various participant groups, including vaccine recipients only (n=34),^34,36,37,43,44,47,54,55,60,61,73,76,79,93,94,97,105–107,110,113,114,117,119,127,128,131, 134,136–138,140,143,145^ parents or caregivers only (n=21),^31,52,58,59,63,64,70,81, 83,89–91,95,96,100,101,120,123,124,133,144^ mothers only (n=17),^29,42,53,66,69,74,77,82,84,92,102,116,126,130,132,135,139^ fathers only (n=1),^62^ and health-care providers only (n=3).^57,67,68^ The majority of studies (n=79) were considered to be at low risk of bias,^31,33,35–38, 40–44,46–48,51–77,82,85–89,91–95,97,103–112,114,117–119,121–123,126–129,132,133,135,137,144^ 32 studies were considered at moderate risk of bias,^30,32,34,39,45,49,50, 78–81,84,90,96,98–100,113,115,116,120,124,125,130,131,134,136,138–141,143^ and only six studies were considered at high risk of bias^29,83,101,102,142,145^ (appendix pp 14–97).

**Figure 2:**
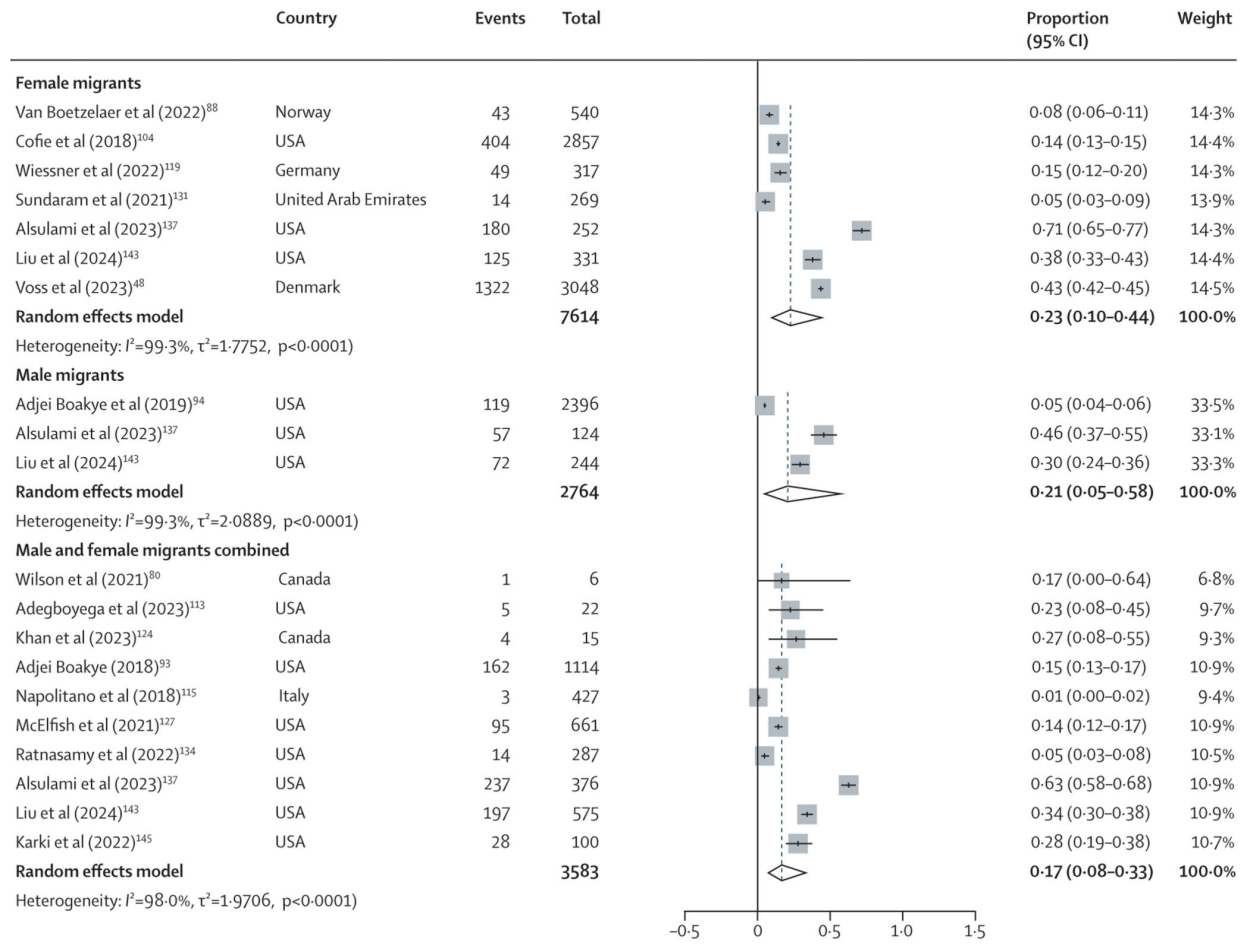
Forest plots showing pooled estimates of HPV vaccine uptake in migrants HPV=human papillomavirus.

Among 7614 female migrants, pooled uptake of the HPV vaccine was 23·0% (95% CI 10·0–44·4; *I*^2^=99·3%) across seven studies.^48,88,104,119,131,137,143^ Uptake among 2764 male migrants was lower, at 21·0% (5·0–58·0; *I*^2^=99·3%) across three studies^94,137,143^ (figure 2). The pooled uptake estimate for 3583 male and female migrants combined was 17·0% (8·0–33·0; *I*^2^=98·0%) across ten studies.^80,93,113,115,124,127,134,137,143,145^ We conducted sensitivity analyses including only studies assessed as having a low risk of bias for female migrants and for studies including male and female migrants combined. Pooled estimates remained largely unchanged in these analyses: 26·0% (10·0–53·0; *I*^2^=99·5%) for female migrants^48,88,104,119,137^ and 27·0% (7·0–62·0; *I*^2^=99·4%) for male and female migrants combined^93,127,137^ (appendix p 5).

**Figure 3:**
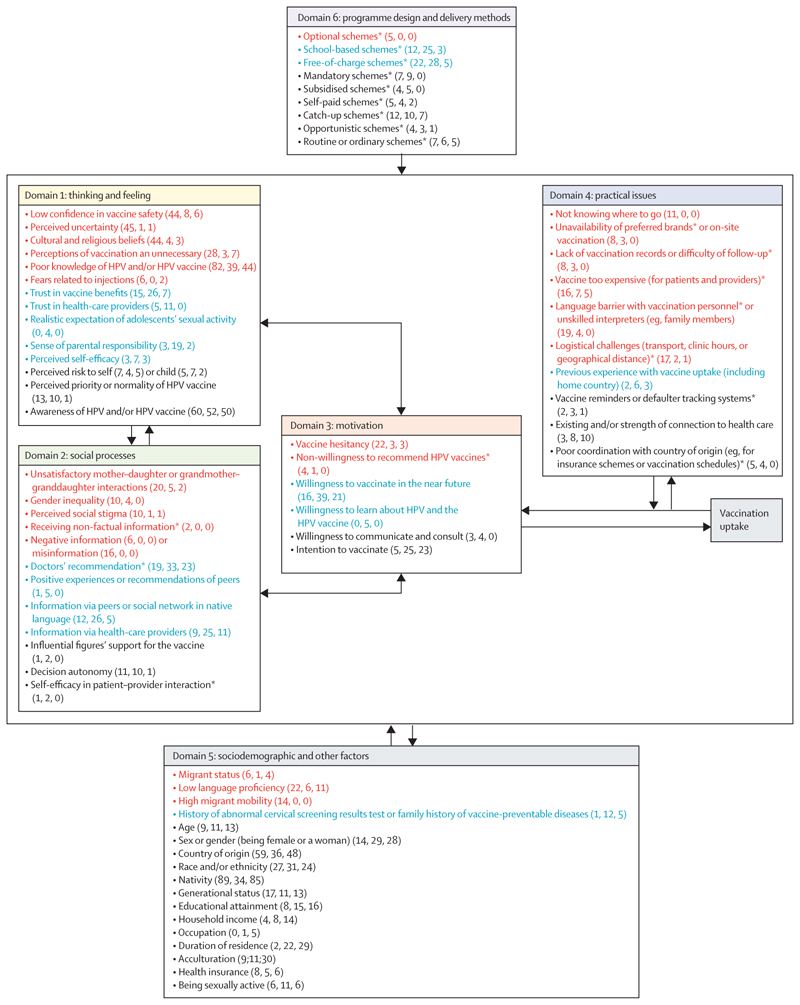
Behavioural and social drivers of HPV vaccination uptake in migrants Text in red, blue, and black represents factors that negatively influence uptake (or barriers), factors that positively influence uptake (facilitators), and factors that have an inconclusive or mixed effect on uptake, respectively. The numbers in parentheses indicate code frequency counts in which the factor negatively influenced uptake, positively influenced uptake, and had no significant or predominant effect on uptake, respectively. HPV=human papillomavirus. *Factors related to health-care providers or health-care systems.

The BeSD framework was adapted on the basis of the findings (figure 3, appendix pp 98–105). Domains 5 (sociodemographic and other factors) and 6 (programme design and delivery methods) emerged from the data and were added to the framework. The frequency counts of each factor under each domain are shown in figure 3 and the appendix (p 6). We present the findings separately for each domain. For cases in which the generational status is not mentioned, the data were from first-generation migrants (foreign-born). Migrant parents are referred to as first-generation migrants and their children as second-generation migrants.

Concerning domain 1, thoughts and feelings about HPV vaccination, the main factors negatively influencing uptake included concerns around vaccine safety,^29–31,42,51–54, 56,59,62,63,65,66,69–71,74–76,123,124,135,137,139,140,142,144,145^ cultural and religious beliefs (eg, a perceived risk of premarital sex or promiscuity or concerns about pork gelatine in vaccine manufacturing), ^29–31,50,52,56,57,61–65,68,69, 71,73,74,76,102,111,123,135,139^ uncertainty around HPV vaccines and infection (often linked to perceived needs for more information),^29–31,50,52–57,61, 63,65,66,69,75,76,91,102,111–113,115,123,124,142,144^ parents’ or caregivers’ thoughts that the vaccination was unnecessary because their daughters are too young or that the vaccine is only relevant within the context of marriage,^29–31,50,52,55,57,60–62,65,69,73,76,80,102,123,124,139^ and limited knowledge about HPV and/or the HPV vaccine.^29–31,51,52,54–57,59–62,64–66,69,71,73–76,78,80,81,95,96,102,108,111,113,115,117,123,124,129,131,135,137,140,142^

Factors positively influencing vaccine uptake included a feeling of parental responsibility for getting their eligible child vaccinated;^31,42,50,51,61,63,66,71,74,76,91,139^ parents’ more realistic understanding of children’s sexual activity,^59,66,68,91^ especially among Latinx parents;^59^ self-efficacy and confidence in one’s ability to engage in preventive action;^59,61,113,116,137^ confidence in the benefits of the vaccine;^29,30,53,54,56,61,62,64–66,71,74,75,80,91,113,135,137^ and trust in health-care providers.^29,30,57,62,68,69,71,77,124,135^ The influence of perceived risks of HPV infection and/or associated cancers to one’s child^31,42,49,56,57,66,69,71,116,132^ or oneself^31,49,55,61,70,73,112,116,132^ showed mixed results, and one’s partners were rarely investigated.

Regarding domain 2, social processes, the factors impeding HPV vaccine uptake included unsatisfactory mother–daughter or grandmother–granddaughter interactions (including spouse communication) and/or relationships (resulting in ineffective communication about child vaccination and sexual health),^29–31,50–53, 61,66,69,73,74,76,77,80,135,144^ and a gatekeeping role of grandmothers concerning health-seeking for their daughters or daughters-in-law.^75^ Poor relationships were reportedly more common in second-generation adolescent migrants, for whom mismatching communication exists with differential peer effects, leading to clashes of norms between mothers and daughters.^51^ Perceived social stigma was a key negative influencing factor, often rooted in cultural histories and negative past experiences (eg, unethical experimentation practices during the HIV epidemic among Haitians, and historical relations between Black Americans, who might not be foreign-born, and physicians).^29,113^ Social stigma was rooted in heightened embarrassment, because HPV vaccination was linked to promiscuity or sexually transmitted infections.^139,145^ One study found that greater knowledge was linked to reduced stigma around HPV infection and vaccination among international students.^55^

Gender inequality was reported as a key factor, whereby power dynamics within families—such as a strong paternal influence over maternal authority in health decision-making—complicated the vaccination process for daughters.^31^ Negative information^50,51,70,71,102^ and the spread of vaccine misconceptions or misinformation^30,31,61,71,75,76,102,135^ (via social media, short videos, or peers^76^) consistently deterred those seeking HPV vaccination.

Facilitators of HPV vaccine uptake included hearing positive experiences or advice from those who had themselves or their children vaccinated,^42,52,62,68,74^ receiving information about the vaccine through doctors’ recommendations,^29,50,52,54,56–58,61,62,65,66,68,69,71,74,75,77,80,102,131,132,144^ and receiving information about the vaccine via health-care providers or schools^29,31,51,53,58,59,63,64,69,74,78,81,89,92,112, 115,123,124,135,144^ or peers or social networks (in one’s native language).^42,50,51,53–55,58,59,62,65,67,70,71,74,75,77,92,115,135,144^ The effects of the information received depended on multiple factors, including the strength and framing of recommendations, communication methods, characteristics of sources and recipients,^52^ and personal preferences regarding information formats (ie, oral versus written and direct versus indirect communication with clinicians).^63^

Domain 3 considered the motivation for vaccination. Negatively influencing factors included a hesitancy to vaccinate among parents or caregivers,^29,32,73,75,80,102,116,132,135,140^ limited willingness of providers to recommend HPV vaccines (owing to low perceived priority, competing priorities, or preconception about migrants’ cultural beliefs),^71,102^ or the provision of non-factual information.^71^ Factors positively influencing uptake included willingness of the parent or caregiver to get themselves or their child vaccinated^29,59,61,62,66–68,70,72,75,78,80,101,102,113,115,132,133,135,144^ and to learn about HPV and HPV vaccines.^29,51,54,75,76^ Influences of the intention to vaccine were inconclusive.^65,69,76,100,111,116,132,139,140^ Among Asian immigrant college students residing in the USA, vaccine intention was a significant mediator between HPV vaccine literacy and HPV vaccination.^140^ However, of 44 Haitian mothers or guardians living in the USA, 33 (75%) intended to get their daughters vaccinated in response to doctors’ recommendations, but only 14 (31%) of the 44 daughters subsequently received the vaccine (p=0·22).^69^

Domain 4, considering practical issues, included more negatively influencing factors than other domains. Practical issues preventing uptake included migrants’ lack of knowledge about where vaccines are available,^57,71,75,98,111,115, 123,135,145^ unavailability of preferred brands,^70^ unavailability of on-site vaccination,^56,63,71,72,77,102^ absence of vaccination records,^52,67,71,75,77^ and difficulty or loss to follow-up.

Affordability was also a barrier, as vaccines were too expensive for beneficiaries and providers owing to the scarcity or absence of funding.^54,56,57,61,67,71,72,75,76,98,113,123,135,142,145^ Poor access was reported as a result of multiple logistical challenges, including difficulties with transport, time constraints, inadequate clinic hours, and geographical distance.^56,57,63,66,71,75,76,80,123,124^ Poor coordination between host and home countries (eg, regarding insurance or vaccine schedules) contributed to poor continuity of care.^63,70,71,75^

Language barriers between vaccination personnel and migrants posed challenges, with unskilled interpreters sometimes providing insufficient or inaccurate information (eg, young daughters acting as interpreters for their parents or caregivers).^56,66,68,70,71,73,75–78,102,124^ Vaccine reminders or defaulter tracking systems showed inconsistent effects on promoting uptake.^40,63,71^

Facilitators included previous vaccine uptake (including in the home country), which targeted younger migrants (aged 11–19 years).^32,40,51,62,63^ In one study, after receiving written reminders, vaccine uptake was dependent on the mothers’ region of origin: daughters of immigrant mothers of non-western ethnicity were twice as likely to receive the HPV vaccine than Danish natives (OR 2·02 [95% CI 1·57–2·59]; p=0·0000), but no difference was found for daughters of immigrant mothers of western ethnicity.^40^ Another facilitator was the strength of connection to health care, such as having a usual place to seek care or an increased number of health-care visits in the past year; however, although this factor was significant in non-migrants,^93,94^ it was not significant in migrants.^93,94,103,132,141^

Factors within domain 5, sociodemographic and other factors, extended beyond health or vaccination-specific issues yet probably influenced uptake. However, these factors were inconclusive. Overall, significant predictors of low uptake included migrant status,^56,71,75,137^ high migrant mobility,^38,56,63,64,66,67,70,71,76,77^ and low language proficiency of vaccine recipients and their parents.^56–58,60,66,70,71,73,75,78,97,102^ Predictors of high uptake included previous experience or family history with vaccine-preventable diseases (eg, cervical cancer) or abnormal cervical screening results.^42,62,67,68,71,91,135^ Gender and/or sex had moderate effects on initiation, completion, and uptake of the HPV vaccine,^93,107,122,127^ but substantial effects on awareness of HPV and/or the HPV vaccine,^112,115^ with women performing better than men.

By contrast, duration of residence,^31,36,37,46,50,58,67,79,81,83,88, 94,96,97,99,100,102,104,112,113,115,120,122,125,130,132–134,137^ acculturation,^51,53,55,58,66,69,82–84,89,90,112,121,125,137,142,144^ educational attainment,^44,46,51,68,71,80,81,89,93,94,102,103,105,112,115,116,118,129,131–133,137,139,144^ and household income^44,46,47,59,68,71,89,93,102,103,107,118,129,131–133,141,144^ showed inconsistent findings. Nativity, generational status, country or region of origin, and race and/or ethnicity also showed inconsistent results. These factors were intertwined with gender and sex, with women often showing higher initiation than men^93,105,107^ and immigrants showing lower uptake than descendants.^38^ However, the findings on gender and sex were not conclusive. For example, irrespective of nativity, women showed better completion rates than men of the full duration of a vaccination programme in Alberta, Canada; however, this difference was reduced to the point at which men even had a slightly better completion rate than women when excluding the first 2 years after programme implementation for each sex (full programme: 57·62% completion rate for immigrant women *vs* 44·72% for immigrant men; excluding the first 2 years of the programme for each sex: 63·96% [95% CI 63·24–64·67] *vs* 66·20% [65·17–67·23], respectively).^47^ Similarly, age exhibited inconsistent effects.

Domain 6 considered the HPV vaccination programme design and delivery methods. The studies included various delivery approaches with differential effects on HPV vaccination uptake. School-based programmes were among the most consistently effective approaches for improving uptake,^33–35,44,57,64,91,118^ with free school-based catch-up programmes showing more favourable outcomes in terms of equitable HPV vaccination initiation than free non-school-based catch-up approaches and free school-based ordinary programmes.^35^ The type of school also had an effect. In one study, public schools had the best performance in terms of HPV vaccination initiation, followed by private schools, whereas schools for those with special educational needs performed poorly.^48^ Another study reported that information on the HPV vaccine was not provided to an adolescent who attended a special educational needs school and her Arabic-speaking mother, resulting in no uptake.^77^

Overall, free vaccination programmes showed consistently positive effects on the initiation of HPV vaccination in migrants,^33–36,38,39,44,71,88,112,124^ compared with self-payment schemes. Initiation rates differed by migrant group, duration of residence, generational status, income, and type of vaccination programme (catch-up *vs* routine). In a study in Denmark, girls with a refugee background had significantly lower HPV vaccine uptake than native girls in both ordinary (OR 0·44 [95% CI 0·37–0·51]) and catch-up (0·61 [0·54–0·69]) programmes, but this difference remained significant only for the ordinary vaccination programme when adjusting for household income (0·73 [0·61–0·89] and 0·88 [0·76–1·01], respectively).^37^ Generational status affected initiation rates in routine and free-of-charge catch-up programmes, with lower rates among immigrants to Denmark (first-generation migrants) than among descendants (second-generation migrants) and native Danes.^36,38^ However, descendants had mixed experiences in the routine programme depending on their birth cohort, with those born in the more recent cohort (2001–03) having higher uptake [OR 1·15, 1·08–1·21] but those born in an earlier cohort (1996–2000) having lower uptake (0·65 [0·60–0·68]) than native Danes.^38^

Mandatory schemes had mixed effects.^42,73,91^ Support for these programmes varied, with some foreign-born parents showing stronger support for school mandates than those from other ethnic groups.^91^ Others expressed negative sentiments towards mandatory programmes, particularly when tied to legal residency requirements,^42^ or generating feelings of violation of their rights because of perceived reduced autonomy.^73^

Optional vaccination schemes had consistently negative effects on uptake, owing to a perceived lack of priority given to HPV vaccination by health-care providers and vaccine recipients.^57,71,75^

The studies recommended numerous strategies and reported various effective approaches to strengthen HPV vaccination among migrants (panel). Information on communication approaches and strategies was abundant, and focused on providing culturally and linguistically tailored messaging in suitable formats and venues, and through trusted messengers (eg, religious champions, community health workers, peer school health promoters, and health-care providers with a similar cultural background), to reach diverse groups effectively and provide clear information on the advantages and disadvantages of vaccination (eg, benefits of the vaccine and risks of HPV infection and associated cancers).^120,121^

Studies emphasised that behavioural change interventions should not be separated from efforts to improve the accessibility of vaccination.^116,140^ Priority was given to multilevel communication strategies targeting all stakeholders—including direct beneficiaries, parents or caregivers, and providers. Such strategies included comic books for adolescents, educational forums for mothers,^53,111^ and online continuous education courses for providers to enhance responsiveness and sensitivity to the needs of direct beneficiaries and parents.^30^

Inclusion of key messages in vaccine information sheets was considered essential, such as Halal certification^77^ and positive vaccination testimonials.^140^

Clear, accessible information concerning HPV and the HPV vaccine—covering the what, why, and how in lay terms—was important,^124^ including details on the prevalence of HPV infection in the host country, modes of transmission, exposure risks, and susceptibility.^113,144^

For young adult migrants, preferred formats included concise information, infographics, and statistics^113,145^ and narrative videos and audiovisual content in native languages broadcast on the television and/or radio.^60,65^ Effective channels were educational workshops,^63^ comic books,^53^ and targeted social media platforms such as college health centre Facebook pages.^145^ For parents and caregivers, recommended formats included radio programmes (for older parents, ie, >35 years),^100^ culturally relevant short stories,^31^ photonovelas or radionovelas presented by community health workers in communities with low literacy,^120^ and interactive educational forums.^111^ Flyers and pamphlets about the HPV vaccine were distributed at community venues such as clinics and churches.^63^ Hotlines staffed by doctors from the same country as the migrants, including Ukrainian doctors in Poland, were used.^75^ Because migrants often use multiple information sources, education focusing on accessing reliable health information and making the most of diverse resources was recommended.^135^

Male-targeted interventions were prioritised to challenge gender-related misconceptions about HPV vaccination. Recommended approaches included featuring male celebrities in health campaigns^31^ and implementing school-based programmes for boys to reduce stigma.^57^

The role of trusted mediators is crucial in delivering HPV vaccination messages. Recommended strategies included using peer health promoters in schools to educate students and serve as a liaison with health-care professionals,^106^ and engaging religious champions and community health workers to foster acceptance among caregivers.^31,77^ Emphasis was placed on involving community health workers and migrant representatives to deliver messages and co-design messages in line with cultural and religious values.

The role of health-care providers in promoting HPV vaccination was emphasised, with recommendations for active involvement in promotion and follow-up. Key strategies included framing vaccination in alignment with parents’ values, normalising the HPV vaccine, providing strong and clear recommendations, building trust, and encouraging proactive behaviour.^71^ Communication preferences varied: some migrant groups preferred in-person interactions,^52^ whereas others favoured oral and written communication in their native language at the clinic or sent to their homes.^63,71^

In framing communication, it was recommended to present HPV vaccination as part of general health promotion rather than solely as a measure to prevent sexually transmitted infections.^52,57^ Studies stressed that girls are at risk of HPV through their partners,^31^ a point that is often under-reported. The focus should be on risk perception, rather than stigmatising promiscuity.^31^

Although accessibility challenges were major barriers to vaccine uptake, recommendations in this area were sparse. Suggested solutions included walk-in centres,^71^ mobile clinics,^63^ school vaccine clinics,^124^ catch-up vaccine days,^124^ community pharmacies,^71,137^ transportation to clinics,^63^ and outreach services.^75,79,99,126^ Free or affordable vaccination^41,56,57,71,75,98,100,105,107,113,126,130,131,134,137^ and interpretation services at points of care^36,56,63,75,93,142^ were recommended.

Several studies highlighted the effectiveness of bundling approaches and making use of other health-care appointments to provide the vaccine. Bundling involved integrating HPV vaccination with other services or campaigns—including HIV programmes,^113^ sexual education,^55,113^ social and mass media campaigns,^113^ vaccination awareness events on campus,^131^ seasonal flu vaccination clinics on university campuses,^143^ and COVID-19 vaccination hubs.^140^ Making use of other routine health-care visits—such as general practitioner (primary care doctor), maternal health care, gynaecology, paediatric, college health centre, or childhood immunisation visits—was also recommended to administer the vaccine or raise awareness.^50,71,79,110,113,142,145^ This strategy ensures dedicated time for discussions between parents or caregivers and providers, increasing engagement and uptake.^63^

Establishing robust vaccine documentation system was essential. Recommendations included national, long-term monitoring systems to track vaccine uptake, including data on country of origin, migrant status, and race and/or ethnicity.^71,79,104^ Setting vaccination rate goals for educational institutions was considered important.^140^ For highly mobile groups (eg, farm workers), innovative strategies were suggested—including electronic vaccination booklets or records,^71^ eHealth tools for sharing health data with clinicians in host countries,^63^ and specialised health assessments for refugees on arrival.^138^ Reminders via text messages, telephone calls, or postcards were recommended to alert eligible populations and prompt follow-ups for incomplete vaccinations, with lists sent to general practitioners for tracking.^41^

## Discussion

This systematic review included 933 189 migrant participants from 16 countries and one territory, primarily in high-income countries. We identified various barriers to HPV vaccination uptake, including vaccine safety concerns, cultural beliefs, low knowledge of HPV, gender and family dynamics, negative information, lack of provider recommendations, language barriers, and high vaccine costs. Facilitators included trust in health-care providers, positive peer experiences, and free school-based catch-up delivery models. To improve uptake, recommended strategies focus on addressing missed opportunities through bundling approaches and the use of other health-care visits; using age-appropriate communication channels; and using culturally and linguistically tailored messaging that emphasises positive framing, aligns with parental values, and appeals to their sense of responsibility. Leveraging trusted mediators such as peers, community health workers, and religious leaders to deliver and co-design messages, alongside effective provider communication, were emphasised. Addressing physical barriers through outreach services and strengthening vaccination data systems, especially for mobile groups such as migrant farm workers, were key recommendations.

A global systematic review of studies published up to Dec 14, 2022, reported low pooled rates of HPV vaccination initiation among migrants and refugees: 31·6% (95% CI 22·3–40·9) for males and females combined, 17·4% (11·9–22·9) for females, and 3·0% (2·4–3·6) for males.^19^ Our meta-analysis, focusing on global studies conducted in the past decade only (ie, since 2014), suggests that vaccine uptake remains low in these populations: 17·0% (8·0–33·0; *I*^2^=98.0%) for males and females combined, 23·0% (10·0–44·0; *I*^2^=99·3%) for females, and 21·0% (5·0–58·0; *I*^2^=99·3%) for males.

In our review, recurring factors were individual-level knowledge, perceptions, beliefs, and social norms surrounding the vaccine among parents, caregivers, and recipients, and were reflected in multiple recommendations focusing on effective health information, education, and communication. These factors are consistent with previous literature reviews in non-migrant groups, which show low levels of knowledge about HPV and/or HPV vaccination among global indigenous communities,^146^ rural populations in the USA,^147^ non-immigrant parents of female adolescents in countries of the Association of Southeast Asian Nations,^148^ and minority ethnic adolescent girls.^149^ These findings highlight the importance of education in improving knowledge and shaping attitudes for migrants and vulnerable host populations alike. A previous systematic review of 2206 immigrant parents’ perceptions of HPV vaccination found low levels of awareness and negative perceptions, which often improved with information.^150^ In this review, we found that knowledge-related and perception-related barriers can be offset by receiving accurate and effective communication through trusted messengers and appropriate channels, including doctors from the same background recommending the vaccine or peers sharing positive experiences with vaccination services. We found hesitancy among health-care providers to recommend the vaccine if it conflicted with the cultural beliefs of migrants. Provider recommendations have an important role in HPV vaccine uptake;^147^ the absence of such recommendations negatively affects uptake, even if the vaccination is free of charge. A global meta-analysis on the effects of provider communication on HPV vaccination among 265 083 patients in the USA showed that provider recommendations substantially increased the initiation of HPV vaccination compared with no recommendation (60% *vs* 24%; pooled OR 10·1 [95% CI 7·6–13·4]; *I*^2^=99·4) and also increased vaccination completion.^151^ Discussions with health-care providers were associated with higher HPV vaccination initiation (pooled OR 12·4 [6·3–24·3]; *I*^2^=93·9).^151^ This result corroborates our findings, emphasising the need for active provider involvement in promoting vaccination.

Accessibility issues were major factors affecting uptake in this review. Although global coverage of HPV vaccination programmes remains low, high-income countries have achieved better coverage rates, although these rates fall short of WHO’s 90% target.^7^ First-dose programme coverage in 2023 was below target in the European region (62%), the region of the Americas (57%), and the Western Pacific region (70%).^7^ Vaccination rates among migrants and refugees are low, with a 2024 systematic review suggesting completion rates of 63·4% (95% CI 48·0–78·8) in the European region, 6·0% (3·9–8·2) in the region of the Americas, and 7·8% (7·09–8·52) in the Western Pacific region.^19^ Notably, 95% of populations vaccinated against HPV globally—including both migrants and non-migrants—are in high-income countries,^19^ suggesting that migrants in LMICs face greater challenges, which remain underexplored. There is an urgent need for affordable, locally manufactured HPV vaccines. A 2023 analysis of HPV programmes in 18 Asian LMICs identified major implementation challenges—including vaccine shortages, lack of subsidies, and reliance on out-of-pocket payments, contributing to low affordability.^152^ The authors reported a scarcity of national surveillance data on HPV vaccination.^152^ These health systems-related factors affect both local populations and migrants, with migrants facing additional barriers owing to lower health-care use and high mobility. Fortunately, WHO’s single-dose HPV vaccine guidelines^153^ present an opportunity to streamline vaccination delivery for migrants.

We found that school-based programmes were reported to achieve consistently superior results in promoting HPV vaccination initiation and uptake among migrants in high-income countries. School-based programmes are considered gold-standard models in high-income countries that achieved high vaccination coverage (eg, Sweden and Australia).^154^ These programmes have proven successful in LMICs, outperforming routine and facility-based immunisation approaches.^155^ However, their applicability to migrants in non-high-income countries, especially newly arrived or undocumented migrants, is limited. Ensuring that girls who do not attend school, a common group among migrants in LMICs, are reached is crucial. Some LMICs have implemented hybrid models combining school-based, health centre, and campaign-based delivery to reach these girls,^155^ and the potential of these models to reach migrant groups warrants further investigation.

Given our findings, a multipronged approach along BeSD pathways is desirable to address modifiable but complex barriers and take advantage of enablers for HPV vaccine uptake. Key strategies included culturally sensitive messaging and tailored communication for different target groups (eg, parents and caregivers or adolescents), framing information in a health-promotion context to address misconceptions or misinformation, deploying trusted mediators, promoting proactive and strong provider recommendation, and implementing practical solutions to address missed opportunities (eg, bundling HPV vaccination with other services) and for mobile migrants (eg, eHealth initiatives). Reducing barriers to access through various novel structural measures was recommended, alongside interventions addressing multilevel issues, innovative vaccination monitoring, and affordable or free vaccination.

Interventions specific to subpopulation groups included Halal vaccine certification for Muslims and so-called photonovela, radionovela, or telenovela platforms for Latinx populations. Strategies to address barriers (eg, logistical challenges and a lack of vaccination records or difficulty of follow-up) unique to migrant farm workers are workable in mobile population groups, including hard-to-reach groups. Sending mobile clinics (including culturally and linguistically sensitive outreach workers^71^) to shelters or camps and the involvement of nurses in on-site education and vaccinations (eg, at the workplaces of migrant farm workers)^63^ could address accessibility issues specific to migrants.

There is a paucity of studies in LMICs, especially in low-income countries with limited vaccine availability. Behavioural and social factors influencing vaccine uptake are not systematically collected, and current methods lack standardisation, making cross-study comparison difficult. Global and regional efforts are needed to standardise data collection as part of routine reporting. Such data are important for migrants, for whom vaccine entitlement does not guarantee uptake. Logistical challenges including vaccine supply chains, funding, and political support need attention, as well as tools to navigate vaccine access for newcomers. Exploring the sharing of electronic health records—including digital vaccination histories and key information (eg, contacts, country of origin, race and/or ethnicity, and migrant status for mobile migrants)—and harmonising relevant regional and global legislation and infrastructure could improve service delivery. It is important to consider integrating national statistics on migrant status into vaccination registries for better global monitoring. Calls have been made to go beyond educational interventions alone or a one-level approach.^156^ Studies exploring interventions and their implementation are scarce, and the actual empirical effect of interventions remains underdocumented. Future implementation research should explore successful models from other vaccination programmes—including co-design approaches,^157^ COVID-19 delivery models,^158^ and equitable vaccination strategies^159^—to adapt them for HPV vaccination. Given the current evidence on barriers and facilitators to screening in migrants,^160^ addressing targeted screening and vaccination programmes for migrants should be prioritised.

The strengths of this review include providing a comprehensive understanding of the factors influencing HPV vaccine uptake among migrants, using an established framework and highlighting successful strategies informed by stakeholder recommendations. Our review addresses less prominent and inconclusive factors that are overlooked in literature, illustrating the complexity of uptake drivers and their overlap with sociodemographic factors. We examined programme design and delivery methods, showing how venue, timing, and legal status affect vaccine uptake.

Several limitations should be considered. First, the great majority of studies were from high-income countries, particularly the USA; this would have created a bias, potentially weighing disproportionately towards particular influencing factors (eg, cultural beliefs in specific migrant groups predominantly residing in that geographical area and practical issues specific to US health systems) and subsequent recommended strategies. Second, although inherent to migrant health research, the definition of migrant varied across studies, complicating meta-analyses or subgroup analyses. Third, our meta-analysis results had high heterogeneity (*I*^2^>50%), possibly due to differences in migrant types or HPV vaccination programmes in study countries. Further sensitivity analysis was not possible owing to the small number of studies. Fourth, we found no relevant studies on migrants from the LGBTQ+ community or migrant sex workers. Family reunification data were rarely found; registry-based studies do not always include these data alongside other types of residence permit held by the migrant (eg, refugee, labour migrant, or student).^38^ Data on visa transitions can be incomplete.^46^ Fifth, expansion of target groups since the initiation of HPV vaccine delivery (2006 in the USA) is likely to have contributed to the disproportionate focuses on girls and women rather than boys and men. Additionally, studies focused on different age groups, which could have led to bias in the interpretation. Finally, HPV vaccine delivery and uptake could have been strengthened by improved organisational health literacy; this can be achieved through strong organisational leadership to nurture a culture for the improvement of organisational health literacy and by the implementation of organisational structures and policies aimed at effective cross-cultural communication, better patient navigation of health-care systems, and culturally appropriate strategies to support HPV vaccination as primary prevention.^161^

In conclusion, this review highlights that, despite global commitments to equitable vaccination, persistent social, behavioural, and systemic barriers hinder HPV vaccine uptake among migrants. Although numerous studies were identified, migrants in LMICs are under-represented, risking their exclusion from efforts to eliminate preventable cervical cancers. It is essential to prioritise these populations in research, identify key drivers of uptake, and collaborate with migrant communities to create tailored, effective delivery models that meet their specific needs.

## Supplementary Material

Figure S1, S2, S3, S4; Table S1, S2, S3, S4, S5, S6

## Figures and Tables

**Table:** Characteristics of included studies

	Number of studies (n=117)
Study design	
Quantitative, cross-sectional	62
Qualitative	28
Quantitative, cohort	16
Mixed-methods	10
Randomised controlled trial	1
Year of publication	
2007	1
2009	1
2010	2
2011	3
2012	5
2013	8
2014	3
2015	7
2016	8
2017	9
2018	13
2019	8
2020	7
2021	13
2022	13
2023	11
2024*	5
Host countries	
USA or US territory	77, including 1 in Puerto Rico
Denmark	10
Canada	5
Norway	4
UK	4, including 2 in England and 1 in Scotland
Germany	3
Sweden	2
Poland	2
Italy	2
Australia	2
Netherlands	1
New Zealand	1
South Korea	1
Nepal	1
Malaysia	1
United Arab Emirates	1
WHO region	
Region of the Americas	82
European region	28
Western Pacific region	5
Eastern Mediterranean region	1
South-East Asia region	1
Host country classification by income level	
High income	115
Upper middle income	1
Low middle income	1
Study setting	
Community setting only	47
Household setting only	37
Clinic or hospital setting only	11
College or university setting only	8
Both community and clinic settings	7
Both clinic and household settings	2
Multilevel or multisystems setting	2
Camp	2
Both clinic and high school settings	1
Participant groups	
Recipient of vaccine or those eligible for the vaccine only	34
Parents, caregivers, or guardians only	21
Mother only	17
Father only	1
Health-care provider only	3
Other stakeholders	1
Combination of stakeholders	40
Recipient of vaccine or those eligible for the vaccine and parent or caregiver (including mother only)	34
Parent or caregiver (including mother only) and health-care provider	3
Recipient of vaccine or those eligible for the vaccine and regional coordinators	1
Recipient of vaccine or those eligible for the vaccine, parent or caregiver (including mother only), and health-care provider	2
Number of participants	5 638 838
Number of migrantsf	933189
Sex of participants	
Female only	51
Male and female	49
Male and female or female only (depending on the group)	13
Male only	3
Not available	1
WHO region of origin of participants^‡^	
Region of the Americas	105
European region	85
African region	74
South-East Asia region	59
Western Pacific region	56
Eastern Mediterranean region	49
Type of migrant	
Refugee	13
Student	7
Migrant farm worker	3
Economic migrant	1
Not specified or unclear	93
HPV vaccine uptake (≥1 dose) in migrants§¶	
Female migrants	23·0% (10·0–44·4); *I*^2^=99·3%; n=7614; 7 studies
Male migrants	21·0% (5·0–58·0); *I*^2^=99·3%; n=2764; 3 studies
Male and female migrants combined	17·0% (8·0–33·0); *I*^2^=98·0%; n=3583; 10 studies
Risk of bias	
Low	79
Moderate	32
High	6

HPV=human papillomavirus.

* Up to Dec 4, 2024.

† Includes first-generation and second-generation migrants.

‡ Data are the number of codes (ie, countries of origin reported in each study). The total does not sum to 117 because migrants with more than one country of origin were included in the studies.

§ Data are pooled estimate (95% CI).

¶ Includes data on first-generation migrants from the past decade of studies (2014 onwards) only, in which vaccination status was self-reported in 15 studies and obtained from the national vaccination register in one study. 16 unique studies were included in the meta-analysis, but some provided data for more than one sex. For other HPV vaccination rates in individual studies, see appendix (pp 14–97).

## Data Availability

Data are available upon reasonable request to the corresponding author.

## Research in context

### Evidence before this study

Since its inception in 2006, 141 countries worldwide have introduced human papillomavirus (HPV) vaccination into their Essential Programmes on Immunization. WHO’s Cervical Cancer Elimination Initiative has set a target of 90% HPV vaccination coverage among girls by the age of 15 years by 2030. However, progress towards this target has been slow, with only 27% global coverage in 2023, and lower coverage particularly in low-income and middle-income countries (LMICs) where access to the vaccine remains limited. Globally, migrants are considered an undervaccinated population owing to well documented barriers to accessing vaccination services and inconsistent inclusion in national vaccination policies, despite WHO’s Immunization Agenda 2030, which calls for universal and equitable access to vaccination for all groups across the life course. We searched seven databases (MEDLINE, Embase, American Psychological Association PsycINFO, Global Health, Cumulative Index to Nursing and Allied Health Literature, Scopus, and the Cochrane Library [Cochrane Database of Systematic Reviews and Cochrane Central Register of Controlled Trials]) and grey literature (eg, WHO; Gavi, the Vaccine Alliance; International Organization for Migration; and United Nations High Commissioner for Refugees), without language restrictions, for global data published between Jan 1, 2006, and Dec 4, 2024. The search combined free-text and subject heading terms for migrant, vaccination, and HPV. Our search identified 1806 database records and 1756 records from websites, from which we included 117 studies involving 933 189 first-generation and second-generation migrants across 16 countries and one territory. We extracted data for a hybrid thematic analysis using an adapted version of the WHO-endorsed Behavioural and Social Drivers of Vaccination (BeSD) framework, and conducted a random-effects meta-analysis to calculate pooled estimates of HPV vaccine uptake in first-generation migrants, focusing on studies conducted in the past decade (ie, from 2014 onwards). Risk of bias was assessed using relevant critical appraisal tools from the Joanna Briggs Institute. Before this review, multiple studies in Europe and the Americas indicated higher HPV infection rates and higher cervical cancer incidence among migrants than among their host counterparts. Additionally, in 2024, a global systematic review of two databases by Graci and colleagues (involving 31 442 participants in 34 studies across the WHO region of the Americas and the European and Western Pacific regions) found low rates of HPV vaccination initiation among migrants and refugees (31·6% [95% CI 22·3–40·9]). Other studies, mostly from high-income countries, highlight various obstacles to HPV vaccination among migrants; however, comprehensive data on the drivers of HPV vaccination uptake among migrant groups globally are scarce, and there is a particular shortage of studies mapping approaches, strategies, and best practices to promote uptake in this population.

### Added value of this study

This large-scale, global systematic review and meta-analysis provided low pooled estimates of HPV vaccine uptake in first-generation migrants: 23·0% (95% CI 10·0–44·0; n=7614; *I*^2^=99·3%) for female migrants, 21·0% (5·0–58.0; n=2764; *I*^2^=99·3%) for male migrants, and 17·0% (8·0–33·0; n=3583; *I*^2^=98·0%) for male and female migrants combined. We used the WHO BeSD model to examine positive and negative factors affecting the uptake of HPV vaccination in migrants and explore facilitators to increasing vaccine coverage in these populations. The adapted BeSD model provided a holistic view of uptake drivers among migrants, which could be used to guide research on the topic, particularly for LMICs where data are scarce and vaccine accessibility is constrained. This study has added value not only in synthesising evidence on drivers but also in extracting actionable recommendations drawn from the insights of researchers, migrant communities, health-care providers, and other stakeholders. Key findings revealed complex barriers, such as safety concerns, cultural beliefs, poor knowledge of HPV and/or the HPV vaccine, family dynamics, gender norms, language issues, vaccine cost, and information on services. Facilitators included receiving reliable information and trusted recommendations (particularly through culturally sensitive communication), framing messages with risk awareness and health promotion, and aligning with parents’ values. Recommended strategies and interventions to promote vaccine uptake were diverse, including bundling HPV vaccination with other services; tailoring communication to diverse audiences; engaging trusted community mediators; improving patient–provider, parent–provider, or parent–child interactions; integrating migrants into national vaccination monitoring systems; and offering outreach and proximity services. These insights provide a foundation for targeted interventions to improve HPV vaccination among migrants and guide future research in LMICs.

### Implications of all the available evidence

Migrants globally face multilevel barriers to accessing HPV vaccination. From a policy and practice perspective, meeting the global HPV vaccination targets will require addressing the persistent barriers through the approaches and strategies identified. These efforts should be accompanied by inclusive national and regional initiatives to monitor progress towards HPV vaccination targets and equitable immunisation.

## Panel Approaches and strategies to promote HPV vaccination uptake in migrants

### Implementing effective health information, education, and communication

- Appropriate messaging, formats, and channels
  - Provide culturally and linguistically tailored messages in migrants’ native language, focusing on strength framing, heightening risk perception, or stressing as health promotion, and aligning with parents’ and caregivers’ values (P, A)
  - Offer clear and balanced information on benefits versus risks (eg, vaccine benefits *vs* risks of HPV infection; A)
  - Target all stakeholder groups (eg, beneficiaries, parents and caregivers, and providers; P, A)
  - Use suitable formats for young adults, such as infographics, statistics, narrative videos, and content in native languages on television or radio (P, A)
  - Use suitable formats for adolescents, such as comic books (A)
  - Use suitable formats for parents, such as radio programmes, photonovelas or radionovelas, interactive forums, adverts, and pamphlets (P, A)

- Leveraging trusted mediators
  - Use trusted messengers to reach diverse groups (P, A)
  - Involve peer health promoters as mediators in educational settings (P, A)
  - Engage religious champions, community health workers, and key opinion leaders (P, A)
  - Co-design culturally and religiously sensitive messages with community health workers and migrant representatives (A)

- Male-targeted communication interventions
  - Involve male celebrities in health messages (A)
  - Implement school-based programmes for boys to reduce stigma around HPV vaccination (P)

- Barriers addressed (BeSD model)
  - Poor knowledge about HPV and/or HPV vaccines (domain 1)
  - Negative information or misinformation (domain 2)
  - Low confidence in vaccine safety (domain 1)
  - Perceptions of vaccination unnecessary (domain 1)
  - Perceived uncertainty (perceived information needs; domain 1)
  - Cultural and religious beliefs (domain 1)
  - Gender inequality and mother’s lack of autonomy (domain 2)
  - Vaccine hesitancy (domain 3)
  - Language barrier (domains 4 and 5)
  - Receiving non-factual information from health-care providers (domain 2)

### Improving patient–provider, parent–provider, or parent– child communication and interaction

- Encourage providers to actively promote HPV vaccination with appropriate framing (eg, aligning with parental values and normalising vaccination; P, A)
- Use strong and clear recommendations (P, A)
- Build trust and encourage proactive vaccine behaviour (P, A)
- Provide continuous education for health-care providers on migrant-sensitive and culturally responsive care (A)
- Better message framing for patient–provider, parent–provider, or parent–child communication (eg, emphasising as health promotion and heightening risk perception about partner; P, A)
  - Barriers addressed (BeSD model)
  - Non-willingness of providers to recommend HPV vaccine (domain 3)
  - Receiving non-factual information from health-care providers (domain 2)
  - Low confidence in vaccine safety (domain 1)
  - Cultural and religious beliefs (domain 1)
  - Poor knowledge of HPV and/or HPV vaccines (domain 1)
  - Vaccine hesitancy (domain 3)

### Addressing accessibility issues

- Proximity and free services
  - Provide walk-in, mobile clinics, community pharmacies, and transportation to clinics (P, A)
  - Invest in outreach services and on-site vaccination (P)
  - Provide publicly funded free HPV vaccination (P, A)
  - Invest in safety-net clinics (A)
  - Provide high-quality interpretation services and multilingual providers at service points (P, A)

- Bundling and using other health-care visits
  - Bundle HPV vaccination with other services or campaigns (eg, HIV programmes, school curricula, and sexual education; P, A)
  - Capitalise on opportunistic vaccination by making use of other health-care visits (eg, general practitioner visits, maternal care, gynaecology visits, and paediatric visits) and existing school-based programmes to provide catch-up vaccination (A)

- Barriers addressed (BeSD model)
  - Not knowing where to go to receive an HPV vaccine (domain 4)
  - Difficulty of follow-up or loss to follow-up (domain 4)
  - Cost of vaccines (domain 4)
  - Language barrier (domains 4 and 5)
  - High migrant mobility (domain 5)

### Strengthening vaccination monitoring

- Establish national, long-term monitoring systems for vaccine uptake, including data on the migrants’ country of origin, their migratory status, and their ethnicity (A)
- Implement innovative strategies for mobile groups (eg, electronic vaccination booklets or records and eHealth tools; P, A)
- Implement reminder systems (eg, text messages, telephone calls, letters, and hotlines; P, A)
- Barriers addressed (BeSD model)
  - Lack of vaccination records or difficulty of follow-up (domain 4)
  - High migrant mobility (domain 5)

A=authors’ recommendations or suggestions. BeSD=Behavioural and social drivers of vaccination. HPV=human papillomavirus. P=participants’ recommendations.
