# Supplementary material for: Drivers of human papillomavirus vaccine uptake in migrant populations and interventions to improve coverage: a systematic review and meta-analysis
Source: Lancet Public Health. Author manuscript; Available in PMC 2026 Feb 19. (PMC7618757; doi:10.1016/S2468-2667(25)00148-3)
Supplement: Figure S1, S2, S3, S4; Table S1, S2, S3, S4, S5, S6 [file EMS212115-supplement-Figure_S1__S2__S3__S4__Table_S1__S2__S3__S4__S5__S6.pdf]

THE LANCET
Public Health

## Supplementary appendix

This appendix formed part of the original submission and has been peer reviewed. We post it as supplied by the authors.

Supplement to: Iwami M, Bouaddi O, Razai MS, et al. Drivers of human papillomavirus vaccine uptake in migrant populations and interventions to improve coverage: a systematic review and meta-analysis. *Lancet Public Health* 2025; **10:** e693–711.

**Supplementary appendix**

# Drivers of human papillomavirus vaccine uptake in migrant populations and interventions to improve coverage: a systematic review and meta-analysis

**Authors**

Michiyo Iwami, PhD†[1], Oumnia Bouaddi, MD†[2,3,4], Mohammad S Razai, MD Res.[1,5], Rania Mansour, MD[1,6], Beatriz Morais, MSc[7], Nafeesa Mat Ali, MBBS[7], Alison F Crawshaw, PhD[1,8], Sainabou Bojang, MSc[1], Farah Seedat, PhD[1], Anna Deal, MSc[1], Sophie Webb, BMBS[7], Jessica Carter, MBBS[1,9], Nathaniel Aspray, MBChB[1], Nuria Sanchez Clemente, PhD[1], Juan Arroyo-Laguna, PhD[10], Sanjeev Krishna, ScD[7,11], Yolanda Augustin, PhD††[7], Henry M Staines, DPhil††[7], Sally Hargreaves, PhD††*[1]

**Affiliations**

[1]The Migrant Health Research Group, Institute for Infection and Immunity, School of Health and Medical Sciences, City St George's, University of London, London, United Kingdom

[2]Mohammed VI International School of Public Health, Mohammed VI University of Sciences and Health, Casablanca, Morocco

[3]Department. of Public Health and Clinical Research, Mohammed VI Center for Research and Innovation, Rabat, Morocco

[4]Barcelona Institute for Global Health (ISGlobal, Hospital Clinic – University of Barcelona), Barcelona Spain

[5]Primary Care Unit, Department of Public Health and Primary Care, University of Cambridge, Cambridge, United Kingdom

[6]Department of General Surgery, Mayo Clinic, Phoenix, Arizona, United States of America.

[7]Institute for infection and Immunity, School of Health and Medical Sciences, City St George's, University of London, London, United Kingdom

[8]Prevention, Inequalities and Commissioning, City and Hackney Public Health Team, Hackney Council, 1 Hillman Street, London, United Kingdom

[9]Wolfson Institute of Population Health, Queen Mary's University of London, London, United Kingdom

[10]Institute of Social Analytics and Strategic Intelligence Pulso PUCP, Faculty of Social Sciences, Pontifical Catholic University of Peru, Lima, Peru

[11]Institut Für Tropenmedizin, Eberhard Karls Universität Tübingen, and German Center for Infection Research (Dzif), Tübingen, Germany

†Joint first authors

††Joint last authors

***Corresponding author:** Prof Sally Hargreaves. City St George's, University of London, United Kingdom, SW17 0EA. Email: s.hargreaves@sgul.ac.uk

**Contents**


Supplementary appendix ........ 1

Figure S1. The WHO Behavioural and Social Drivers of Vaccination Uptake Framework ...... 3
Figure S2. Map of study location ........ 4
Figure S3. Sensitivity analysis results – Forest Plots ........ 5
Figure S4. Coding results (frequency counts). N=117 studies ........ 6
Table S1. Inclusion and exclusion criteria ........ 7
Table S2. Search strategy MEDLINE (Ovid) (1 January 2006 – 4 December 2024) ........ 8
Table S3. Country/region of origin of participants (117 studies, 5,638,838 participants) ........ 10
Table S4. Race/ethnicity of participants (117 studies, 5,638,838 participants) ........ 13
Table S5. Detailed description of included studies (N=117) ........ 14
Table S6. A summary of qualitative and quantitative findings ........ 98
References ........ 106

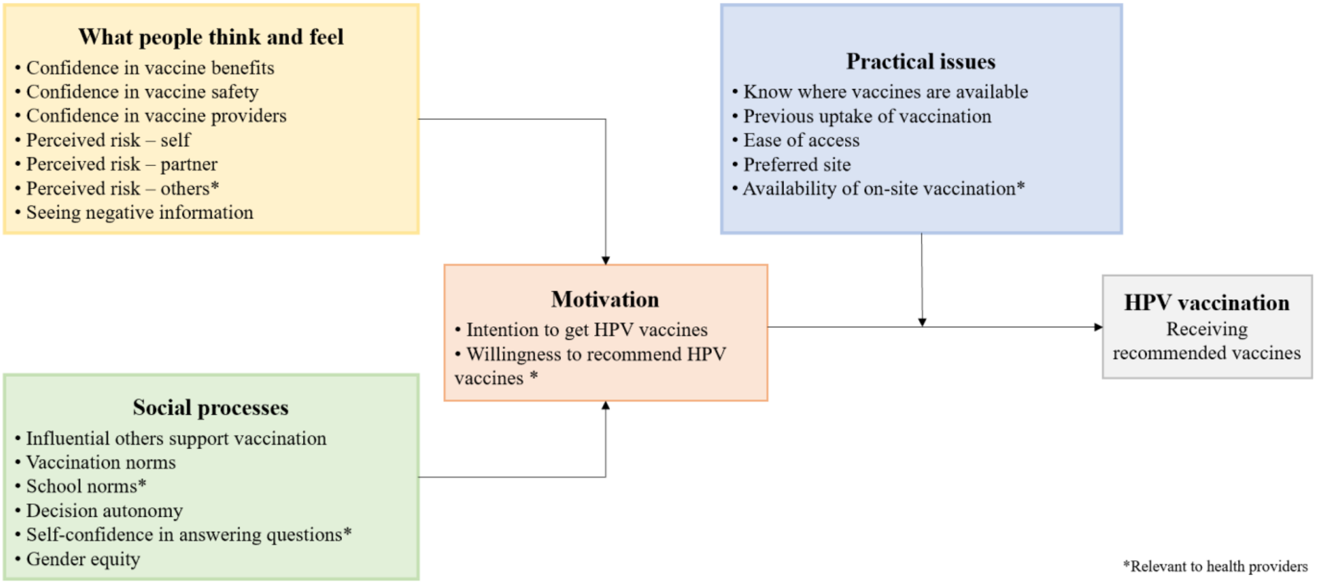


**Figure S1. The WHO Behavioural and Social Drivers of Vaccination Uptake Framework**

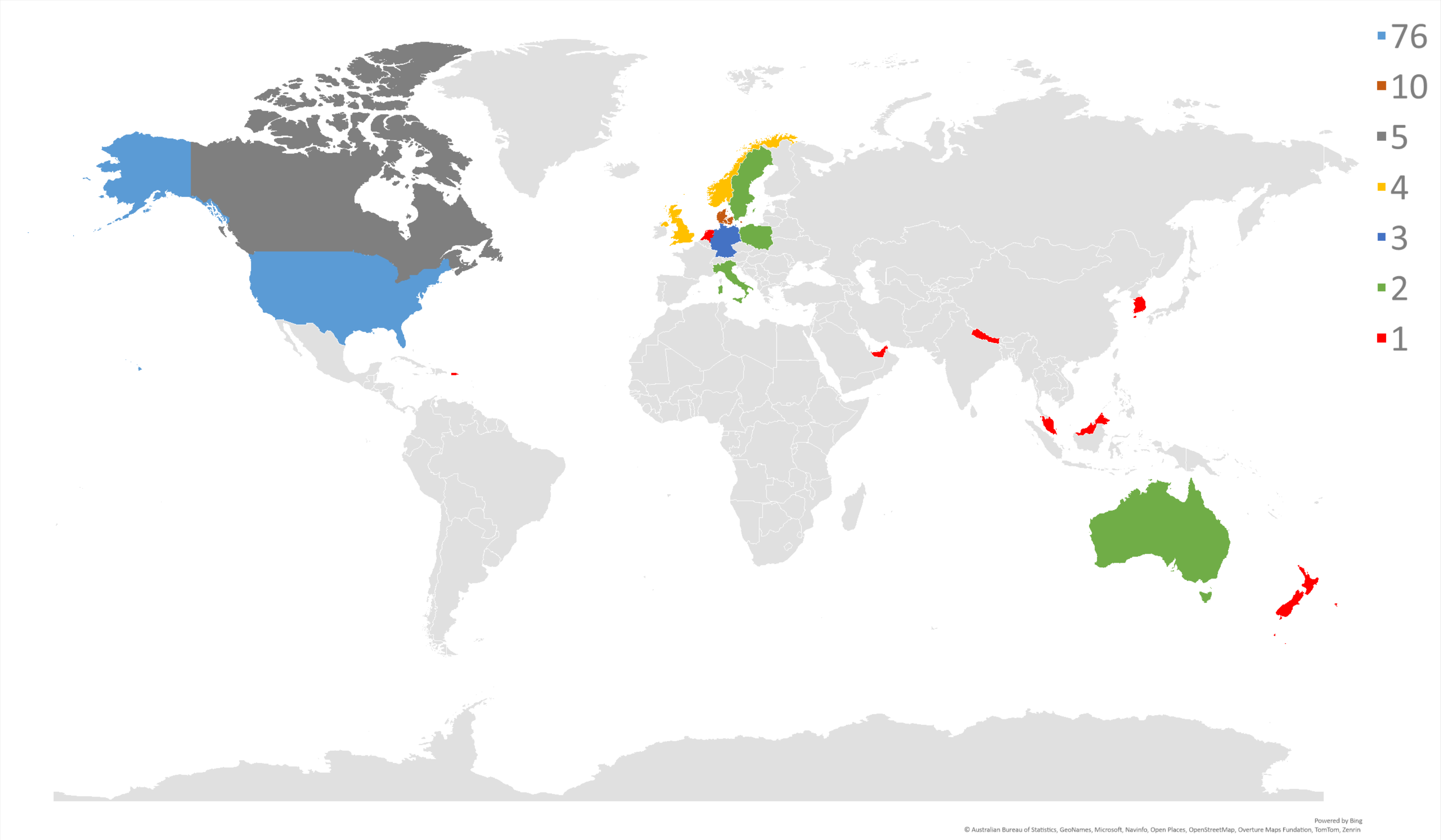


**Figure S2. Map of study location**

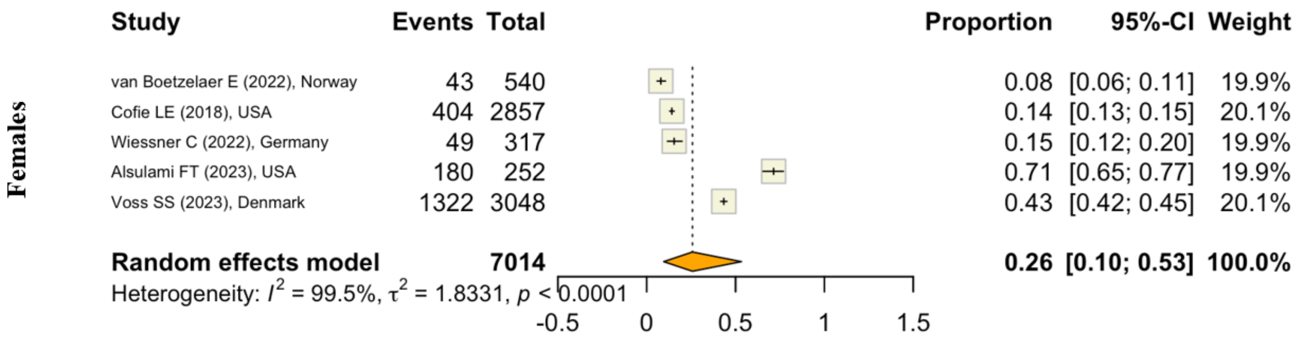


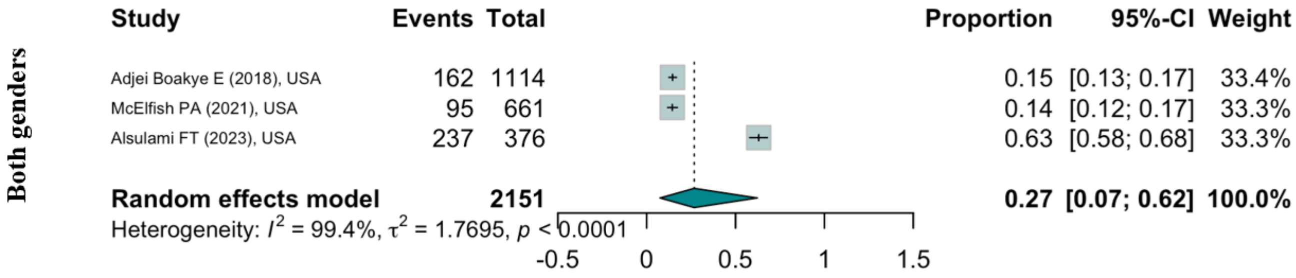


**Figure S3. Sensitivity analysis results – Forest Plots**

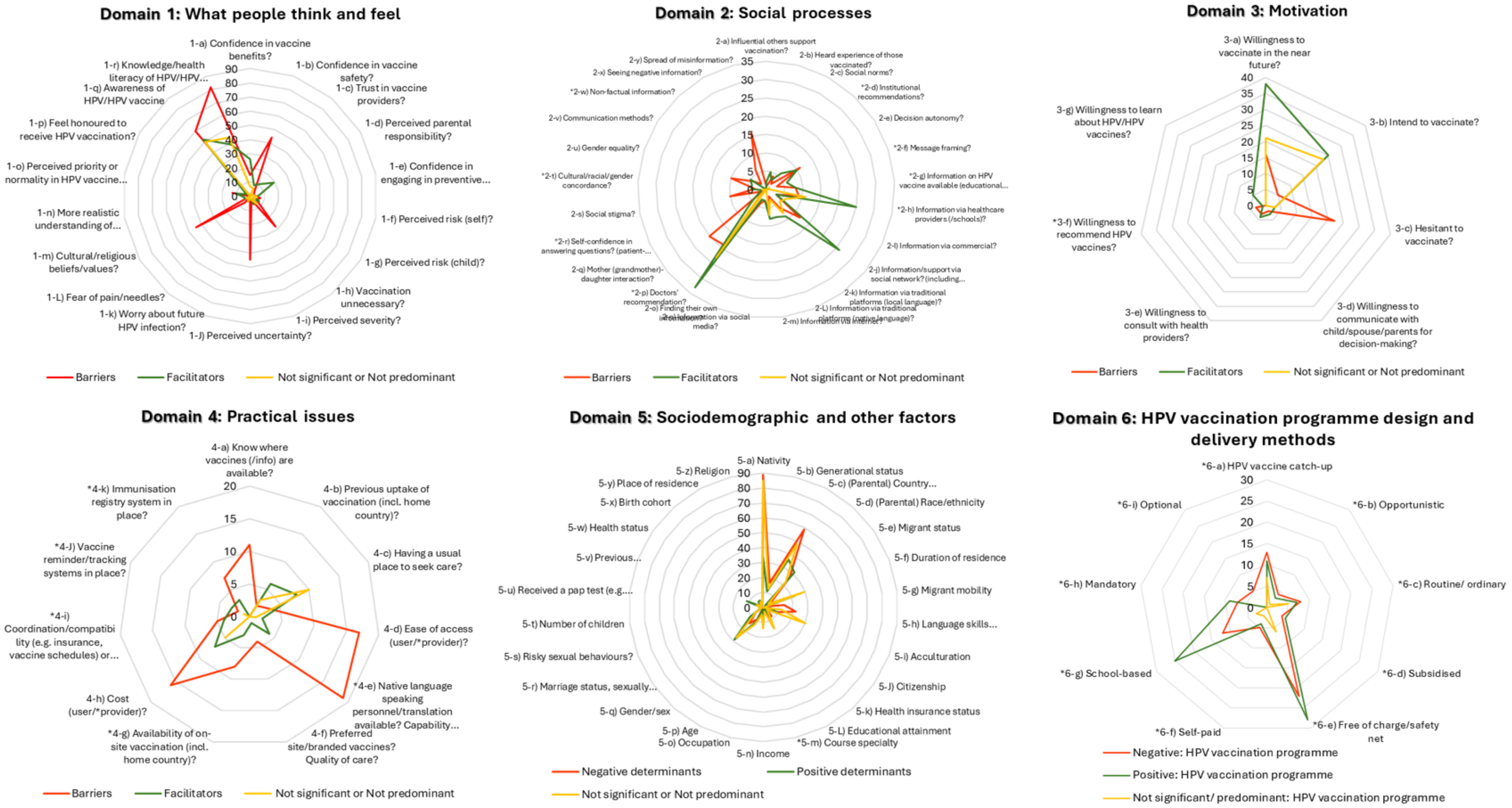


**Figure S4. Coding results (frequency counts). N=117 studies**

**Table S1. Inclusion and exclusion criteria**

| Inclusion criteria | Exclusion criteria |
|---|---|
| • Published from 1 January 2006 onwards; any language; any geographical regions/ areas.<br>• Community (including a humanitarian setting) and/or clinical settings.<br>• Adolescent and adult migrants (defined as foreign-born) and children of migrant parents who are eligible for HPV vaccination programme irrespective of gender/sex, sexuality, migratory status, and socioeconomic status.<br>• Studies on other stakeholders' views/perceptions about barriers/ facilitators to uptake (/service delivery) of HPV vaccines in (/for) migrants.<br>• Qualitative studies, observational studies (e.g. cohort/ cross-sectional/ case-control/ longitudinal studies; case studies; natural experiments, quasi experiments), randomised controlled trials (RCTs), and before-after studies, which reported primary data on negatively or positively influencing factors to the uptake (/service delivery) of HPV vaccines among migrants globally.<br>• Secondary data analysis, as long as meeting the above criteria.<br>• Studies that migrants were analysed as sub-group, as long as meeting the above criteria.<br>• Studies that HPV vaccination were included in wider vaccination/ vaccine studies, as long as meeting the above criteria.<br>• Grey literature, including theses, dissertation, non-peer-reviewed literature. | • Internal migrants.<br>• Migrant clinicians.<br>• Animals (e.g. migrant animals are excluded).<br>• Editorials, opinion pieces, commentaries, protocols), policy documents, and guidelines; conference abstracts/ posters/ proceedings, case (series) reports, systematic/scoping/rapid/umbrella reviews.<br>• Economic studies.<br>• Not transparently report on migrants or migratory status.<br>• Out of scope - Reports that were thoroughly about other vaccines (e.g. flu, MMR) but did not include HPV vaccines, or not about HPV vaccines for prophylactic use but only for therapeutic use.<br>• Out of scope - Focusing on factors that influenced uptake of cervical/ anal/ oral cancer screening, HPV testing, self-sampling, HPV infection rates, ethics of HPV vaccines etc, but not thoroughly about factors that influenced uptake (/service delivery) of HPV vaccination.<br>• Out of scope - Reporting on vaccine design/ development/ production, or tool development (including survey tools).<br>• Out of scope - Reporting on lab studies, sero-surveillance, molecular virology, immunology, biological assays, genome studies.<br>• Not available on internet, beyond institutional access, or full-text is not retrievable.<br>• Duplicate. |

**Table S2. Search strategy MEDLINE (Ovid) (1 January 2006 – 4 December 2024)**

| No. | Database/ interface | Search date | Search string | No. of records yielded |
|---|---|---|---|---|
| 1-a | Ovid Medline | 2 March 2022 | exp Vaccines/ or vaccin*.mp. or exp Vaccination/<br>immunis*.mp.<br>exp Immunization/ or exp Immunization Programs/ or immuniz*.mp.<br>exp Papillomavirus Infections/ or exp Papillomaviridae/ or human papilloma*.mp.<br>HPV.mp.<br>exp Papillomavirus Vaccines/<br>1 or 2 or 3<br>4 or 5<br>7 and 8<br>6 or 9<br>exp "Transients and Migrants"/ or migrant*.mp.<br>exp "Emigrants and Immigrants"/ or immigrant*.mp.<br>emigrant*.mp.<br>transient*.mp.<br>exp Refugees/ or refugee*.mp.<br>asylum seeker*.mp.<br>foreign*.mp.<br>born overseas.mp.<br>displaced.mp.<br>undocumented.mp.<br>newcomer*.mp.<br>new comer*.mp.<br>expat*.mp.<br>diaspora*.mp.<br>non-resident*.mp.<br>non-citizen*.mp.<br>newly arrived.mp.<br>new arrival*.mp.<br>non-national*.mp.<br>11 or 12 or 13 or 14 or 15 or 16 or 17 or 18 or 19 or 20 or 21 or 22 or 23 or 24 or 25 or 26 or 27 or 28 or 29<br>10 and 30<br>limit 31 to yr="2006 -Current" | 349 |
| 1-b | *ditto* | 3 March 2023 | exp Vaccines/ or vaccin*.mp. or exp Vaccination/<br>immunis*.mp.<br>exp Immunization/ or exp Immunization Programs/ or immuniz*.mp.<br>exp Papillomavirus Infections/ or exp Papillomaviridae/ or human papilloma*.mp.<br>HPV.mp.<br>exp Papillomavirus Vaccines/<br>1 or 2 or 3<br>4 or 5<br>7 and 8<br>6 or 9<br>exp "Transients and Migrants"/ or migrant*.mp.<br>exp "Emigrants and Immigrants"/ or immigrant*.mp.<br>emigrant*.mp.<br>transient*.mp.<br>exp Refugees/ or refugee*.mp.<br>asylum seeker*.mp.<br>foreign*.mp.<br>born overseas.mp.<br>displaced.mp.<br>undocumented.mp.<br>newcomer*.mp.<br>new comer*.mp.<br>expat*.mp.<br>diaspora*.mp.<br>non-resident*.mp.<br>non-citizen*.mp.<br>newly arrived.mp. | 50 |

| No. | Database/ interface | Search date | Search string | No. of records yielded |
|---|---|---|---|---|
| | | | new arrival*.mp.<br>non-national*.mp.<br>11 or 12 or 13 or 14 or 15 or 16 or 17 or 18 or 19 or 20 or 21 or 22 or 23 or 24 or 25 or 26 or 27 or 28 or 29<br>10 and 30<br>limit 31 to yr="2022 -Current" | |
| 1-c | *ditto* | 4 December 2024 | exp Vaccines/ or vaccin*.mp. or exp Vaccination/<br>immunis*.mp.<br>exp Immunization/ or exp Immunization Programs/ or immuniz*.mp.<br>exp Papillomavirus Infections/ or exp Papillomaviridae/ or human papilloma*.mp.<br>HPV.mp.<br>exp Papillomavirus Vaccines/<br>1 or 2 or 3<br>4 or 5<br>7 and 8<br>6 or 9<br>exp "Transients and Migrants"/ or migrant*.mp.<br>exp "Emigrants and Immigrants"/ or immigrant*.mp.<br>emigrant*.mp.<br>transient*.mp.<br>exp Refugees/ or refugee*.mp.<br>asylum seeker*.mp.<br>foreign*.mp.<br>born overseas.mp.<br>displaced.mp.<br>undocumented.mp.<br>newcomer*.mp.<br>new comer*.mp.<br>expat*.mp.<br>diaspora*.mp.<br>non-resident*.mp.<br>non-citizen*.mp.<br>newly arrived.mp.<br>new arrival*.mp.<br>non-national*.mp.<br>11 or 12 or 13 or 14 or 15 or 16 or 17 or 18 or 19 or 20 or 21 or 22 or 23 or 24 or 25 or 26 or 27 or 28 or 29<br>10 and 30<br>limit 31 to yr="2023 -Current" | 66 |

**Table S3. Country/region of origin of participants (117 studies, 5,638,838 participants)**

| WHO region | Country/ Region of Origin (Frequency counts) |
|---|---|
| Region of the Americas [AMR] (105) | **Mexico (17)**<br>LAC (9)<br>Canada (5)<br>Dominican Republic (5)<br>El Salvador (5)<br>Peru (5)<br>USA (5)<br>South America (5)<br>Haiti (4)<br>Colombia (4)<br>Honduras (4)<br>Guatemala (4)<br>Brazil (3)<br>Central America (3)<br>Ecuador (2)<br>Cuba (2)<br>Latin America (2)<br>Argentina (2)<br>North America (2)<br>Antigua (1)<br>Bahamas (1)<br>Barbados (1)<br>Trinidad (1)<br>Dominica (1)<br>Costa Rica (1)<br>Nicaragua (1)<br>Caribbean (1)<br>Antilles (1)<br>Suriname (1)<br>Panama (1)<br>Venezuela (1)<br>Puerto Rico (1)<br>Northern America (1)<br>Southern America (1)<br>Chile (1)<br>America (1) |
| European Region [EUR] (85) | Europe (6)<br>Eastern Europe (6)<br>Turkey (5)<br>Russia (5)<br>Poland (5)<br>Ukraine (3)<br>Central Asia (3)<br>Western Europe (3)<br>Germany (3)<br>Sweden (3)<br>Northern Europe (2)<br>France (2)<br>Latvia (2)<br>Spain (2)<br>Greenland (2)<br>Romania (2)<br>Lithuania (2)<br>Bosnia-Herzegovina (2)<br>Western countries (2)<br>Bosnia (1)<br>Denmark (1)<br>EU (1)<br>Europe outside EU (1)<br>Former-USSR (1)<br>Southern Europe (1)<br>Kazakhstan (1)<br>Portugal (1)<br>Europe Eurasia (1)<br>Old EU (1)<br>EEA-EFTA (1)<br>newer EU (1)<br>Finland (1)<br>Serbia (1)<br>United Kingdom (1)<br>Israel (1)<br>Italy (1)<br>Malta (1)<br>Netherlands (1) |

| WHO region | Country/ Region of Origin<br>(Frequency counts) |
|---|---|
| | Norway (1)<br>Faroe Islands (1)<br>Former Yugoslavia (1)<br>Central Europe (1)<br>Iceland (1)<br>Bulgaria (1) |
| African Region [AFR]<br>(74) | Africa (14)<br>**Somalia (9)**<br>Sub-Saharan Africa (7)<br>Ethiopia (5)<br>Eritrea (4)<br>Nigeria (4)<br>Tanzania (3)<br>Egypt (3)<br>Morocco (3)<br>Kenya (3)<br>Zimbabwe (2)<br>South Africa (2)<br>North Africa (2)<br>Cape Verde (1)<br>Algeria (1)<br>Zambia (1)<br>African country (1)<br>Burundi (1)<br>Congo (1)<br>Rwanda (1)<br>Liberia (1)<br>East Africa (1)<br>Senegal (1)<br>Ivory Coast (1)<br>Mauritius (1)<br>Malawi (1) |
| South-East Asian Region [SEAR]<br>(59) | Asia (11)<br>India (8)<br>Vietnam (7)<br>Southeast Asia (7)<br>South Asia (6)/ Southern Asia (1)<br>Bangladesh (3)<br>East Asia (3)<br>Sri Lanka (2)<br>Bhutan (2)<br>Indian subcontinent (2)<br>Nepal (2)<br>Eastern Asia (2)<br>East India (1)<br>Thailand (1)<br>Indonesia (1) |
| Western Pacific Region [WPR]<br>(56) | **China (12)**<br>South Korea (7)<br>Australia (5)<br>Philippines (5)<br>Oceania (4)<br>New Zealand (3)<br>Cambodia (2)<br>Japan (2)<br>Laos (1)<br>Pacific Islands (1)<br>Tonga (1)<br>Samoa (1)<br>Vanuatu (1)<br>Pacific (1)<br>Taiwan (1)<br>Tibet (1)<br>Fiji (1)<br>Hong Kong (1)<br>Malaysia (1)<br>Singapore (1)<br>Brunei (1)<br>Papua New Guinea (1)<br>Micronesia/ Melanesia (1)<br>Polynesia (1) |

| WHO region | Country/ Region of Origin (Frequency counts) |
|---|---|
| Eastern Mediterranean Region [EMR] (49) | Pakistan (7)<br>Iraq (6)<br>Lebanon (5)<br>Middle East (5)<br>Syria (4)<br>MENA (3)<br>Iran (3)<br>Afghanistan (3)<br>Palestine (2)/ Stateless Palestinian (1)<br>Western Asia (2)<br>Jordan (1)<br>Yemen (1)<br>West Asia (1)<br>Somalia (1)<br>Libya (1)<br>Sudan (1)<br>Kuwait (1)<br>Near East (1) |
| Cross-cutting* (2) | Western Hemisphere (1)<br>Non-western countries (1) |
| Not applicable (5) | Unknown (3)<br>Missing (2) |

MENA: Middle East and North Africa; LAC: Latin America and the Caribbean
*Unable to categorise into one single region.

**Table S4. Race/ethnicity of participants (117 studies, 5,638,838 participants)**

| Category | Race/ Ethnicity reported (Frequency counts) |
|---|---|
| Asian<br>(49) | **Asian (15)**<br>Chinese (8)<br>**Other Asian (4)**<br>Korean American (3)<br>Indian (3)<br>Korean (3)<br>**Asian Indian (2)**<br>Filipino (2)<br>Vietnamese (2)<br>Vietnamese American (1)<br>Pakistani (1)<br>Bangladeshi (1)<br>**Non-Hispanic Asian (1)**<br>**South Asian (1)**<br>Japanese (1)<br>Korean Chinese (1) |
| Black<br>(47) | African American (16)<br>**Black (14)**<br>**Non-Hispanic Black (7)**<br>African (6)<br>Oromo (2)<br>Afro-Caribbean (1)<br>Black African (1) |
| Hispanic/ Latino/a/x<br>(34) | **Hispanic (14)**<br>**Latina/o (11)**<br>**Hispanic/ Latino (4)**<br>**Latinx (3)**<br>Mexican American (1)<br>Other Hispanic (1) |
| White<br>(30) | **White (16)**<br>**Non-Hispanic White (10)**<br>Caucasian (1)<br>White British (1)<br>**Non-Latino White (1)**<br>European (1) |
| Indigenous peoples/ natives<br>(23) | American Indian (5) / Native American (2)<br>Alaskan Native (4)<br>NHPI (4)<br>Pacific Islander (2)<br>Māori (1)<br>Pacific (1)<br>Somali (1)<br>Amhara (1)<br>Hadere? (1)<br>Tigre (1) |
| Mixed or Multiple ethnic or racial groups<br>(7) | Multi-racial (7) |
| Gypsy, Roma and Traveller<br>(6) | Romania Roma (1)<br>Slovakian Roma (1)<br>English Gypsy (1)<br>Welsh Gypsy (1)<br>Irish Traveller (1)<br>Scottish Showpeople (1) |
| Middle Eastern<br>(1) | Palestinian (1) |
| Cross-cutting*<br>(6) | Non-Hispanic Other (3)<br>Non-Hispanic (1)<br>Caribbean (1)<br>MELAA (1) |
| Not applicable<br>(6) | Missing (3)<br>Unknown (3) |

MELAA: Middle Eastern, Latin American and African; NHPI: Native Hawaiian & Pacific Islander
*Unable to group into one single category

**Table S5. Detailed description of included studies (N=117)**

| Study ID, First author, Publication year | Study type/ design | Year(s) of study | Location/country of study | Setting | Total number of participants/ sample size (study completers) | No. of immigrants/first-generation & descendants/ second-generation | Proportion of immigrants/first-generation & descendants/second-generation | Migratory status/ Reason for migration | Country/Region of origin/ Nativity/ Ethnicity(/race) | Age | Gender/ sex | Participant type | Intervention/ programme | Uptake of HPV vaccine | Study risk of bias scores |
|---|---|---|---|---|---|---|---|---|---|---|---|---|---|---|---|
| [1], Stephens DP, 2013 | Mixed methods | October 2010 - May 2011 (recruitment) | USA | Community | N=31 (immigrant Haitian mothers, having a daughter between aged 11 and 18, who had not received the HPV vaccine before) | n=31 | 100.0% | (Recent migrants - resided in USA for <5 years) | COUNTRY OF ORIGIN: Haiti (100%) | AGE (Mother): No average age data available.<br>AGE GROUP (Daughter): aged 11-15 (n=13; 41.9%); aged 16-18 (n=18; 58.1%). | Female (100%) | Parent (migrant mother) | n/a | n/a | High risk |
| [2], McFadden SM, 2021 | Mixed methods | December 2017 (provider focus group); July - November 2018 (evaluation of the online CE course) | USA | Community; Clinic (/local hospitals/public health organisations) | FOCUS GROUPS: 11 Providers (4 physicians, 1 nurse practitioner, 5 medical assistants, 1 medical interpreter) + 30 East African immigrant mothers of children aged 11-17.<br><br>EVALUATION OF ONLINE CE COURSE (Pre-test survey only relevant): N=202 providers (158 did follow-up). | n=30 | 73.2% | Not specified | FOCUS GROUPS: 11 Providers who regularly work with East African community members (4 physicians, 1 nurse practitioner, 5 medical assistants, 1 medical interpreter - Data on country/region of origin or ethnicity/race not available) + 30 East African migrant mothers of children aged 11-17 (COUNTRY OF ORIGIN: Somalia 36.7%, Ethiopia 33.3%, Eritrea 30.0%).<br><br>EVALUATION OF ONLINE CE COURSE (Pre-test survey only relevant): N=202 healthcare providers.<br>HISPANIC ETHNICITY: Yes (9.9%); No (90.1%).<br>RACE: White (54.5%); Black (5.5%); Asian (24.3%); Pacific Islander (1.5%); Other (14.4%). | No average age data available. | Both gender (providers); Female (100%, migrant mothers).<br>FOCUS GROUPS: 11 Providers (gender data not available) + 30 East African immigrant mothers of children aged 11-17.<br>EVALUATION OF ONLINE CE COURSE (Pre-test survey only relevant): Female (86.1%); Male (13.4%); Other (0.5%). | Healthcare provider; Parent (migrant mother) | Online continuing education (CE) course aiming to enhance self-efficacy among healthcare providers | n/a | Moderate risk |

| Study ID, First author, Publication year | Study type/ design | Year(s) of study | Location/country of study | Setting | Total number of participants/ sample size (study completers) | No. of immigrants/first-generation & descendants/ second-generation | Proportion of immigrants/first-generation & descendants/second-generation | Migratory status/ Reason for migration | Country/Region of origin/ Nativity/ Ethnicity(/race) | Age | Gender/ sex | Participant type | Intervention/programme | Uptake of HPV vaccine | Study risk of bias scores |
|---|---|---|---|---|---|---|---|---|---|---|---|---|---|---|---|
| [3], Lai D, 2017 | Mixed methods | May - October 2014 (First phase recruitment); October-2014-February 2015 (Second phase of recruitment) | USA | Community | N=228 (survey); n=93 of 228 participants (Focus groups) | n=154 | 67.5% | Refugees (African refugees) are part of ethnic groups studied. | NATIVITY: Foreign-born (67.5%); US-born (31.6%); Missing (0.9%). ETHNICITY: African American (7.5%); African Immigrant (17.1%); American Indian/Alaskan native (10.1%); Hispanic/Latino (28.1%); Native Hawaiian/Pacific Islander (30.7%); Other including multiracial (3.1%); Missing (3.5%). | AGE: Mean 43.09 years (SD=10.19; range 18-74). [SURVEY] AGE GROUP: <35 years old (n=42; 18.4%); 35-50 years old (n=148; 64.9%); >50 years old (n=35; 15.4%); Missing (n=3; 1.3%). | Both gender. Female (71.1%); Male (28.1%); Missing (0.9%). | Parents/legal guardians/ caregivers (aged ≥18, who were vaccination decision-makers for children aged 11-17) | n/a | n/a | Low risk |
| [4], Joseph NP, 2012 | Mixed methods | October 2008-March 2009 (recruited) | USA | Clinic (Paediatric outpatient clinic and an affiliated community health centre) | N=70 (mothers/legal guardians of girls aged 11-17, who visited paediatric and adolescent outpatient clinics at an urban, academic medical centre and an affiliated community health centre in Boston, Massachusetts, between October 2008-March 2009). | n=51 | 72.9% | Not specified | NATIVITY/ETHNICITY/COUNTRY OF ORIGIN: Haitian-born immigrant women (72.9%); US-born African-American women (27.1%). | AGE by Ethnicity (Participating woman): All, Mean 46 years; African American, Mean 42.5 years (SD=8.5); Haitian migrant, Mean 47 years (SD=7.7). AGE by Ethnicity (Daughter): African American, Mean 14.8 years (SD=1.8); Haitian migrant, Mean 13.7 years (SD=2.1). [p=0.03] | Female (100%). | (Grand) Parent (Mother and Grandmother (legal guardians) of girls aged 11-17). Mothers (94.3%); Grandmothers (legal guardians, 5.7%). | Free-of-charge vaccine (through Vaccines for Children and a federally funded vaccine programme) | Self-report: Receipt of HPV vaccine: 31% (migrants: Haitian) vs 47% (non-migrants: African American), p=0.22. <br><br> Electronic Medical Record used to review participants' daughters' HPV vaccination 12 months after enrolment. | Low risk |
| [5], Wilson LA, 2021 | Mixed methods | 2018 (May-July 2018 [surveys]; June-August 2018 [Semi-structured | Canada | Clinic (community health centres) | N=50 (for survey; 7 participants follow-up semi-structured interview) | N=50 (newcomers): Caregivers n=41; Young adults n=9. | 100.0% | Immigrant (40.0%); Refugee (38.0%); Other (18.0%); Prefer not to | REGION OF ORIGIN: Middle East and North Africa (58%); Sub-Saharan Africa (36%); Other (6%). 18 countries in total. | AGE (Young adult, survey only): Mean 22.4 years (SD=3.7; range 17–27); AGE (Caregiver, survey only): Mean 32.0 years (SD=10.1; Range: 21–57). AGE GROUP (survey only): aged | Both gender. Survey (young adults/caregivers): Female (76.0%); Male (24.0%). Follow-up interview (caregivers): Female | Recipient of vaccine (young adults); and Caregivers. | n/a | Self-report (Young adults): Vaccinated 16.7%. | Moderate risk |

| Study ID, First author, Publication year | Study type/ design | Year(s) of study | Location/country of study | Setting | Total number of participants/ sample size (study completers) | No. of immigrants/first-generation & descendants/ second-generation | Proportion of immigrants/first-generation & descendants/second-generation | Migratory status/ Reason for migration | Country/Region of origin/ Nativity/ Ethnicity(/race) | Age | Gender/ sex | Participant type | Intervention/ programme | Uptake of HPV vaccine | Study risk of bias scores |
|---|---|---|---|---|---|---|---|---|---|---|---|---|---|---|---|
| | | interviews] ) | | | | | | say (4.0%). | | 16-27 (26.0%); aged 28-44 (38.0%); aged 45+ (30.0%); Prefer not to say (6.0%). | (71.4%); Male (28.6%). | | | | |
| [6], Perkins RB, 2010 | Mixed methods (mainly qualitative) | June 2007-February 2008 (Interview) | USA | Clinic (urban academic medical centre and an affiliated community health centre) | N=73 (parents/ legal guardians of vaccine-eligible girls [aged 11-18] attending medical appointments in an urban academic medical centre and an affiliated community health centre) | n=33 (no US/US territory-born immigrants) | 45.2% | Not specified | NATIVITY (Parents): US/US territory-born (55%); Foreign-born (45%).<br><br>ETHNICITY/RACE (Parents): Caucasian (26%); African-American (25%); Afro-Caribbean (16%); African (4%); Latino (29%).<br><br>ETHNICITY/RACE (Parents): Caucasian n=19 (US 23%; Bosnia 1%; Canada 1%) = MIX of US-born and Foreign-born; African-American n=18; African n=3 (Cape Verde 1%; Nigeria 1%; Tanzania 1%) = Foreign-born; Afro-Caribbean n=12 (Antigua 1%; Bahamas 1%; Barbados 3%; Haiti 5%; Trinidad 5%) = Foreign-born; Latino n= 21 (Colombia 7%; Dominican Republic 8%; El Salvador 7%; Puerto Rico [US-territory] 4%; US-born 3%) = MIX of US-born and Foreign-born.<br><br>COUNTRY OF ORIGIN (Parents): US-born (51%); Puerto Rico [US-territory] (4%);<br>Bosnia (1%); Canada (1%); Antigua (1%); Bahamas (1%); Barbados (3%); Haiti (5%); Trinidad (5%); Cape Verde (1%); Nigeria (1%); Tanzania (1%); Colombia (7%); Dominican Republic (8%); El Salvador (7%). | AGE (Parent): Mean 43 years (range: 31-60). Note: Data not disaggregated for migrants and non-migrants.<br>AGE (Adolescent girl): Mean 15 years (range: 11-18). Note: Data not disaggregated for parent's nativity. | Both gender. | Parents/ legal guardians of vaccine-eligible girls (aged 11-18): Mother (n=67; 92%); Father (n=3; 4%); Aunt (n=2; 3%); Sister (n=1; 1%). Note: No disaggregated data available for migrants and non-migrants. | School-related mandatory HPV vaccination (scenario) | n/a | Low risk |

| Study ID, First author, Publication year | Study type/ design | Year(s) of study | Location/country of study | Setting | Total number of participants/ sample size (study completers) | No. of immigrants/first-generation & descendants/ second-generation | Proportion of immigrants/first-generation & descendants/second-generation | Migratory status/ Reason for migration | Country/Region of origin/ Nativity/ Ethnicity(/race) | Age | Gender/ sex | Participant type | Intervention/ programme | Uptake of HPV vaccine | Study risk of bias scores |
|---|---|---|---|---|---|---|---|---|---|---|---|---|---|---|---|
| [7], Ayash C, 2022 | Mixed methods | August 2019–April 2021 (recruitment) | USA | Community | N=162 (Arab American migrant women who had ≥1 child aged 9-26 [Qualitative component of the survey, free-response questions]: n=100) | N=162 (Qualitative component of the survey [free-response questions]: n=100) | 100.0% | Not specified | COUNTRY OF ORIGIN (N = 160):<br>Overall: Algeria (2.5%); Egypt (23.8%); Iraq (5.6%); Jordan (6.3%); Lebanon (9.4%); Morocco (9.4%); Palestine (7.5%); Yemen (25.0%); Syria (8.1%); Other (2.5%).<br>Christians: Algeria (0%); Egypt (33.3%); Iraq (10.5%); Jordan (10.5%); Lebanon (19.3%); Morocco (3.5%); Palestine (5.3%); Yemen (0%); Syria (15.8%); Other (1.8%).<br>Muslims: Algeria (3.9%); Egypt (18.5%); Iraq (2.9%); Jordan (3.9%); Lebanon (3.9%); Morocco (12.6%); Palestine (8.7%); Yemen (38.8%); Syria (3.9%); Other (2.9%). | AGE: Overall Mean 46.1 years (SD=8.1); Christians Mean 47.3 years (SD=7.8); Muslims Mean 45.4 years (SD=8.3). | Female (100%) Note: Data on their children's gender was not collected. | Parent (migrant mother) | n/a | Self-report: Participating migrants' children vaccinated: Total participants of 30.8% (Religious affiliations: Christians 43.1% vs Muslims 23.8%). | High risk |
| [8], Adegboyega A, 2023 | Mixed methods (but used qualitative component only) | October 2020 - April 2021 (recruitment) | USA | Community | N=40 (38 only completed FG) | n=22 | 55.0% | Not specified | NATIVITY/ETHNICITY: African American (45.0%); Sub-Saharan African-born migrant (55.0%) | AGE: Mean 22.2 years (SD=4.5) | Both gender. Female (50.0%); Male (50.0%). | Recipient of vaccine (young adults) | n/a | Self-report: Vaccinated: 37.5% of total participants (Race/ethnicity - 22.7% of migrants [African/African immigrant 5/22] vs 55.6% of non-migrants [African American 10/18]). | Moderate risk |
| [9], Khan A,2023 | Mixed-methods ("Qualitative mixed methods") | August - September 2022; January - February 2023 | Canada | Community | N=31 (Parents (with children aged 9–18 years) | n=31 | 100.0% | FG participants: Immigrants (n=11); Refugees (n=5)<br><br>Very recent Immigrants | RACE/ETHNICITY of Semi-structured online interview & Short online survey participants (n=15): Black (n=6; 40.0%); Chinese (n=1; 6.7%); South Asian (e.g., East Indian, Pakistani, Sri Lankan, etc.) (n=6; 40.0%); Southeast Asian (e.g., Vietnamese, Cambodian, Laotian, Thai, etc.) (n=2; 13.3%). | AGE: No average age data.<br><br>Parents: aged 40–50 (n = 9); aged 30–40 (n = 3); aged 20-30 (n = 2); aged 50–60 (n = 2). | Interview participants (n=15): Female parents (n=10) and Male parents (n=5).<br><br>Gender of children of interview participants: Male child(ren) (n=2; 13.3%); | Parents.<br><br>FG participants: Mothers; Fathers; Guardian. | Publicly funded school-based vaccination, and catch-up vaccination at specialised immunis | Self-report: Respondent's HPV vaccine status: 26.7% (all were migrants) | Moderate risk |

| Study ID, First author, Publication year | Study type/ design | Year(s) of study | Location/country of study | Setting | Total number of participants/ sample size (study completers) | No. of immigrants/first-generation & descendants/ second-generation | Proportion of immigrants/first-generation & descendants/second-generation | Migratory status/ Reason for migration | Country/Region of origin/ Nativity/ Ethnicity(/race) | Age | Gender/ sex | Participant type | Intervention/ programme | Uptake of HPV vaccine | Study risk of bias scores |
|---|---|---|---|---|---|---|---|---|---|---|---|---|---|---|---|
| | | | | | | | | (landed immigrants who had been in Canada for five years or less) (n=9; 60.0%); Recent Immigrants (landed immigrants for more than 5 to 10 years in Canada) (n=3; 20.0%); Established Immigrants (landed immigrants for more than 10 years) (n=2; 13.3%); Refugee (landed as a refugee) (n=1; 6.7%). | RACE/ETHNICITY of FG & Detailed online survey participants: (n=16): Black immigrant parents (n = 6); South/SE Asian immigrant parents (n = 5); and West Asian refugee parents (n = 5). | | Female child(ren) (n=7; 46.7%); Both male and female children (n=6; 40.0%)<br><br>FG participants (n=16): Females (n=8); Males (n=8). Black (females n=0; males n=6); Asian (females n=4; males n=1); West Asian (females n=4; males n=1). | | ation clinics | | |
| [10], Kim SW, 2023 | Mixed methods (but largely | July 2021; December 2021; January 2022 | USA | Community | N=8 (First-generation immigrants) | n=8 (First-generation immigrant) | 100.0% | Not specified | COUNTRY OF ORIGIN: First generation: Vietnamese American (n=2) and Korean American mothers (n=6) | Age of adult women: Mean 41.4 years (SD 5.8 years).<br><br>Age of target child: Korean American: | Mothers: Female (100%).<br><br>Target child: Korean American: Male | Mothers or female primary caregivers | Virtual Digital Storytelling (DST) | n/a | Low risk |

| Study ID, First author, Publication year | Study type/ design | Year(s) of study | Location/country of study | Setting | Total number of participants/ sample size (study completers) | No. of immigrants/first-generation & descendants/ second-generation | Proportion of immigrants/first-generation & descendants/second-generation | Migratory status/ Reason for migration | Country/Region of origin/ Nativity/ Ethnicity(/race) | Age | Gender/ sex | Participant type | Intervention/ programme | Uptake of HPV vaccine | Study risk of bias scores |
|---|---|---|---|---|---|---|---|---|---|---|---|---|---|---|---|
| | qualitative) | | | | | | | | | Mean 12.6 years (SD 1.8); Vietnamese American: Mean 14.0 years (SD 2.4). | (n=3; 50%); Female (n=3; 50%); Vietnamese American: Male (n=0; 0%); Female (n=2; 100%). | | | | |
| [11], Mupandawana ET, 2016 | Qualitative | n/a | England | Community | N=10 (5 African couples who are parents of ≥1 daughter aged 8-14) | n=10 | 100.0% | Not specified | COUNTRY OF ORIGIN: Nigeria (20%); Kenya (20%); Zambia (20%); Zimbabwe (20%); South Africa (20%). | AGE (Mother): Mean 40.6 years (SD=2.4; range 38-44);<br>AGE (Father): No average age data available due to not all data available. | Both gender | Parents (5 couples of mother and father). Note: Kenyan mother is also a nurse. | School-based HPV vaccination (since 2008 for girls aged 12–13) | n/a | Low risk |
| [12], Ramírez M, 2014 | Qualitative | n/a | USA | Community | N=17 (mothers/ grandmothers of adolescent girls from diverse Hispanic backgrounds in a large north-eastern city in US) | n=6 | 35.3% | Not specified | NATIVITY: Foreign-born (35.3%); US territory-born (64.7%).<br>COUNTRY OF ORIGIN/ETHNICITY: Puerto Rican (64.7%); Dominican (11.8%); Mexican (5.9%); Mexican/ Costa Rican (11.8%); Puerto Rican/ Non-Hispanic White (5.9%). | AGE: Mean 30 years (range 26-76). | Female (100%, mothers and grandmothers of adolescent girls from diverse Hispanic background) | (Grand) parent (mother/ grandmother). Mother (88.2%) and grandmother (11.8%) of adolescent girls from diverse Hispanic backgrounds in a large north-eastern city in US. | Mandatory was mentioned, but not the main intervention; the main programme was routine HPV vaccination for girls aged 11-12 (recommended by Advisory Committee on Immunization Practice | n/a | Low risk |

| Study ID, First author, Publication year | Study type/ design | Year(s) of study | Location/country of study | Setting | Total number of participants/ sample size (study completers) | No. of immigrants/first-generation & descendants/ second-generation | Proportion of immigrants/first-generation & descendants/second-generation | Migratory status/ Reason for migration | Country/Region of origin/ Nativity/ Ethnicity(/race) | Age | Gender/ sex | Participant type | Intervention/ programme | Uptake of HPV vaccine | Study risk of bias scores |
|---|---|---|---|---|---|---|---|---|---|---|---|---|---|---|---|
| | | | | | | | | | | | | | s [ACIP]) | | |
| [13], Walter D, 2013 | Qualitative | March 2011 | Germany | Community/Clinic | N=72 (participants in Berlin and Heidelberg, Germany) | n=52 | 72.2% | Not specified | NATIVITY: a) Migrant mothers/ Adolescent with at least one-sided migration background (81.3%); b) German-born adolescents without migration background (18.7%); c) No information for 8 vaccinating doctors in private practice.<br><br>ETHNICITY/RACE: Turkish migrant mothers/adolescent with migration background (27.8%); Russian migrant mothers/adolescent migration background (27.8%); Arabic adolescent migration background (16.7%); German adolescents (16.7%). Note: No information for 8 vaccinating doctors (11.1%) in private practice. | Adolescent descendants, aged 14-16 (n= 48; 66.7%): 36 adolescents (50.0%) with migration background - descendants with at least one-sided migration background; 12 adolescents (16.7%) without migration background; Mothers of children with Turkish and Russian migration background (n=16): No age data available; Physicians in private practice - resident vaccinating doctors (in paediatrics, youth medicine, general medicine and Gynecology from Berlin districts with a high proportion of people with a migration background) (n= 8): No age data available. | Both gender (teenage descendants); Female (100%, mothers). GENDER: Male (24 adolescent boys, 33.3%); Female (24 adolescent girls + 16 mothers, 55.6%). No gender information on 8 vaccinating doctors (11.1%). | Recipient of vaccine; Parent (mother); Healthcare provider | n/a | n/a | Moderate risk |
| [14], Zeraiq L, 2015 | Qualitative | January 2011 and January 2012 (data collection) | Denmark | Community | N=36 (23 mothers and 13 daughters) | n=23 (mothers). n=13 (daughters) [all Palestinians born in Denmark] | 100% (mothers). 100% (daughters). | Not specified | COUNTRY OF ORIGIN/ETHNICITY (Mother): Lebanese (8.7%); Iraqi (4.3%); Palestinian, but born in Lebanon (87.0%).<br><br>COUNTRY OF ORIGIN/ETHNICITY (Daughter): Denmark-born Palestinians (100%). | AGE (Mother): no average age data available. AGE GROUP (Mother): aged 16-23 (26.1%); aged 17-23 (21.7%); aged 17-25 (17.4%); aged 17-26 (17.4%); aged 20-25 (17.4%). | Female (100%) | Parent (mother); Recipient of vaccine (daughter). | Free-of-charge for girls aged 12–18 (parents consent necessary) | n/a | Low risk |

| Study ID, First author, Publication year | Study type/ design | Year(s) of study | Location/country of study | Setting | Total number of participants/ sample size (study completers) | No. of immigrants/first-generation & descendants/ second-generation | Proportion of immigrants/first-generation & descendants/second-generation | Migratory status/ Reason for migration | Country/Region of origin/ Nativity/ Ethnicity(/race) | Age | Gender/ sex | Participant type | Intervention/ programme | Uptake of HPV vaccine | Study risk of bias scores |
|---|---|---|---|---|---|---|---|---|---|---|---|---|---|---|---|
| | | | | | | | | | | AGE (Daughter): no average age data available.<br>AGE GROUP (Daughter): aged 13-15 (30.8%), aged 15-17 (69.2%). | | | | | |
| [15], Dailey PM, 2017 | Qualitative | n/a | USA | Community | N=20 (19 Somali female immigrant parents and 1 female guardian) | n=20 | 100.0% | Not specified | COUNTRY OF ORIGIN: Somali (100%, the largest African immigrant population in the USA). Black/African Muslims (active in practice). | AGE (participant): Mean 36.4 years (SD=5.7);<br>AGE (participant's children): Mean 10.7 years (SD=1.5). | Female (100%) | Parent/guardian | n/a | n/a | Low risk |
| [16], Celentano I, 2021 | Qualitative | October 2017 - September 2018 | USA | Community | N=30 (East African mothers) | n=30 | 100.0% | Not specified | NATIVITY: Foreign-born (100%)<br>COUNTRY OF ORIGIN: Somalia, Ethiopia, Eritrea (no disaggregated data available). | AGE (Mother): Mean 41.0 years (SD=5.6). | Female (100%) | Parent (mother) | Comic book | Self-report: Migrants with ≥1 child aged 11–17 vaccinated: 20% | Low risk |
| [17], Scarinci IC, 2007 | Qualitative | April 2004 - March 2005 (data collection) | USA | Community | N=55 (participants with no personal history of cervical cancer, and sexually active in the last 6 months) | n=28 | 50.9% | Not specified | NATIVITY/ETHNICITY: Latina migrants (50.9 %); African Americans (49.1%). | AGE (Latina immigrant women): Mean 27.9 years (SD=6.19; range 17-36). | Female (100%) | POTENTIAL recipient of vaccine (older adolescents and young adults) in the context of pre-HPV vaccination programme in the USA. | n/a | n/a | Low risk |
| [18], Gao H, 2016 | Qualitative | October/November 2013 | USA | University | N=44 (Chinese international students, residing in Midwest of US) | n=44 | 100.0% | International students | COUNTRY OF ORIGIN: China (100%) | AGE: Mean 24.6 years (SD=3.49; range 18-34). | Both gender. Female (52.27%); Male (47.73%). | Recipient of vaccine | Informational pamphlet about Gardasil® | n/a | Low risk |

| Study ID, First author, Publication year | Study type/ design | Year(s) of study | Location/country of study | Setting | Total number of participants/ sample size (study completers) | No. of immigrants/first-generation & descendants/ second-generation | Proportion of immigrants/first-generation & descendants/second-generation | Migratory status/ Reason for migration | Country/Region of origin/ Nativity/ Ethnicity(/race) | Age | Gender/ sex | Participant type | Intervention/ programme | Uptake of HPV vaccine | Study risk of bias scores |
|---|---|---|---|---|---|---|---|---|---|---|---|---|---|---|---|
| [19], Luque JS, 2012 | Qualitative | n/a | USA | Clinic; Community | N=20 (5 Hispanic mothers + 7 Hispanic fathers + 8 healthcare providers of Vaccines for Children [VFC] programme, including nurse practitioners, nurse managers, registered nurses, physician assistants, and one physician) | n=12 | 60.0% | Not specified | COUNTRY OF ORIGIN (MOTHER): Mexico (100%); COUNTRY OF ORIGIN (FATHER): Mexico (86%); Honduras (14%). | AGE GROUP (Mother): aged 25-34 (n=3; 60%); aged 35-44 (n=2; 40%); AGE GROUP (Father): aged 25-34 (n=5; 71%); aged 35-44 (n=2; 29%); AGE GROUP (Healthcare providers): aged 25-34 (n=1; 12%); aged 45-54 (n=6; 75%); aged 55-64 (n=1; 12%). AGE (Vaccine eligible child, Hispanic adolescents): Mothers' child (Mean 12.7 years; range 10-18); Fathers' child (Mean 12.8 years; range 9-18) | Both gender | Healthcare provider; Parents (mothers and fathers). | Federally funded Vaccines for Children (VFC) programme (free vaccines for low-income children) | n/a | Low risk |
| [20], Rubens-Augustson T, 2019 | Qualitative | March-April 2018 | Canada | Community | N=10 (healthcare providers) | n=2 | 20.0% | Not specified | NATIVITY/GENERATIONAL STATUS: Newcomer Yes (10%); No (80%); Second generation (10%). | AGE: No average age data available. AGE GROUP: aged 18-25 (n=1; 10%); age 26-35 (n=2; 20%); age 36-45 (n=5; 50%); aged 46-55 (n=0; 0%); aged 56+ (n=2; 20%). | Both gender. Female (80%); Male (20%) | Healthcare provider | Publicly-funded school-based programme and catch-up clinics | n/a | Low risk |
| [21], Aragones A, 2016 | Qualitative | n/a | USA | Community | N=36 (Latino immigrant parents of minors [i.e., 9–17 year old] who had not yet initiated the HPV vaccine series) | n=36 | 100.0% | Not specified | COUNTRY OF ORIGIN: Colombia (16%); Dominican Republic (11%); Ecuador (30%); Mexico (25%); Peru (8%); Other (10%). | AGE: Mean 42 years (SD=10.43; range 25-65). | Both gender. Female (91%); Male (9%). | Parents (of minors, i.e., aged 9-17). | Self-paid (scenario) | n/a | Low risk |

| Study ID, First author, Publication year | Study type/ design | Year(s) of study | Location/country of study | Setting | Total number of participants/ sample size (study completers) | No. of immigrants/first-generation & descendants/ second-generation | Proportion of immigrants/first-generation & descendants/second-generation | Migratory status/ Reason for migration | Country/Region of origin/ Nativity/ Ethnicity(/race) | Age | Gender/ sex | Participant type | Intervention/ programme | Uptake of HPV vaccine | Study risk of bias scores |
|---|---|---|---|---|---|---|---|---|---|---|---|---|---|---|---|
| [22], Kim M, 2017 | Qualitative | June-July 2015 | USA | College | N=20 (Korean American female college students) | n=14 | 70% Notes: South Korea First-generation (foreign-born who arrived in the USA when aged ≥18) students (25%); South Korea 1.5 generation (foreign-born who arrived in the USA when aged <18) students (45%); US-born South Korea Second-generation students (30%). | First-/1.5/second-generation students | NATIVITY/COUNTRY OF ORIGIN: South Korea (70%); US-born (second generation, 30%). South Korea First-generation (25%); South Korea 1.5 generation (45%); South Korea Second-generation = US-born (30%) | AGE: Mean 21.7 years (SD=2.48). AGE GROUP WHEN MOVED TO US (N=14): Younger than 18 years (1.5 generation, n=9; 64%); 18 years and older (first generation, n=5; 36%). | Female (100%) | Recipient of vaccine | n/a | n/a | Low risk |
| [23], McComb E, 2018 | Qualitative | n/a | Canada | Community | N=11 (migrant women, aged 18-26) | n=11 | 100.0% | Refugees and immigrants (Students) | REGION OF ORIGIN: Africa (36%); Asia (45%); South America (18%). | AGE: Mean 23.5 years (SD=2.4). | Female (100%) | Recipient of vaccine | Catch-up HPV vaccination | n/a | Low risk |
| [24], Lindsay AC, 2021 | Qualitative | October 2019 - February 2020 | USA | Community | N=19 (fathers) | n=12 NB: The above foreign-born fathers have adolescent boys (~53%) and girls (~74%) aged 11-19. | 63.2% | Not specified | COUNTRY OF ORIGIN (FATHER): Puerto Rico (31.5%); Dominican Republic (26.3%); Colombia (21.1%); Peru (5.3%); El Salvador (5.3%); Guatemala (5.3%); USA (5.3%). REGION OF ORIGIN: Caribbean (57.9%); South America (~26%); Central America (~11%). | AGE: Median 49 years (range: 34–57). Note: Son's age: Mean 13.4 years (SD=2.1); Daughter's age: Mean 14.3 years (SD=2.7); Both gender of adolescents: Mean 13.8 years. | Male (100%) | Parent (Father) | n/a | n/a | Low risk |
| [25], Vamos CA, 2021 | Qualitative | 2015 | USA | Community/Clinic | N=13 (parents/caregivers with a daughter and/or son ages 9-15 from a rural, faith-based, community organisation in Florida) | n=13 | 100.0% | Labour migrant (migrant farmworkers) | COUNTRY OF ORIGIN: Mexico (100%). ETHNICITY: Hispanic (100%). RACE: White (30%); Other (62%); Don't know (8%) | AGE: Mean 36.23 years (SD=5.04) | Both gender. Female (92%, mother of at least 1 child aged 9-15); Male (8%, father of at least 1 child aged 9-15). | Parents/caregivers | Federally funded Vaccines for Children (VFC) Programme; | Self-report: Receipt of HPV vaccine in participating migrants' any children: 38% | Low risk |

| Study ID, First author, Publication year | Study type/ design | Year(s) of study | Location/country of study | Setting | Total number of participants/ sample size (study completers) | No. of immigrants/first-generation & descendants/ second-generation | Proportion of immigrants/first-generation & descendants/second-generation | Migratory status/ Reason for migration | Country/Region of origin/ Nativity/ Ethnicity(/race) | Age | Gender/ sex | Participant type | Intervention/ programme | Uptake of HPV vaccine | Study risk of bias scores |
|---|---|---|---|---|---|---|---|---|---|---|---|---|---|---|---|
| | | | | | | | | | | | | | Medicaid | | |
| [26], Jackson C, 2017 | Qualitative | December 2013 - April 2015 (Recruitment and data collection) | UK | Community | N=174 (participants from 6 Traveller communities based in 4 UK cities: Bristol; Glasgow; York; London) | n=97 | 55.7% | Not specified | NATIVITY/ ETHNICITY: Foreign-born (55.7%); UK-born (44.3%).<br><br>COUNTRY OF ORIGIN: FOREIGN-BORN: Bristol Romanian Roma (13.8%); Bristol Irish Traveller (5.2%); Glasgow Romanian Roma (9.8%); Glasgow Slovakian Roma (11.5%); London Irish Traveller (15.5%);<br>LOCALLY BORN: Bristol English Gypsy (8.6% - 1/15 is Welsh Gypsy); York English Gypsy (27.6%); Glasgow Scottish Showpeople (8.0%).<br>Note: 6 Traveller communities based in 4 UK cities (Bristol; Glasgow; York; London). The English Gypsy, European Roma and Irish Traveller communities are recognised legally as ethnic minorities.<br>Note: Roma and Traveller are those living on an authorised caravan/trailer/chalet site or housed, excluding those living on the roadside /unauthorised encampments. | n/a | Both gender. Female (79.9%); Male (20.1%).<br><br>Bristol Romanian Roma (Female 58.3 %; Male 41.7%); Bristol Irish Traveller (Female 77.8%; Male 22.2%); Glasgow Romanian Roma (Female %; Male %); Glasgow Slovakian Roma (Female 85.0%; Male 15.0%); London Irish Traveller (Female 100.0%; Male 0.0%);<br><br>Bristol English Gypsy (Female 66.7%; Male 33.3%; 1/15 is Welsh Gypsy); York English Gypsy (Female 77.1%; Male 22.9%); Glasgow Scottish Showpeople (Female 71.4%; Male 28.6%). | (Grand) Parents (Mother; Grandmother; Father; Grandfather); [Pregnant women]; Female no children; Male no children; Recipient of vaccine (adolescent girls eligible for HPV vaccine, aged 12-13). Note: Adolescent boys were not included. | School-based HPV vaccination programme (e.g., girls aged 12-13 given at school) | n/a | Low risk |

| Study ID, First author, Publication year | Study type/ design | Year(s) of study | Location/country of study | Setting | Total number of participants/ sample size (study completers) | No. of immigrants/first-generation & descendants/ second-generation | Proportion of immigrants/first-generation & descendants/second-generation | Migratory status/ Reason for migration | Country/Region of origin/ Nativity/ Ethnicity(/race) | Age | Gender/ sex | Participant type | Intervention/ programme | Uptake of HPV vaccine | Study risk of bias scores |
|---|---|---|---|---|---|---|---|---|---|---|---|---|---|---|---|
| [27], Kobetz E, 2011 | Qualitative | 2010 | USA | Community | N=41 (Haitian immigrant women) | n=41 | 100.0% | Not specified | COUNTRY OF ORIGIN: Haiti (100%) | AGE: No average age data available. Range 21-71. AGE GROUP: aged 21-30 (n=9; 22.0%); aged 31-40 (n=11; 26.8%); aged 41-50 (n=7; 17.1%); aged Over 50 (n=14; 34.1%). | Female (100%) | Recipient of vaccine (12.2%); Parents (~54%). | n/a | n/a | Low risk |
| [28], Burke NJ, 2015 | Qualitative | n/a | USA | Community | N=25 (Cambodian Khmer mothers with at least one daughter aged 9-17 living in the Seattle–Tacoma Metropolitan Area) | n=25 | 100.0% | Refugee | COUNTRY OF ORIGIN: Cambodia (100%). | AGE: No average age data available. AGE GROUP: aged 30-39 (n=4; 16%); aged 40-49 (n=16; 60%); aged ≥50 (n=6; 24%). | Female (100%) | Parent (mother) | n/a | Self-report: Receipt of HPV vaccine in participating migrants' daughters: 36% | Low risk |
| [29], Javanbakht M, 2012 | Qualitative | March - May 2009 | USA | Clinic | N=21 (providers/medical staff, providing care to adolescent girls [aged 11–17] at two clinics in Los Angeles) | n/a ("a large immigrant population [predominantly Hispanic] serviced by the clinic site") | n/a ("a large immigrant population [predominantly Hispanic] serviced by the clinic site") | Not specified | ETHNICITY: Hispanic (71.4%); Non-Hispanic (28.6%). | n/a | Both gender. Female (81%); Male (19%). | Healthcare provider | Vaccines for Children (no cost vaccine programme) | n/a | Low risk |
| [30], Perkins RB, 2013 | Qualitative | August 2009 - January 2011 (interview data collection) | USA | Clinic (community health centres) | N=34 (providers from 4 federally qualified community health centre, serving Boston's low-income and minority populations: 25 doctors [paediatric and family medicine physicians] + 9 nurse | n/a | n/a | Not specified | RACE/ETHNICITY: White (76.5%); Latino (8.8%); Asian (8.8%); Indian (5.9%). | n/a | Both gender. Physicians: Female (76%); Male (24%); Nurse practitioners: Female (100%); Male (0%). | Healthcare provider | n/a | n/a | Low risk |

| Study ID, First author, Publication year | Study type/ design | Year(s) of study | Location/country of study | Setting | Total number of participants/ sample size (study completers) | No. of immigrants/first-generation & descendants/ second-generation | Proportion of immigrants/first-generation & descendants/second-generation | Migratory status/ Reason for migration | Country/Region of origin/ Nativity/ Ethnicity(/race) | Age | Gender/ sex | Participant type | Intervention/programme | Uptake of HPV vaccine | Study risk of bias scores |
|---|---|---|---|---|---|---|---|---|---|---|---|---|---|---|---|
| | | | | | practitioners in primary care) | | | | | | | | | | |
| [31], Gorman DR, 2019 | Qualitative | March 2018 (Focus group) | Scotland | Community | N=13 (Polish migrant women, representing 11 families) | n=13 | 100.0% | Not specified | COUNTRY OF ORIGIN: Poland (100%). | AGE GROUP: aged 18-29 (n=1; 7.7%); aged 30-44 (n=10; 76.9%); aged 45-59 (n=0; 0%); aged 60+ (n=2; 15.4%). | Female (100%). | (Grand) Parents | School-based (free of charge) | n/a | Low risk |
| [32], Vamos CA, 2022 | Qualitative | n/a | USA | Multi-level (System level) | N=13 (diverse stakeholders representative of health, social services, and political sectors [across the organisational, community and the societal levels]) | n/a | n/a | Migrant farmworker | ETHNICITY (USERS): Latinx (100%). | n/a | n/a | Stakeholders (diverse stakeholders representing health/ social services/ political sectors at multi-level). | Federally funded Vaccines for Children (VFC) Programme; Medicaid | n/a | Low risk |
| [33], Ganczak M, 2021 | Qualitative | September 2019 | Poland | Community (interviews done at a medical university) | N=22 (Ukrainian migrants, aged 18-45) | n=22 | 100.0% | Economic migrant | COUNTRY OF ORIGIN: Ukraine (100%). | AGE GROUP: aged 18-20 (n=2; 9.1%); aged 21-30 (n=11; 50.0%); aged 31-40 (n=8; 36.4%); aged >40 (n=1; 4.5%). NB. Inconsistent data: "one was 18 years, eleven were in their 20s, nine in their 30s, one in his 40s". | Both gender. Female (45.5%); Male (54.5%). | Recipient of vaccine; Parents | HPV vaccination recommended in the Polish immunization programme (non-mandatory) and self-paid | n/a | Low risk |
| [34], Pratt R, 2019 | Qualitative | n/a | USA | Clinic (primary care); High school | N=34 (22 young adult women; 12 young adult men) | n=27 | 79.4% | Not specified | NATIVITY: Foreign-born (79.4%); US-born (20.6%). ETHNICITY: African American/Black (8.8%); Somali (88.2%); Other (2.9% - Oromo, an ethnic group from Ethiopia). | AGE: Mean 19.9 years (SD=1.46) | Both gender. Female (64.7%); Male (35.3%). | Recipient of vaccine (young adults targeting catch-up HPV vaccination) | n/a | n/a | Low risk |

| Study ID, First author, Publication year | Study type/ design | Year(s) of study | Location/country of study | Setting | Total number of participants/ sample size (study completers) | No. of immigrants/first-generation & descendants/ second-generation | Proportion of immigrants/first-generation & descendants/second-generation | Migratory status/ Reason for migration | Country/Region of origin/ Nativity/ Ethnicity(/race) | Age | Gender/ sex | Participant type | Intervention/ programme | Uptake of HPV vaccine | Study risk of bias scores |
|---|---|---|---|---|---|---|---|---|---|---|---|---|---|---|---|
| [35], Ko LK, 2019 | Qualitative | February–April 2017 | USA | Community | N=30 (East African migrant mothers [incl. Somali] of children aged 11-17) | n=30 | 100.0% | Not specified | COUNTRY OF ORIGIN (MOTHER) within each spoken language: Somali (Somali 90.0%; Kenya 9.1%); Amharic (Ethiopia 90.0%; Eritrea 10.0%); Tigrinya (Ethiopia 22.2%; Eritrea 77.8%).<br><br>ETHNICITY (MOTHER) within each spoken language: Somali (Somali 100%); Amharic (Amhara 60%; Hadere 10%; Tigre 20%; Oromo 10%); Tigrinya (Tigre 100%).<br><br>ETHNICITY (MOTHER): Mothers of 11–17 year old children from Somali (36.7%); Amharic (33.3%); Tigrinya (30.0%). | AGE (Mother): Somali (Mean 42.9 years; SD=6.0); Amharic (Mean 35.9 years; SD=1.8); Tigrinya (Mean 44.4 years; SD=3.5). AGE (Children aged 11-17): Somali (Mean 14.8 years; SD=1.9); Amharic (Mean 12.7 years; SD=4.2); Tigrinya (Mean 15 years; SD=5.2). | Mother: Female (100%).<br><br>11-17 year old children: Somali (Female only 18.2%; Male only 54.6%; Male & female 27.3%); Amharic (Female only 60.0%; Male only 40.0%; Male & female 0%); Tigrinya (Female only 66.7%; Male only 22.2%; Male & female 11.1%). | Parent (mother) | Part of the development of a multi-level communication intervention to promote HPV vaccination in Somali, Ethiopian, and Eritrean communities (e.g., comic books for adolescents, educational forums for mothers, an online continuing education course for health providers) | Self-report: ≥1 child HPV vaccinated: 20.5% of total migrants (Somali 18.2% vs Amharic 10.0% vs Tigrinya 33.3%). | Low risk |
| [36], Ganczak M, 2022 | Qualitative | December 2021–March 2022 | Poland | Community; Clinic (primary care clinics and | N=58 (n=46 Ukrainian migrants aged 15–45 [34 adults including 4 grandmothers, | n=46 | 79.3% | (Recent migrant. Note: A recent migrant in this study | COUNTRY OF ORIGIN (Migrants): Ukraine (100%); COUNTRY OF ORIGIN (Healthcare workers): Poland, Ukraine, etc (not full details provided). | AGE (Ukrainian migrants, n=46): Mean 32.1 years; AGE (Healthcare workers, n=12): Mean 39.6 years. | Both gender. Ukrainian migrants: Female (97.8%). Healthcare workers: | (Grand) Parents (migrant adult); Recipient of vaccine (adolescen | HPV vaccination recommended in the Polish | n/a | Low risk |

| Study ID, First author, Publication year | Study type/ design | Year(s) of study | Location/country of study | Setting | Total number of participants/ sample size (study completers) | No. of immigrants/first-generation & descendants/ second-generation | Proportion of immigrants/first-generation & descendants/second-generation | Migratory status/ Reason for migration | Country/Region of origin/ Nativity/ Ethnicity(/race) | Age | Gender/ sex | Participant type | Intervention/ programme | Uptake of HPV vaccine | Study risk of bias scores |
|---|---|---|---|---|---|---|---|---|---|---|---|---|---|---|---|
| | | | | immunisation centres) | plus 12 teenage girls]; and n=12 healthcare workers) | | | refers to "resident in Poland for a minimum of 6 months and a maximum of 10 years"). | | Note: "In Poland, participants 12–16 years of age are able to sign a co-consent". | Female (66.7%). | t girls); Healthcare provider. | immunization programme (non-mandatory) and self-paid | | |
| [37], Garcia S, 2022 | Qualitative | March - August 2021 (recruitment) | USA | Clinic (Federally Qualified Health Centres [FQHCs]) | N=30 (Unvaccinated Mexican-born: n=15 Unvaccinated US-born Mexican American: n=15) | n=15 | 50.0% | Not specified | NATIVITY/COUNTRY OF ORIGIN/ETHNICITY: Mexico-born (50%); US-born Mexican American (50%). | AGE: All Mexican American: Mean 23 years (SD=1.77); Unvaccinated Mexico-born: Mean 23 years (SD=2.0); Unvaccinated US-born: Mean 24 years (SD=1.5). | Female (100%) | Vaccine eligible women (young mothers 66.7%; young adults 33.3% - an equal distribution between Mexico-born and US-born Mexican American) | n/a | n/a | Low risk |
| [38], Netfa F, 2023 | Qualitative | April 2021 - July 2021 | Australia | Community | N=16 (16 Arabic-speaking mothers: face-to-face interviews: n=13; videoconferencing due to COVID-19 public health restrictions: n=3) | n=16 | 100.0% | Not specified | COUNTRY OF ORIGIN: Libyan (n=8/16; 50.0%); Sudanese (n=1/16; 6.3%); Moroccan (n=1/16; 6.3%); Palestinian (n=2/16; 12.5%); Kuwaiti (n=1/16; 6.3%); Lebanese (n=1/16; 6.3%); Egyptian (n=1/16; 6.3%); Syrian (n=1/16; 6.3%). | Age of mothers: Mean 42.3 years (SD = 4.6; range = 32-50 years). | Mothers' gender/sex: Female (100%).<br><br>Child's gender/sex: Female (n=11/18; 61.1 %, girls); Male (n=7/18; 38.9 %, boys). | Mothers | Australian school vaccination programme | Self-report: Receipt of HPV vaccine in migrant mothers' their child): 87.5% | Low risk |
| [39], Chadenie | Quantitative, cross- | December 2008 - December | Italy | Clinic | N=516 (n=475 Mothers: Italy native [90.7%]; | n=44 | 9.3% | Not specified | NATIVITY (mothers only, excluding healthcare | AGE (Mother only, excluding healthcare professionals): Mean | Mothers: Female (100%). Health professionals: | Parent (mother); | First vaccination campaig | n/a | Moderate risk |

| Study ID, First author, Publication year | Study type/ design | Year(s) of study | Location/country of study | Setting | Total number of participants/ sample size (study completers) | No. of immigrants/first-generation & descendants/ second-generation | Proportion of immigrants/first-generation & descendants/second-generation | Migratory status/ Reason for migration | Country/Region of origin/ Nativity/ Ethnicity(/race) | Age | Gender/ sex | Participant type | Intervention/ programme | Uptake of HPV vaccine | Study risk of bias scores |
|---|---|---|---|---|---|---|---|---|---|---|---|---|---|---|---|
| r GMC, 2011 | sectional | 2009 (data collection). | | | Foreign-born [9.3%]; n=41 Healthcare professionals: Medical doctor [17.1%]; Nurse [68.3%]; Midwife [14.6%]). | | | | professionals): Foreign-born (9.3%); Italy-born (90.7%). | 42.0 years (SD=4.58) | gender data not available. | Healthcare provider | n in 2 suburbs of Milan | | |
| [40], Lu P-J, 2014 | Quantitative, cross-sectional | 2012 (data collection) ; 2013 (data analysis) | USA | Household | N=34,525 (all adults) | n=6,559 | 19.0% | Not specified | NATIVITY: Foreign-born (17.4%); US-born (82.6%).<br><br>REGION/COUNTRY OF ORIGIN:<br>Total: US (82.6%); Mexico/Central America/Caribbean Islands (8.6%); South America (1.0%); Europe (2.1%); Asia (4.0%); Other (1.7%).<br>US-born: US (100.0%).<br>Foreign-born: Mexico/Central America/Caribbean Islands (49.3%); South America (6.0%); Europe (12.3%); Asia (22.7%); Other (9.7%).<br><br>RACE/ETHNICITY:<br>Total: Non-Hispanic white (66.5%); Non-Hispanic black (11.5%); Hispanic (14.9%); Non-Hispanic Other (7.2%).<br>US-born: Non-Hispanic white (76.5%); Non-Hispanic black (12.4%); Hispanic (7.4%); Non-Hispanic Other (3.8%).<br>Foreign-born: Non-Hispanic white (18.9%); Non-Hispanic black (7.3%); Hispanic (50.5%); Non-Hispanic Other (23.3%). | AGE: No average age data available.<br><br>AGE GROUP (All): aged 18-49 (n=18,165; 56.4%); aged 50-64 (n=8,978; 25.8%); aged 65+ (n=7,382; 17.8%);<br>AGE GROUP (US-born): aged 18-49 (n=14,057; 54.7%); aged 50-64 (n=7,557; 26.5%); aged 65+ (n=6,342; 18.9%);<br>AGE GROUP (Foreign-born): aged 18-49 (n=4,102; 64.6%)*[p<0.05]; aged 50-64 (n=1,420; 22.7%); aged 65+ (n=1,037; 12.7%).<br>*p<0.05 by chi-square test (comparing US-born and foreign-born). | Both gender.<br>All: Female (51.9%); Male (48.1%).<br>US-born: Female (52.0%); Male (48.0%).<br>Foreign-born: Female (51.2%); Male (48.8%). | Recipient of vaccine | Routinely-recommended vaccinations for adults | Self-report: HPV vaccination (≥1 dose):<br>Male aged 18-26, overall 3.7 [95% CI: 2.7-5.1]; Nativity - US-born 4.2% [3.0-5.8] vs Foreign-born n/a (estimates not reliable); Duration of residence - Foreign-born living in US <10 years n/a (estimates not reliable) vs living in US ≥10 years n/a (estimates not reliable); Citizenship - Foreign-born US citizen n/a (estimates not reliable) vs Non-US citizen n/a (estimates not reliable).<br>**Female aged 18-26,** overall 35.6 [95% CI: 33.0-38.3]; Nativity - US-born 38.7% [35.9-41.6] vs Foreign-born 14.7% [10.9-19.6], p<0.05; Duration of | Moderate risk |

| Study ID, First author, Publication year | Study type/ design | Year(s) of study | Location/country of study | Setting | Total number of participants/ sample size (study completers) | No. of immigrants/first-generation & descendants/ second-generation | Proportion of immigrants/first-generation & descendants/second-generation | Migratory status/ Reason for migration | Country/Region of origin/ Nativity/ Ethnicity(/race) | Age | Gender/ sex | Participant type | Intervention/ programme | Uptake of HPV vaccine | Study risk of bias scores |
|---|---|---|---|---|---|---|---|---|---|---|---|---|---|---|---|
| | | | | | | | | | | | | | | residence - Foreign-born living in US <10 years 10.7% [6.7-16.7] vs living in US ≥10 years 19.1% [12.8-27.6]; Citizenship - Foreign-born US citizen 21.2% [13.6-31.5] vs Non-US citizen 10.4% [6.7-15.8], $p<0.05$.<br>HPV vaccination (≥1 dose): Male aged 18-26, US-born, English-speaking 4.0% [95% CI: 2.8-5.6] vs Non-English speaking n/a (estimates not reliable); Foreign-born, English-speaking n/a (estimates not reliable) vs Non-English speaking n/a (estimates not reliable). Female aged 18-26, US-born, English-speaking 39.1% [95% CI: 36.2-42.0] vs Non-English speaking 24.0% [14.1-37.7], $p<0.05$; Foreign-born, English-speaking 17.3% [12.1-24.0] vs Non-English speaking n/a (estimates not reliable).<br>HPV vaccination (≥1 dose): Race-ethnicity - Male | |

| Study ID, First author, Publication year | Study type/ design | Year(s) of study | Location/country of study | Setting | Total number of participants/ sample size (study completers) | No. of immigrants/first-generation & descendants/ second-generation | Proportion of immigrants/first-generation & descendants/second-generation | Migratory status/ Reason for migration | Country/Region of origin/ Nativity/ Ethnicity(/race) | Age | Gender/ sex | Participant type | Intervention/ programme | Uptake of HPV vaccine | Study risk of bias scores |
|---|---|---|---|---|---|---|---|---|---|---|---|---|---|---|---|
| | | | | | | | | | | | | | | aged 18-26, US-born, Non-Hispanic White 3.7% [95% CI: 2.4-5.6] vs Non-Hispanic Black n/a (estimates not reliable) vs Hispanic n/a (estimates not reliable) vs Other n/a (estimates not reliable); Foreign-born, Non-Hispanic White n/a (estimates not reliable) vs Non-Hispanic Black n/a (estimates not reliable) vs Hispanic n/a (estimates not reliable) vs Other n/a (estimates not reliable). Female aged 18-26, US-born, Non-Hispanic White 44.1% [95% CI: 40.5-47.8] vs Non-Hispanic Black 29.0% [23.1-35.7], p<0.05 vs Hispanic 25.6% [20.6-31.3], p<0.05 vs Other 38.6% (28.3-50.1); Foreign-born, Non-Hispanic White 25.7% (14.3-41.8) vs Non-Hispanic Black n/a (estimates not reliable) vs Hispanic 11.5% (7.1-18.1) vs Other n/a | |

| Study ID, First author, Publication year | Study type/ design | Year(s) of study | Location/country of study | Setting | Total number of participants/ sample size (study completers) | No. of immigrants/first-generation & descendants/ second-generation | Proportion of immigrants/first-generation & descendants/second-generation | Migratory status/ Reason for migration | Country/Region of origin/ Nativity/ Ethnicity(/race) | Age | Gender/ sex | Participant type | Intervention/ programme | Uptake of HPV vaccine | Study risk of bias scores |
|---|---|---|---|---|---|---|---|---|---|---|---|---|---|---|---|
| | | | | | | | | | | | | | | (estimates not reliable). Note: p < 0.05 by t-test (comparing race/ethnicity where non-Hispanic white is the reference group).<br>HPV vaccination (≥1 dose): Country/ region of origin – Male aged 18-26, US 4.2% [95% CI: 3.0-5.8] vs Mexico/ Central America/ Caribbean Islands n/a (estimates not reliable) vs South America n/a (estimates not reliable) vs Europe n/a (estimates not reliable) vs Asia n/a (estimates not reliable) vs Others n/a (estimates not reliable). Female aged 18-26, US 38.7% [95% CI: 35.9-41.6] vs Mexico/ Central America/ Caribbean Islands 8.8% [5.2-14.4], p<0.05 vs South America n/a (estimates not reliable) vs Europe 27.9% [15.0-46.0] vs Asia n/a (estimates not reliable) vs Others n/a (estimates not | |

| Study ID, First author, Publication year | Study type/ design | Year(s) of study | Location/country of study | Setting | Total number of participants/ sample size (study completers) | No. of immigrants/first-generation & descendants/ second-generation | Proportion of immigrants/first-generation & descendants/second-generation | Migratory status/ Reason for migration | Country/Region of origin/ Nativity/ Ethnicity(/race) | Age | Gender/ sex | Participant type | Intervention/ programme | Uptake of HPV vaccine | Study risk of bias scores |
|---|---|---|---|---|---|---|---|---|---|---|---|---|---|---|---|
| | | | | | | | | | | | | | | reliable). Note: < 0.05 by t-test (comparing birth country/region where United States is the reference group). HPV vaccination (≥1 dose): Adjusted adult vaccination coverage by Nativity - Male aged 18-26, US-born, Adjusted prevalence ratio [reference] vs US-born, Adjusted vaccination coverage n/a (not enough sample size); Foreign-born, Adjusted prevalence ratio n/a (not enough sample size); Foreign-born, Adjusted vaccination coverage n/a (not enough sample size). Female aged 18-26, US-born, Adjusted prevalence ratio [reference] vs US-born, Adjusted vaccination coverage 37.4% [95% CI: 34.5-40.4]; Foreign-born, Adjusted prevalence ratio 0.6% [0.4-0.9], significant; Foreign-born, Adjusted vaccination | |

| Study ID, First author, Publication year | Study type/ design | Year(s) of study | Location/country of study | Setting | Total number of participants/ sample size (study completers) | No. of immigrants/first-generation & descendants/ second-generation | Proportion of immigrants/first-generation & descendants/second-generation | Migratory status/ Reason for migration | Country/Region of origin/ Nativity/ Ethnicity(/race) | Age | Gender/ sex | Participant type | Intervention/ programme | Uptake of HPV vaccine | Study risk of bias scores |
|---|---|---|---|---|---|---|---|---|---|---|---|---|---|---|---|
| | | | | | | | | | | | | | | coverage 23.0% [16.2-31.5]. | |
| [41], Furgurson KF, 2019 | Quantitative, cross-sectional | June-August 2015 | USA | Camps | N=200 (participating adult Latina/o [Mexico-born] farmworkers [n=100; 50%]; participating adult Latina/o [Mexico-born] non-farmworkers [n=100; 50%]); (n=135 adolescent child, aged 11-17: having Farmworker parent [n=76; 56%]; having Non-farmworker parent [n=59; 44%]). | Adult Latina/o: n=200.<br><br>(Adolescent child, aged 11-17: n=73) | Adult Latina/o: 100%.<br><br>(Adolescent child, aged 11-17: 54.1%). | Labour migrant (farmworker); migrant (non-farmworker). | NATIVITY/COUNTRY OF ORIGIN/ETHNICITY (Adult participant): Mexico-born (100%) COUNTRY OF RESIDENCE (Adolescent child, aged 11-17): US 54.1%; Mexico 45.9%. | AGE (Adult participants): Farmworkers: Mean 37.6 years (SD=10.1); non-farmworkers Mean 41.1 years (SD=11.7) [p = 0.052].<br><br>(AGE GROUP (Adolescent child): Farmworkers: aged 11-12 (n=24; 32%); aged 13-14 (n=19; 25%); aged 15-17 (n=33; 43%); Non-farmworkers: aged 11-12 (n=20; 34%); aged 13-14 (n=16; 27%); aged 15-17 (n=23; 39%). [p=0.654] ) | Both gender. Farmworkers (Adult participant): Female (20%); Male (80%). Non-farmworkers (Adult participant): Female (50%); Male (50%). [p<0.001].<br><br>(Farmworkers (Adolescent child): Female (59%); Male (41%). Non-farmworkers (Adolescent child): Female (41%); Male (59%). [p=0.031]) | Parent (Adult farmworker/non-farmworker participant);<br><br>Recipient of vaccine (Participant's adolescent child, aged 11-17) | Mexico: School-based HPV vaccination programme for all girls in the fifth grade (2011) | Self-report: Initiation of HPV vaccine (≥1 dose) of participating parents' adolescent child (daughter/son): Overall 24.4% of total participants. Gender – Female, overall 28% (farmworker parent 24% vs Non-farmworker parent 33%, p=0.246); Male, overall 21% (farmworker parent 26% vs Non-farmworker parent 17%, p=0.267). Farmworker parent, children vaccinated 25% vs Non-farmworker parent, children vaccinated 24%. Country of residence – US, overall 34% (Farmworker parent 65% vs Non-farmworker parent 25%, p=0.038); Mexico, overall 8% (Farmworker parent 8% vs Non-farmworker parent 0%, p value n/a). Age – Aged 11-12, overall 23% (Farmworker parent 21% vs Non-farmworker | Moderate risk |

| Study ID, First author, Publication year | Study type/ design | Year(s) of study | Location/country of study | Setting | Total number of participants/ sample size (study completers) | No. of immigrants/first-generation & descendants/ second-generation | Proportion of immigrants/first-generation & descendants/second-generation | Migratory status/ Reason for migration | Country/Region of origin/ Nativity/ Ethnicity(/race) | Age | Gender/ sex | Participant type | Intervention/ programme | Uptake of HPV vaccine | Study risk of bias scores |
|---|---|---|---|---|---|---|---|---|---|---|---|---|---|---|---|
| | | | | | | | | | | | | | | parent 25%, p=0.743); Aged 13-14, overall 17% (Farmworker parent 26% vs Non-farmworker parent 6%, p=0.242); Aged 15-17, overall 30% (Farmworker parent 27% vs Non-farmworker parent 35%, p=0.328). Parent reporting vaccination status – Reported by Mother, overall 44% (Farmworker parent 65% vs Non-farmworker parent 35%, p=0.296); Reported by Father, overall 11% (Farmworker parent 14% vs Non-farmworker parent 5%, p=0.189. | |
| [42], Gerend MA, 2013 | Quantitative, cross-sectional | June - July 2010 (data collection) | USA | Clinic (Federal Qualified Health Clinic [FQHC]) | N=200 (Latina mothers including those who have more than one daughter aged 9-18, n=65) | n=154 | 77.0% | Not specified | NATIVITY: Foreign-born (77%); US-born (23%).<br><br>COUNTRY OF ORIGIN (among Foreign-born): Cuba (3%); El Salvador (1%); Guatemala (14%); Honduras (4%); Mexico (74%); Nicaragua (2%); Peru (<1%); Puerto Rico (<1%).<br>NB: Puerto Rico is categorised as foreign-born, but not US-born. | AGE (Mother, n=199): Mean 36.2 years (SD=7.3; range 23-61);<br>AGE (Their daughter, n=200): Mean 13 years (SD=3; range 9-18). | Female (100%, mothers) | Parent (mother) | Routine HPV vaccination for girls aged 11 (9)-12 and Catch-up vaccination for females aged 13-26 | n/a | Low risk |

| Study ID, First author, Publication year | Study type/ design | Year(s) of study | Location/country of study | Setting | Total number of participants/ sample size (study completers) | No. of immigrants/first-generation & descendants/ second-generation | Proportion of immigrants/first-generation & descendants/second-generation | Migratory status/ Reason for migration | Country/Region of origin/ Nativity/ Ethnicity(/race) | Age | Gender/ sex | Participant type | Intervention/ programme | Uptake of HPV vaccine | Study risk of bias scores |
|---|---|---|---|---|---|---|---|---|---|---|---|---|---|---|---|
| [43], Kepka D, 2015 | Quantitative, cross-sectional | August - October 2013 | USA | Community | N=118 (parents, representing 108 daughters and 92 sons, aged 11–17) | n=118 | 100.0% | Not specified | COUNTRY OF ORIGIN/BIRTHPLACE: Mexico (69.2%); Other (30.8%). ANCESTRY: Mexico (72.4%); Other (27.6%). | AGE GROUP: aged 18-39 (n=41; 39.4%); aged 40-49 (n=49; 47.1%); aged ≥50 (n=14; 13.5%). | Both gender of parents with both gender of adolescent children. Female (84.4%); Male (15.7%). | Parents (of HPV vaccine age-eligible adolescents) | Routine vaccination for girls aged 11–12 and Catch-up vaccination for female aged 13–26 (2006). Routine vaccination for boys aged 11–12 and Catch-up vaccination for male aged 13–21 (2009) | Self-report: Receipt of HPV vaccine (≥1 dose) among Participating' daughter(s)/ son(s): Daughters, overall 42.6% (46/108) vs Sons, overall 20.7% (19/92). Completion of HPV vaccine (3 doses): Daughters, overall 17.6% (19/108); Sons, overall 8.7% (8/92). Parents Age – aged 18-39, overall 39.4% vs aged 40-49, overall 47.1% vs aged ≥50, overall 13.5% Aged 18-39, daughters received 42.1% vs aged 40-49 40.4% vs aged ≥50 50.0%, p=0.482. Aged 18-39, sons received 12.5% vs aged 40-49 25.7% vs aged ≥50 27.3%, p=0.531. Parents Gender – Male, overall 15.7% vs Female, overall 84.4%; Male, daughters received 31.3% vs Female 42.7%, p=0.346; Male, sons received 3.8% vs Female 18.4%, p=0.604. Parents Marital status – Married, | High risk |

| Study ID, First author, Publication year | Study type/ design | Year(s) of study | Location/country of study | Setting | Total number of participants/ sample size (study completers) | No. of immigrants/first-generation & descendants/ second-generation | Proportion of immigrants/first-generation & descendants/second-generation | Migratory status/ Reason for migration | Country/Region of origin/ Nativity/ Ethnicity(/race) | Age | Gender/ sex | Participant type | Intervention/ programme | Uptake of HPV vaccine | Study risk of bias scores |
|---|---|---|---|---|---|---|---|---|---|---|---|---|---|---|---|
| | | | | | | | | | | | | | | overall 21.7% vs Other, overall 78.3%; Married, daughters received 39.0% vs Other 52.2%, p=0.521; Married, sons received 21.9% vs Other 17.7%, p=0.379.<br>Parents Education – <High school, overall 39.6% vs ≥High school, overall 60.4%; <High school, daughters received 38.5% vs ≥High school 41.4%, p=0.950; <High school, sons received 16.2% vs ≥High school 22.9%, p=0.872.<br>Parents Household income – <$20K, overall 47.3% vs $20K-$35K, overall 30.4% vs >$35K, overall 22.3%; <$20K, daughters received 48.9% vs $20K-$35K 40.0% vs >$35K 44.0%, p=0.961; <$20K, sons received 19.1% vs $20K-$35K 20.8% vs >$35K 30.0%, p=0.784.<br>Parents Country of origin – Mexico, overall 69.2% vs Other, overall 30.8%; Mexico, daughters received 37.3% vs Other 53.1%, p=0.288; | |

| Study ID, First author, Publication year | Study type/ design | Year(s) of study | Location/country of study | Setting | Total number of participants/ sample size (study completers) | No. of immigrants/first-generation & descendants/ second-generation | Proportion of immigrants/first-generation & descendants/second-generation | Migratory status/ Reason for migration | Country/Region of origin/ Nativity/ Ethnicity(/race) | Age | Gender/ sex | Participant type | Intervention/ programme | Uptake of HPV vaccine | Study risk of bias scores |
|---|---|---|---|---|---|---|---|---|---|---|---|---|---|---|---|
| | | | | | | | | | | | | | | Mexico, sons received 20.9% vs Other 20.8%, p=0.049. Parents duration of year in the USA – 0-14 years, overall 44.2% vs ≥15 years, overall 55.8%; 0-14 years, daughters received 32.5% vs ≥15 years 43.6%, p=0.035; 0-14 years, sons received 14.3% vs ≥15 years 23.9%, p=0.484. Parents Ancestry – Mexico, overall 72.4% vs Other, overall 27.6%; Mexico, daughters received 39.7% vs Other 46.4%, p=0.784; Mexico, sons received 21.4% vs Other 15.0%, p=0.165. Parents Acculturation – Acculturated, overall 50.0% vs Not acculturated, overall 50.0%; Acculturated, daughters received 42.1% vs Not acculturated 43.1%, p=0.274; Acculturated, sons received 16.7% vs Not acculturated 24.0%, p=0.621. | |
| [44], Kepka DL, 2012 | Quantitative, cross- | July–September | USA | Community | N=78 (rural Hispanic mothers/guardia | N=53 (Foreign-born | 67.9% (Foreign-born participating mother) | Not specified | NATIVITY/COUNTRY OF ORIGIN (Mother): Mexico-born (69.7%); US-born (30.3%) | AGE GROUP: aged ≤34 (n=26; 33.3%); aged 35-44 (n=26; | Female (100%, mother) | Parent (mother/guardian) | Routine HPV vaccination for | Self-report (by Mothers): HPV vaccine uptake (≥ 1 dose) in | Moderate risk |

| Study ID, First author, Publication year | Study type/ design | Year(s) of study | Location/country of study | Setting | Total number of participants/ sample size (study completers) | No. of immigrants/first-generation & descendants/ second-generation | Proportion of immigrants/first-generation & descendants/second-generation | Migratory status/ Reason for migration | Country/Region of origin/ Nativity/ Ethnicity(/race) | Age | Gender/ sex | Participant type | Intervention/ programme | Uptake of HPV vaccine | Study risk of bias scores |
|---|---|---|---|---|---|---|---|---|---|---|---|---|---|---|---|
| | sectional | 2009 (recruited) | | | ns of daughters aged 9–17) | participating mother) | | | NATIVITY/COUNTRY OF ORIGIN (Parent): Mexico-born, at least one parent (86.1%); US-born (13.9%). 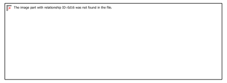 | 33.3%); aged ≥45 (n=26; 33.3%). | | | girls aged 11 (9)-12 and Catch-up vaccination for females aged 13-26 | daughters aged 9–17: 34.6% of total participating mothers [95% CI: 24.2–46.2]. Mothers Age – aged ≤34, vaccine uptake by daughters 34.6% vs aged 35-44, 46.2% vs ≥aged 45, 23.1%, p=0.22. Mothers Marital status – Married/living as Married, vaccine uptake by daughters 40.7% vs Not married 22.7%, p=0.14. Mothers Country of origin – **Mexico-born, vaccine uptake by daughters 38.7%** vs US-born 10.0%, p=0.08. Mothers Acculturation – Low Acculturation, vaccine uptake by daughters 36.4% vs High Acculturation 32.4%, p=0.71. Mothers Income - <$20K, vaccine uptake by daughters 31.6% vs $20K-35K, 26.3% vs >$35K, 50.0%, p=0.27. Mothers Occupation – Agriculture, vaccine uptake by | |

| Study ID, First author, Publication year | Study type/ design | Year(s) of study | Location/country of study | Setting | Total number of participants/ sample size (study completers) | No. of immigrants/first-generation & descendants/ second-generation | Proportion of immigrants/first-generation & descendants/second-generation | Migratory status/ Reason for migration | Country/Region of origin/ Nativity/ Ethnicity(/race) | Age | Gender/ sex | Participant type | Intervention/ programme | Uptake of HPV vaccine | Study risk of bias scores |
|---|---|---|---|---|---|---|---|---|---|---|---|---|---|---|---|
| | | | | | | | | | | | | | | daughters 30.0% vs Service/Technical 42.3% vs Homemaker 31.6% vs Other 23.1%, p=0.66. | |
| [45], Marlow LAV, 2009 | Quantitative, cross-sectional | July - August 2008 (data collection) | England | Community | N=950 (women aged ≥16 for HPV awareness study component). N=601 (women aged ≥16 for HPV vaccine acceptability study component). | n=591 | 62.2% | Not specified | NATIVITY/GENERATIONAL STATUS/ETHNICITY: MIGRANT TOTAL (n=591; 62.2% = 591/950). First generation (n=300; 31.6% = 300/950); Immigrant (n=291; 30.6%=291/950). White British (n=200): Second generation (94%); First generation (6%); migrant (0%). Indian (n=235): Second generation (26%)*; First generation (32%); migrant (43%). Pakistani (n=164): Second generation (15%)*; First generation (54%); migrant (32%). Bangladeshi (n=63): Second generation (6%)*; First generation (48%); migrant (46%). Caribbean (n=130): Second generation (35%)*; First generation (29%); migrant (35%). African (n=107): Second generation (24%)*; First generation (36%); migrant (40%). Chinese (n=51): Second generation (28%)*; First generation (33%); migrant (39%). [*p<0.05]<br><br>GENERATIONAL STATUS: | AGE: No average age data available. AGE GROUP: aged 25–34 (26%); aged 35–44 (28%). Note: The remaining data not available. AGE GROUP by ETHNICITY: White British (n=200): aged 16-24 (10%); aged 25-34 (29%); aged 35-44 (25%); aged 45-54 (17%); aged 55+ (21%). Indian (n=235): aged 16-24 (20%)*; aged 25-34 (29%); aged 35-44 (26%); aged 45-54 (12%); aged 55+ (15%). Pakistani (n=164): aged 16-24 (23%)*; aged 25-34 (24%); aged 35-44 (34%); aged 45-54 (10%); aged 55+ (10%)*. Bangladeshi (n=63): aged 16-24 (29%)*; aged 25-34 (24%); aged 35-44 (25%); aged 45-54 (16%); aged 55+ (6%)*. Caribbean (n=130): aged 16-24 (22%)*; aged 25-34 (24%); aged 35-44 (22%); aged 45-54 (15%); aged 55+ (17%). African (n=107): | Female (100%, girls/mothers) | Parent (mother); Recipient of vaccine (girl) | School-based (including scenario) | n/a | Low risk |

| Study ID, First author, Publication year | Study type/ design | Year(s) of study | Location/country of study | Setting | Total number of participants/ sample size (study completers) | No. of immigrants/first-generation & descendants/ second-generation | Proportion of immigrants/first-generation & descendants/second-generation | Migratory status/ Reason for migration | Country/Region of origin/ Nativity/ Ethnicity(/race) | Age | Gender/ sex | Participant type | Intervention/ programme | Uptake of HPV vaccine | Study risk of bias scores |
|---|---|---|---|---|---|---|---|---|---|---|---|---|---|---|---|
| | | | | | | | | | White British (n=200): Second generation (94%); First generation (n=12; 6%); Immigrant (n=0; 0%). Indian (n=235): Second generation (26%)*; First generation (n=75; 32%); Immigrant (n=101; 43%). Pakistani (n=164): Second generation (15%)*; First generation (n=89; 54%); Immigrant (n=52; 32%). Bangladeshi (n=63): Second generation (6%)*; First generation (n=30; 48%); Immigrant (n=29; 46%). Caribbean (n=130): Second generation (35%)*; First generation (n=38; 29%); Immigrant (n=46; 35%). African (n=107): Second generation (24%)*; First generation (n=39; 36%); Immigrant (n=43; 40%). Chinese (n=51): Second generation (28%)*; First generation (n=17; 33%); Immigrant (n=20; 39%). [*p<0.05] | aged 16-24 (20%)*; aged 25-34 (30%); aged 35-44 (35%); aged 45-54 (9%); aged 55+ (7%)*. Chinese (n=51): aged 16-24 (24%)*; aged 25-34 (18%); aged 35-44 (43%)*; aged 45-54 (16%); aged 55+ (0%)*. *p<0.05 | | | | | |
| [46], Remschmidt C, 2014 | Quantitative, cross-sectional | 2010 - 2012 | Germany | Household | N=823 (women, aged 20 - 25 in Germany) | n=116 | 14.1% | Not specified | NATIVITY: Migrants (14.1%); Non-migrants (85.9%). | AGE: Mean 22.6 years (SD=1.6) | Female (100%) | Recipient of vaccine (women aged 20-25). | Free of charge for all females aged 12-17 | Self-report: Initiation of HPV vaccine (≥1 dose): 30.3% (95% CI: 27.1-33.7) of total participants with HPV vaccination status information; Completion of HPV vaccine (full course): 26.7% (95% CI: 23.6-29.9). Initiation of HPV vaccine (≥1 dose): Current smoker – No 35.5% vs Yes 17.9%; Migrant background – No | Low risk |

| Study ID, First author, Publication year | Study type/ design | Year(s) of study | Location/country of study | Setting | Total number of participants/ sample size (study completers) | No. of immigrants/first-generation & descendants/ second-generation | Proportion of immigrants/first-generation & descendants/second-generation | Migratory status/ Reason for migration | Country/Region of origin/ Nativity/ Ethnicity(/race) | Age | Gender/ sex | Participant type | Intervention/ programme | Uptake of HPV vaccine | Study risk of bias scores |
|---|---|---|---|---|---|---|---|---|---|---|---|---|---|---|---|
| | | | | | | | | | | | | | | (non-migrants) 32.9% vs **Yes (migrants) 13.0%**; Educational status – Low 10.8% vs Medium 27.4% vs High 33.1%. | |
| [47], Rondy M, 2010 | Quantitative, cross-sectional | March–May 2009 | Netherlands | Multi-level | N=384,869 (girls) | n=24,643 | 6.4% | Not specified | NATIVITY: Foreign-born (6.4%); The Netherlands-born (15.3%); Unknown (78.3%).<br><br>COUNTRY OF ORIGIN (PARENTS):<br>The Netherlands – The Netherlands (n=58941; 15.3%);<br>The Netherlands – Morocco (n=320; 0.08%);<br>The Netherlands-Netherlands Antilles (n=413; 0.11%);<br>The Netherlands-Surinam (n=882; 0.23%);<br>The Netherlands-Turkey (n=455; 0.12%);<br>Morocco-Morocco (n=3881; 1.01%);<br>Netherlands Antilles-Netherlands Antilles (n=419; 0.11%);<br>Suriname-Suriname (n=1972; 0.51%);<br>Turkey-Turkey (n=3678; 0.96%);<br>The Netherlands-Other (n=5368; 1.39%);<br>Other-Other (n=7255; 1.89%);<br>Unknown (n=301285; 78.3%). | AGE: No average age data available.<br><br>AGE GROUP by BIRTH COHORT: Birth cohort 1993 (n=98,291; 25.5%); Birth cohort 1994 (n=97,918; 25.4%); Birth cohort 1995 (n=94,792; 24.6%); Birth cohort 1996 (n=93,868; 24.4%). Note: No disaggregated data available for migrants and non-migrants. | Female (100%, girls - in documentary review); Both gender? (stakeholders - questionnaire). | Recipient of vaccine (girl); Stakeholders (all regional coordinators of the HPV vaccination campaign at the The Community Health Services [CHS]). | HPV catch-up vaccination campaign implemented in March 2009 for girls born between 1993-1996 | Vaccination register 'Praeventis': Individual data – Birth cohort year 1999, 48.6% vs year 1994, 50.6% vs Year 199, 50.7% vs Year 1996, 49.7%. Previous MMR vaccination – No 14.5% vs One dose 32.9% vs Two dose 51.6%; Parents Country of origin – The Netherlands – The Netherlands 51.8% vs The Netherlands – Morocco 36.3% vs The Netherlands Antilles 47.2% vs The Netherlands – Suriname 43.8% vs The Netherlands – Turkey 44.0% vs **Morocco – Morocco 24.0%** vs The Netherlands Antilles – Netherlands Antilles 37.5% vs **Suriname – Suriname 44.2%** vs **Turkey-** | Low risk |

| Study ID, First author, Publication year | Study type/ design | Year(s) of study | Location/country of study | Setting | Total number of participants/ sample size (study completers) | No. of immigrants/first-generation & descendants/ second-generation | Proportion of immigrants/first-generation & descendants/second-generation | Migratory status/ Reason for migration | Country/Region of origin/ Nativity/ Ethnicity(/race) | Age | Gender/ sex | Participant type | Intervention/ programme | Uptake of HPV vaccine | Study risk of bias scores |
|---|---|---|---|---|---|---|---|---|---|---|---|---|---|---|---|
| | | | | | | | | | | | | | | **Turkey 37.6%** vs The Netherlands – Other 50.0% vs **Other – Other 44.9%** vs Unknown 50.3%. Distance girls' house to vaccination centre – 0km, 52.6% vs 0-4.99km, 0.0% vs 5-9.99km, 48.% vs =10km, 47.7%. Postcode level – Socioeconomic status (SES) – High SES 53.1% vs High average SES 50.7% vs Low average SES 49.4% vs Low SES 46.9%. | |
| [48], van Boetzelaer E, 2022 | Quantitative, cross-sectional | 23 September - 6 October 2019 (data collection) | Norway | Household | N=4,967 (women born between 1991-1996 who were offered free catch-up HPV vaccination between 1 November 2016-30 June 2019 in Norway) | n=540 (Foreign-born study participant). (Foreign-born with at least one caregiver: n=716). | 10.9% (Foreign-born study participant). (Foreign-born with at least one caregiver: 16.3%). | Not specified | NATIVITY (Study participant): Foreign-born (10.9%); Norway-born (89.1%) NATIVITY (Parent): Foreign-born with at least one caregiver (16.3%); Norway-born (83.7%). COUNTRY/REGION OF ORIGIN (Study participant): Norway (89.1%); EU, USA, Canada, Australia, New Zealand (4.3%); Asia, Africa, Latin America, Oceania, Europe (outside EU) (6.6%). COUNTRY/REGION OF ORIGIN (Parent): Norway both caregivers (83.7%); EU, USA, Canada, Australia, New Zealand with at least one caregiver (6.3%); Asia, Africa, Latin America, Oceania, Europe | AGE: Median 26 years (range 23-28). Note: AGE by HPV VACCINATION (n=4,967): No vaccination (Median 26 years; range 23-28 years); Partial vaccination (Median 26 years; range 23-28 years); Complete vaccination (Median 26 years; range 23-28 years). | Female (100%) | Recipient of vaccine (study participating woman); Parent (caregiver) | Free-of-charge, Catch-up HPV vaccination offered between 1 November 2016 - 30 June 2019 for women born in 1991-1996 | Self-report: Partial vaccination (1–2 doses), weighted %: Overall 6.5% [95% CI: 5.7-7.5]. Country of origin (Study participants) – Norway 5.8% [5.1-6.5] vs EU, USA, Canada, Australia, NZ 8.0% [5.0-12.5] vs Asia, Africa, Latin America, Oceania, Europe (outside EU) 8.1% [5.6-11.6]. Duration of residence in Norway for migrants (study participants) – 0-4 years 8.1% [5.0-12.9] vs 5-9 years | Low risk |

| Study ID, First author, Publication year | Study type/ design | Year(s) of study | Location/country of study | Setting | Total number of participants/ sample size (study completers) | No. of immigrants/first-generation & descendants/ second-generation | Proportion of immigrants/first-generation & descendants/second-generation | Migratory status/ Reason for migration | Country/Region of origin/ Nativity/ Ethnicity(/race) | Age | Gender/ sex | Participant type | Intervention/ programme | Uptake of HPV vaccine | Study risk of bias scores |
|---|---|---|---|---|---|---|---|---|---|---|---|---|---|---|---|
| | | | | | | | | | with at least 1 caregiver (outside EU) (10.0%). | | | | | 5.1% [1.9-12.9] vs ≥10 years 8.8% [5.9-12.9]. Country of origin (caregivers) – Both caregivers from Norway 5.5% [4.8-6.3] vs At least one caregiver from EU, USA, Canada, Australia, NZ 9.1% [5.4-15.1] vs At least one care giver from Asia, Africa, Latin America, Oceania, Europe (outside EU) 7.2% [4.7-11.0]. Marital status – Single 5.5% [4.1-7.2] vs In a relationship 6.9% [5.1-9.4] vs Cohabiting 6.8% [5.6-8.3] vs Married 7.7% [5.1-11.6] vs Separated 7.3% [1.1-37.6]. Children – Yes 7.4% [5.6-9.7] vs No 6.3% [5.3-7.4]. Highest completed education – Primary 7.6% [5.4-10.7] vs Secondary 6.4% [5.0-8.2] vs University or College 6.2% [5.1-7.5]. Household income after tax in NOK – P10 214K 6.8% [5.3-8.6] vs P20 266K 6.7% [4.3-10.3] vs P30 305K 6.0% [3.7-9.5] vs | |

| Study ID, First author, Publication year | Study type/ design | Year(s) of study | Location/country of study | Setting | Total number of participants/ sample size (study completers) | No. of immigrants/first-generation & descendants/ second-generation | Proportion of immigrants/first-generation & descendants/second-generation | Migratory status/ Reason for migration | Country/Region of origin/ Nativity/ Ethnicity(/race) | Age | Gender/ sex | Participant type | Intervention/ programme | Uptake of HPV vaccine | Study risk of bias scores |
|---|---|---|---|---|---|---|---|---|---|---|---|---|---|---|---|
| | | | | | | | | | | | | | | P40 339K 6.5% [3.8-10.7] vs P50 372K 5.8% [3.3-10.1] vs P60 408K 6.2% [3.6-10.6] vs P70 450K 4.8 [2.8-8.1] vs P80 507K 7.3% [4.5-11.7] vs P90 606K 6.4% [3.9-10.3] vs P100 >606001 9.4% [5.6-15.5]. Complete vaccination (3 doses): Overall 63.4% [95% CI: 61.6-65.1]. Country of origin (Study participants) – Norway 70.1% [68.7-71.5] vs EU, USA, Canada, Australia, NZ 52.2% [45.6-58.9] vs Asia, Africa, Latin America, Oceania, Europe (outside EU) 48.0% [42.6-53.4]. Duration of residence in Norway for migrants (study participants) – 0-4 years 41.1% [34.3-55.4] vs 5-9 years 44.2% [33.6-55.4] vs ≥10 years 57.3% [51.3-63.1]. Country of origin (caregivers) – Both caregivers from Norway 71.9% [70.3-73.4] vs At least one caregiver from EU, USA, | |

| Study ID, First author, Publication year | Study type/ design | Year(s) of study | Location/country of study | Setting | Total number of participants/ sample size (study completers) | No. of immigrants/first-generation & descendants/ second-generation | Proportion of immigrants/first-generation & descendants/second-generation | Migratory status/ Reason for migration | Country/Region of origin/ Nativity/ Ethnicity(/race) | Age | Gender/ sex | Participant type | Intervention/ programme | Uptake of HPV vaccine | Study risk of bias scores |
|---|---|---|---|---|---|---|---|---|---|---|---|---|---|---|---|
| | | | | | | | | | | | | | | Canada, Australia, NZ 58.2% [50.4-65.6] vs At least one care giver from Asia, Africa, Latin America, Oceania, Europe (outside EU) 51.9% [46.3-57.6]. Marital status – Single 66.6% [63.5-69.6] vs In a relationship 67.8% [63.8-71.6] vs Cohabiting 64.8% [62.0-67.4] vs Married 45.9% [40.4-51.5] vs Separated 28.7% [11.8-54.8]. Children – Yes 47.0% [43.2-5.08] vs No 67.8% [65.8-69.7]. Highest completed education – Primary 44.7% [40.3-49.1] vs Secondary 56.9% [53.7-60.1] vs University or College 73.6% [71.3-75.7]. Household income after tax in NOK – P10 214K 66.7% [63.5-69.6] vs P20 266K 55.4% [50.0-60.7] vs P30 305K 61.4% [55.2-67.3] vs P40 339K 57.0% [50.5-63.3] vs P50 372K 59.9% [53.5-66.0] vs P60 408K 66.0% [59.4-72.1] vs P70 450K 72.4 [66.5- | |

| Study ID, First author, Publication year | Study type/ design | Year(s) of study | Location/country of study | Setting | Total number of participants/ sample size (study completers) | No. of immigrants/first-generation & descendants/ second-generation | Proportion of immigrants/first-generation & descendants/second-generation | Migratory status/ Reason for migration | Country/Region of origin/ Nativity/ Ethnicity(/race) | Age | Gender/ sex | Participant type | Intervention/ programme | Uptake of HPV vaccine | Study risk of bias scores |
|---|---|---|---|---|---|---|---|---|---|---|---|---|---|---|---|
| | | | | | | | | | | | | | | 77.7] vs P80 507K 66.7% [60.6-72.3] vs P90 606K 72.4% [66.4-77.8] vs P100 >606001 66.9% [59.0-74.0]. | |
| [49], Lindsay AC, 2020 | Quantitative, cross-sectional | January - April 2019 (survey data collection) | USA | Community | N=47 (Brazilian immigrant parents, each representing a unique family, with at least one child aged 11–19, lived in Massachusetts; and had resided in US for at least 12 months) | n=47 | 100.0% | Not specified | COUNTRY OF ORIGIN: Brazil (100%).<br><br>STATE OF ORIGIN (Brazilian state):<br>Minas Gerais 66.0% (Fathers 57.1%; Mothers 73.1%);<br>São Paulo 4.3% (Fathers 4.8%; Mothers 3.8%);<br>Paraná 6.4% (Fathers 9.5%; Mothers 3.8%);<br>Santa Catarina 8.5% (Fathers 9.5%; Mothers 7.7%);<br>Rio de Janeiro 2 4% (Fathers 0%; Mothers 7.7%);<br>Espírito Santo 8.5% (Fathers 14.3%; Mothers 3.8%);<br>Mato Grosso 2.1% (Fathers 4.8%; Mothers 0%). | AGE (Parent): Mean 45.3 years (SD=8.6).<br>AGE (Mother): Mean 45.6 (SD=9.3);<br>AGE (Father): Mean 44.9 (SD=7.7). | Both gender. Female (55.3%, mothers); Male (44.7%, fathers). | Parents (migrant parents) | Recommended HPV vaccination for females aged 11-26 and males aged 11-21. Free access to healthcare via government-sponsored health insurance (i.e., MassHealth) | n/a | Low risk |
| [50], Lindsay AC, 2020 | Quantitative, cross-sectional | June - October 2019 | USA | Community | N=56 (Central American immigrant parents, who have ≥1 child aged 11-19) | n=54 | 96.4% | Not specified | NATIVITY (ALL): Foreign-born (96.4%); US-born (3.6%).<br>COUNTRY OF ORIGIN (among FOREIGN-BORN): El Salvador (50.0%); Guatemala (25.9%); Honduras (22.2%); Panama (1.9%).<br><br>NATIVITY (FATHER): Foreign-born (95.8%); US-born (4.2%).<br>COUNTRY OF ORIGIN (among FOREIGN-BORN FATHER): El Salvador | AGE (All): Mean 43.2 years (SD=6.4).<br>AGE (Mother): Mean 45.8 years (SD=7.3);<br>AGE (Father): Mean 39.6 years (SD=8.2). | Both gender. Female (57.1%, mothers); Male (42.9%, fathers). | Parents (mother & father) | Routine HPV vaccination for girls and boys aged 11-12 and Catch-up vaccination for females aged 13-26 and males | Self-report: HPV vaccine (≥1 dose): 85.7% of parents' (those who had heard of the HPV vaccine) children. Fathers reported 64.3% vs Mothers 96.4% (p = 0.06). | Moderate risk |

| Study ID, First author, Publication year | Study type/ design | Year(s) of study | Location/country of study | Setting | Total number of participants/ sample size (study completers) | No. of immigrants/first-generation & descendants/ second-generation | Proportion of immigrants/first-generation & descendants/second-generation | Migratory status/ Reason for migration | Country/Region of origin/ Nativity/ Ethnicity(/race) | Age | Gender/ sex | Participant type | Intervention/ programme | Uptake of HPV vaccine | Study risk of bias scores |
|---|---|---|---|---|---|---|---|---|---|---|---|---|---|---|---|
| | | | | | | | | | (56.6%); Guatemala (21.7%); Honduras (21.7%); Panama (0%).<br><br>NATIVITY (MOTHER): Foreign-born (96.8%); US-born (3.2%).<br>COUNTRY OF ORIGIN (among FOREIGN-BORN MOTHER): El Salvador (45.2%); Guatemala (29.0%); Honduras (22.6%); Panama (3.2%). | | | | aged 13-21 | | |
| [51], Bhatta MP, 2020 | Quantitative, cross-sectional | 2014 | Nepal | Women's health camp in Jhapa District in eastern Nepal | N=630 (Married Nepali women n=540 [85.7%] and Married Bhutanese refugee women n=90 [14.3%], living in eastern Nepal, 2014). | n=90 | 14.3% | Refugee | NATIVITY/COUNTRY OF ORIGIN: Nepali women (85.7%); Bhutanese refugee women (14.3%). | AGE: Mean 38.7 years (SD=8.3) [Mean 38.8 years (SD=8.2), according to Abstract section]; Median 38.0 years (range 19–69).<br>AGE (Nepali): Mean 39.5 years (SD=8.2); AGE (Bhutanese): Mean 34.1 years (SD=7.3). [p < 0.001].<br><br>AGE GROUP (All): aged 19-34 (n=238; 37.8%); aged 35-44 (n=217; 34.4%); aged 45-69 (n=175; 27.8%).<br>AGE GROUP (Nepali): aged 19-34 (n=184; 34.1%); aged 35-44 (n=189; 35.0%); aged 45-69 (n=167; 30.9%);<br>AGE GROUP (Bhutanese): aged 19-34 (n=54; 60.0%); aged 35-44 (n=28; 31.1%); aged 45-69 (n=8; 8.9%). [p<0.0001] | Female (100%) | Married women | Free-of-charge HPV vaccination (scenario) | n/a | Low risk |

| Study ID, First author, Publication year | Study type/ design | Year(s) of study | Location/country of study | Setting | Total number of participants/ sample size (study completers) | No. of immigrants/first-generation & descendants/ second-generation | Proportion of immigrants/first-generation & descendants/second-generation | Migratory status/ Reason for migration | Country/Region of origin/ Nativity/ Ethnicity(/race) | Age | Gender/ sex | Participant type | Intervention/ programme | Uptake of HPV vaccine | Study risk of bias scores |
|---|---|---|---|---|---|---|---|---|---|---|---|---|---|---|---|
| [52], Adjei Boakye E, 2018 | Quantitative, cross-sectional | 2014-2015 (data collection) | USA | Household | N=7,588 (young adults aged 18–26) | n=1,114 | 14.7% | Not specified | NATIVITY: US-born (87.3%); Foreign-born (12.7%).<br>RACE/ETHNICITY.<br>Total: Non-Hispanic White (57.3%); Non-Hispanic Black (15.0%); Hispanic (21.5%); Non-Hispanic Other (6.2%).<br>US-born: Non-Hispanic White (62.9%); Non-Hispanic Black (15.9%); Hispanic (17.2%); Non-Hispanic Other (4.0%).<br>Foreign-born: Non-Hispanic White (18.5%); Non-Hispanic Black (9.1%); Hispanic (51.0%); Non-Hispanic Other (21.4%).[p<0.0001] | AGE (All): Mean 22.5 years (SD=2.5).<br>AGE (US-born): Mean 22.4 years (SD=2.5);<br>AGE (Foreign-born): Mean 22.8 years (SD=2.5) [p<0.0001] | Both gender. Female (50.2%); Male (49.8%). | Recipient of vaccine | Catch-up | Self-report: Initiation of HPV vaccine: 26.8% of total participants [young adults aged 18–26] (**14.5% foreign-born** vs 28.6%US-born; p<.0001); Completion overall: 15.6% of total participants (**7.6% foreign-born** vs 16.8% US-born; p<.0001); Completion among initiators: 64.2% among initiators (**61.9% foreign-born** vs 64.4% US-born; p=0.5842). | Low risk |
| [53], Adjei Boakye E, 2019 | Quantitative, cross-sectional | 2014-2017 (data collection); July 2018 (data analysis) | USA | Household | N=14,056 (men, aged 18–34) | n=2,396 | 17.0% | Not specified | NATIVITY: US-born (83.0%); Foreign-born (17.0%)<br><br>RACE/ETHNICITY.<br>Total: Non-Hispanic White (57.9%); Non-Hispanic Black (13.4%); Hispanic (21.8%); Non-Hispanic Other (6.9%).<br>US-born men: Non-Hispanic White (66.5%); Non-Hispanic Black (13.9%); Hispanic (15.7%); Non-Hispanic Other (3.9%).<br>Foreign-born men: Non-Hispanic White (15.8%); Non-Hispanic Black (11.2%); Hispanic (51.4%); Non-Hispanic Other (21.6%).<br>[p<0.0001]<br><br>REGION OF ORIGIN: | AGE (All): Mean 26.5 years (SD=4.8).<br>AGE (US-born men): Mean 26.3 years (SD=4.8);<br>AGE (Foreign-born men): Mean 27.6 years (SD=4.5).<br>[p<0.0001] | Male (100%) | Recipient of vaccine | Routine HPV vaccination for girls and boys aged 11-12 and Catch-up vaccination for females aged 13-26, and males aged 13-21 and for immune-compromised men or | Self-report: Initiation of HPV vaccine: 9.9% of total participants [adult men aged 18–34] (4.5% foreign-born men vs 11.0% US-born men; p<0.0001); Completion: 3.3% of total participants (1.7% foreign-born men vs 3.7% US-born men; p=0.0011). | Low risk |

| Study ID, First author, Publication year | Study type/ design | Year(s) of study | Location/country of study | Setting | Total number of participants/ sample size (study completers) | No. of immigrants/first-generation & descendants/ second-generation | Proportion of immigrants/first-generation & descendants/second-generation | Migratory status/ Reason for migration | Country/Region of origin/ Nativity/ Ethnicity(/race) | Age | Gender/ sex | Participant type | Intervention/ programme | Uptake of HPV vaccine | Study risk of bias scores |
|---|---|---|---|---|---|---|---|---|---|---|---|---|---|---|---|
| | | | | | | | | | Central America (49.2%); South America (7.1%); Europe (8.5%); Africa (6.5%); Indian Subcontinent (8.7%); Asia (6.2%); Southeast Asia (5.8 %); Other [Middle East, unknown](8.0%). | | | | men who have sex with men up to aged 26 | | |
| [54], Anuforo B, 2022 | Quantitative, cross-sectional | October 2018 - March 2020 (recruitment) | USA | Community | N=77 (Parents of adolescents, aged 11–18) | n=31 | 40.3% | Not specified | NATIVITY (PARENT): US-born (59.7%); Foreign-born (40.3%).<br><br>ETHNICITY/RACE (PARENT): US-born Hispanic (26.1%); Non-Hispanic White (41.3%); Non-Hispanic Black (28.3%); Non-Hispanic Asian (2.2%); Other/Multi-race/Missing (2.2%); Foreign-born Hispanic (16.1%); Non-Hispanic White (12.9%); Non-Hispanic Black (3.2%); Non-Hispanic Asian (61.3%); Other/Multi-race/Missing (6.5%) [p<0.01]. | AGE GROUP (Parent, US-BORN): aged 30-40 (21.7%); aged 41-50 (54.8%); aged >50 (25.8%); AGE GROUP (Parent, Foreign-born): aged 30-40 (19.4%); aged 41-50 (52.2%); aged >50 (26.1%). [p=0.96].<br><br>AGE GROUP (Participant's adolescent, US-born): aged 11-12 (26.1%); aged 13-15 (34.8%); aged 16-18 (39.1%); AGE GROUP (Participant's adolescent, Foreign-born): aged 11-12 (19.4%); aged 13-15 (48.4 %); aged 16-18 (32.3%). [p=0.48].<br><br>AGE (Participant's adolescent): Mean 14 years. | Both gender. GENDER (PARENT): US-BORN – Female (89.1%); Male (8.7%). FOREIGN-BORN – Female (77.4%); Male (22.6%) [p=0.18]. GENDER (PARTICIPANT'S ADOLESCENT): US-BORN - Female (56.5%); Male (43.5%). FOREIGN-BORN - Female (77.4%); Male (22.6%) [p=0.06]. | Parents (of adolescents, aged 11–18) | Routine HPV vaccination for adolescent girls (since 2006) and adolescent boys (since 2009) | Self-report: Initiation of HPV vaccine: 57.1% of total participants who are parents of adolescents (Foreign-born 29.0% vs US-born 52.2%; p=0.04). Foreign-born parents of adolescents initiating the HPV vaccine aOR 0.3 [95% CI: 0.1, 0.9] compared to US-born parents [reference] after demographic characteristics adjustment. | Low risk |
| [55], Kepka D, 2018 | Quantitative, cross-sectional | May 2014 - February 2015 (recruited) | USA | Community | N=228 (adult parents/ legal guardians/ caregivers (aged ≥18), who are vaccination decision-makers for teens, aged | n=154 (Participants).<br><br>(Participants' parents: n=176) | 67.54% (Participants).<br><br>(Participants' parents: 77.19%) | African refugees. But, not clear about Hispanic/Latino | RACE/ETHNICITY: African American (7.46%); African immigrant (17.12%); American Indian/Alaskan Native (10.09%); Hispanic/Latino (28.07%); Native Hawaiian/Pacific Islander (30.70%); Other (includes | AGE: No average age data available. AGE GROUP: aged <35 (n=42; 18.42%); aged 35-50 (n=148; 64.91%); aged >50 years (n=35; 15.35%). | Both gender. Female (71.05%); Male (28.07%). | Parents/ legal guardians/ caregivers (aged ≥18, of adolescent | n/a | n/a | Moderate risk |

| Study ID, First author, Publication year | Study type/ design | Year(s) of study | Location/country of study | Setting | Total number of participants/ sample size (study completers) | No. of immigrants/first-generation & descendants/ second-generation | Proportion of immigrants/first-generation & descendants/second-generation | Migratory status/ Reason for migration | Country/Region of origin/ Nativity/ Ethnicity(/race) | Age | Gender/ sex | Participant type | Intervention/ programme | Uptake of HPV vaccine | Study risk of bias scores |
|---|---|---|---|---|---|---|---|---|---|---|---|---|---|---|---|
| | | | | | 11–17, from African American, African refugee, American Indian/Alaskan Native, Hispanic/Latino, and Native Hawaiian/Pacific Islander community groups in Utah) | | | and others. | multiracial) (3.07%).<br><br>NATIVITY: US-born (31.58%); Foreign-born (67.54%).<br><br>NATIVITY (PARENT): US-born (20.61%); Other (77.19%). | | | s aged 11–17). | | | |
| [56], Yi JK, 2013 | Quantitative, cross-sectional | n/a | USA | Community | N=113 (Vietnamese-American women, aged ≥18, and living in the metropolitan area of Houston, Texas) | n=71 | 62.8% | Not clear (Student, n=65; 57.5%) | COUNTRY OF ORIGIN: Vietnam-born (58%); US-born (37%); Other (5%). | AGE: Mean 22 years (SD=3).<br>Note: No disaggregated data for migrants and non-migrants. | Female (100%) | Recipient of vaccine (young women) | n/a | Self-report: HPV vaccine receipt: 14.2% [16/113] of total participants (Foreign-born [Vietnam and other] 11.3% [8/71] vs US-born 19.0% [8/42]); HPV vaccine completion [3 doses]: 9 %. | Low risk |
| [57], Ashing KT , 2017 | Quantitative, cross-sectional | 2009-2011 | USA | Community | N=383<br><br>(n=201 Hispanic [US-born Latina and Latina migrants]; n=182 non-Hispanic Blacks [US-born African Americans and Black migrants]). | n=197 | 51.4% | Not specified | NATIVITY: US-born (48.6%); Foreign-born (51.4%).<br><br>REGION/COUNTRY OF ORIGIN: US (48.6%); Latin America or the Caribbean (51.4%).<br><br>ETHNICITY:<br>Non-Hispanic Black: AA born in the US (33.7%); Black immigrants (13.8%).<br>Hispanic: Latinas born in the US (14.9%); Latina immigrants (37.6%). | AGE (adults ages ≥ 18 years) by ETHNICITY:<br>AA Born in US: Mean 46.32 years (SD=14.32); Black Immigrant: Mean 35.79 years (SD=10.64). [p<0.001].<br>Latina born in US: Mean 34.19 years (SD=13.49); Latina immigrants: Mean 45.13 years (SD=9.16). [p<0.001]. | Female (100%) | Recipient of vaccine (adult women); Parent (mother) | n/a | n/a | Moderate risk |
| [58], Glenn BA, 2015 | Quantitative, cross- | January 2009 - January 2010 | USA | Community/Clinic | N=490 (mothers/caregivers n=481; | n=432 | 88.2% | Not specified | NATIVITY (Study participant - Mother/Caregiver): Foreign-born (88%); US-born (12%). | AGE (Mother/caregiver): Mean 44 years (SD=7.22). | Female (100%) | Parent (Participating mother/car | Vaccines for Children (VFC) | n/a | Moderate risk |

| Study ID, First author, Publication year | Study type/ design | Year(s) of study | Location/country of study | Setting | Total number of participants/ sample size (study completers) | No. of immigrants/first-generation & descendants/ second-generation | Proportion of immigrants/first-generation & descendants/second-generation | Migratory status/ Reason for migration | Country/Region of origin/ Nativity/ Ethnicity(/race) | Age | Gender/ sex | Participant type | Intervention/ programme | Uptake of HPV vaccine | Study risk of bias scores |
|---|---|---|---|---|---|---|---|---|---|---|---|---|---|---|---|
| | sectional | (eligibility screening) | | | adolescent girls n=487). | | | | RACE/ETHNICITY: Latina (52%); Chinese (20%); Korean (13%); African American (8%); Other race (Non-Hispanic white, multiracial, or from other Asian subgroups, 7%). | AGE (Adolescent girl): Mean 14 years (SD=2.84). | | egiver); Recipient of vaccine (Adolescent girls). | programme (free-of-charge or low cost) at a county-affiliated immunisation clinic | | |
| [59], Kepka D, 2018 | Quantitative, cross-sectional | The 1st phase (focus group + survey): May-October 2014. The 2nd phase (survey only): October 2014–February 2015. | USA | Community | N=228 (Parents/ legal guardians/ caregivers (aged ≥18) of teens aged 11–17: n=93 from the 1st phase; n=135 from the 2nd phase) | n=154 (Participants). (Participant's parents: n=176) | 67.54% (Participants).<br><br>(Participants' parents: 77.19%) | African immigrant/refugee (17.12%). But, not clear about Hispanic/Latino and others. | NATIVITY (Caregiver): US-born (31.58%); Foreign-born (67.54%). NATIVITY (Caregiver's parent): US-born (20.61%); Foreign-born (77.19%). REGION OF ORIGIN (Caregiver): US (n=74/226; 32.7%); Latin American countries (n=65/226; 28.8%); Pacific Islands (n=45/226; 20.0%); African Countries (n=39/226; 17.3%); Australia (n=3/226; 1.3%). COUNTRY OF ORIGIN (Caregiver): US-born (32.7%); Mexico (19.5%); Peru (2.7%); Guatemala (1.8%); Argentina (1.3%); El Salvador (0.9%); Honduras (0.9%); Colombia (0.4%); Ecuador (0.4%); Venezuela (0.4%); Dominican Republic (0.4%); Tonga (18.6%); Samoa (0.9%); Vanuatu (0.4%); African country not specified (0.9%); Burundi (7.5%); Congo (4.9%); Rwanda (2.7%); Liberia (0.9%); Tanzania (0.4%); Australia (1.3%). ETHNICITY/RACE (Caregiver): African American (7.46%); African | AGE: Mean 43.09 years (SD=10.19; range 18–74). AGE GROUP: aged <35 (n=42; 18.42%); aged 35-50 (n=148; 64.91%); aged >50 (n=35; 15.35%). Note: No disaggregated data available for migrants and non-migrants. | Both gender. Female (71.05%); Male (28.07%). | Parents/ legal guardians/ caregivers (aged ≥18, vaccination decision-makers for teens aged 11–17 years). | n/a | n/a | Moderate risk |

| Study ID, First author, Publication year | Study type/ design | Year(s) of study | Location/country of study | Setting | Total number of participants/ sample size (study completers) | No. of immigrants/first-generation & descendants/ second-generation | Proportion of immigrants/first-generation & descendants/second-generation | Migratory status/ Reason for migration | Country/Region of origin/ Nativity/ Ethnicity(/race) | Age | Gender/ sex | Participant type | Intervention/ programme | Uptake of HPV vaccine | Study risk of bias scores |
|---|---|---|---|---|---|---|---|---|---|---|---|---|---|---|---|
| | | | | | | | | | immigrant/refugee (17.12%); American Indian/Alaskan Native (10.09%); Hispanic/Latino (28.07%); Native Hawaiian/Pacific Islander (30.70%); Other, including multiracial (3.07%). | | | | | | |
| [60], Colón-López V, 2015 | Quantitative, cross-sectional | 2013 | Puerto Rico | Clinic (Federal Qualified Health Clinic [FQHC]) | N=60 (Immigrant Dominican parents of adolescent sons, aged 9-17) | n=60 | 100.0% | Not specified | COUNTRY OF ORIGIN: Dominican Republic (100%). | AGE (Parent): Mean 38.6 years (SD=7.2). AGE (Youngest son): Mean 12.9 years (SD=2.6 years; range: 9-17). | Both gender (parents, but their child is male). Female (91.7%); Male (8.3%). | Parents/guardians | Vaccines for Children (VFC) programme at Federally Qualified Health Centers (FQHCs) | Self-report (by Dominican immigrant parents): Receipt of HPV vaccine (1≥ dose) in Son(s): 31.7% of total participants; Receipt of HPV vaccine in Daughter(s) (aged 9–26): 26.7%. | High risk |
| [61], Lee HY, 2018 | Quantitative, cross-sectional | 2016 (recruitment) | USA | Community | N=243 (Korean American migrant women [first-generation immigrant], aged 19-85) | n=243 | 100.0% | Not specified | COUNTRY OF ORIGIN/ ETHNICITY/ GENERATIONAL STATUS: Korean American (first generation, 100%). | AGE GROUP (n=235): aged 19-30 (n=56; 24%); aged 31-45 (n=80; 34%); aged 46-60 (n=45; 19%); aged ≥61 (n=54; 23%). | Female (100%) | Not explicitly mentioned, but potentially both Recipient of vaccine and Caregivers with a broad range of age groups (aged 19-85). | n/a | n/a | Low risk |
| [62], Cofie LE, 2018 | Quantitative, cross-sectional | 2016 (data analysis) | USA | Household | N=15,000 (women aged 18-35) | n=2,857 | 19.0% | Not specified | NATIVITY: Foreign-born (19.0%); US-born (81.0%).<br><br>REGION OF ORIGIN: US: Total (84.03%); US-born 100%. Mexico, Central America, Caribbean island: Total (7.69%); Foreign-born | AGE: No average age data available. AGE GROUP (All): aged 18-26 (n=6,289; 45.03%); aged 27-30 (n=3,862; 25.03%); aged 31-35 (n=4,849; 29.94%). | Female (100%) | Not explicitly mentioned, but potentially both Recipient of vaccine and | n/a | Self-report: Initiation of HPV vaccine: 27.56% of total participants (14.14% [12.42-15.86] Foreign-born vs 30.12% [28.82-31.42] US- | Low risk |

| Study ID, First author, Publication year | Study type/ design | Year(s) of study | Location/country of study | Setting | Total number of participants/ sample size (study completers) | No. of immigrants/first-generation & descendants/ second-generation | Proportion of immigrants/first-generation & descendants/second-generation | Migratory status/ Reason for migration | Country/Region of origin/ Nativity/ Ethnicity(/race) | Age | Gender/ sex | Participant type | Intervention/ programme | Uptake of HPV vaccine | Study risk of bias scores |
|---|---|---|---|---|---|---|---|---|---|---|---|---|---|---|---|
| | | | | | | | | | 48.14% (95% CI 45.60 – 50.68).<br>South America: Total (0.89%); Foreign-born 5.54% (95% CI 4.66 – 6.42).<br>Europe: Total (1.36%); Foreign-born 8.51% (95% CI 6.86 – 10.17).<br>Africa: Total (0.92%); Foreign-born 5.77% (95% CI 4.79 – 6.74).<br>Indian subcontinent: Total (1.32%); Foreign-born 8.25% (95% CI 6.89 – 9.60).<br>Asia: Total (1.39%); Foreign-born 8.70% (95% CI 7.30 – 10.09).<br>Southeast Asia: Total (1.14%); Foreign-born 7.12% (95% CI 6.07 – 8.17).<br>Other (including Russia and Middle East): Total (1.27%); Foreign-born 7.97% (95% CI 6.40 – 9.55).<br><br>RACE/ETHNICITY:<br>Non-Hispanic white: Total (n=7,951; 59.81%); US-born 67.93% (95% CI 66.68 – 69.18); Foreign-born 17.10% (95% CI 14.12 – 19.38).<br>Hispanic: Total (n=3,484; 17.92%); US-born 11.84% (95% CI 11.16 – 12.51); Foreign-born 49.87% (95% CI 47.30 – 52.44).<br>Black: Total (n=2,526; 16.33%); US-born 17.71% (95% CI 16.65 – 18.76); Foreign-born 9.08% (95% CI 7.80 – 10.37).<br>Asian: Total (n=1,039; 5.95%); US-born 2.53% (95% CI 2.20 – 2.86); Foreign-born 23.94% (95% CI 21.67 – 26.21).<br>X2: <0.0001 | AGE GROUP (US-born): aged 18-26 (47.04%; 95% CI 45.59 – 48.48); aged 27-30 (24.60%; 95% CI 23.67 – 25.54); aged 31-35 (28.36%; 95% CI 27.28 – 29.43).<br>AGE GROUP (Foreign-born): aged 18-26 (34.47%; 95% CI 32.35 – 36.59); aged 27-30 (27.24%; 95% CI 25.42 – 29.06); aged 31-35 (38.29%; 95% CI 36.06 – 40.51).<br>[X2: <0.0001]. | | Caregiver with a range of age groups (aged 18-35). | | born; p<0.0001);<br>Completion (3 doses): 8.77% of total participants (8.12% [4.53-11.71] Foreign-born vs 8.82% [7.49-10.16] US-born; p=0.0609). | |

| Study ID, First author, Publication year | Study type/ design | Year(s) of study | Location/country of study | Setting | Total number of participants/ sample size (study completers) | No. of immigrants/first-generation & descendants/ second-generation | Proportion of immigrants/first-generation & descendants/second-generation | Migratory status/ Reason for migration | Country/Region of origin/ Nativity/ Ethnicity(/race) | Age | Gender/ sex | Participant type | Intervention/ programme | Uptake of HPV vaccine | Study risk of bias scores |
|---|---|---|---|---|---|---|---|---|---|---|---|---|---|---|---|
| [63], Cofie LE, 2022 | Quantitative, cross-sectional | 2019 (Data analysed in 2019 using the 2013-2017 NHIS survey data). | USA | Household | N=5,246 (Black adults, aged 18-37) | n=543 | 10.4% | Not specified | NATIVITY/ETHNICITY: Foreign-born Black (10.4%); US-born Black (89.6%).<br>REGION OF ORIGIN (Foreign-born only): Africa (54.5%); Mexico/CA/CI/SA (Central America/Caribbean Island/South America, 33.1%); Other (Europe, Russia, Middle East, Asia, SE Asia, and Elsewhere, 12.4%). | AGE: No average age data available.<br><br>AGE AT TIME OF SURVEY:<br>All (n=5246): aged <21 (n=1271; 24.8%); aged 22-26 (n=1285; 25.3%); aged >26 (n=2690; 49.9%).<br>US-born (n=4703): aged <21 (n=1166; 25.3%); aged 22-26 (n=1175; 25.8%); aged >26 (n=2362; 48.9%).<br>Foreign-born (n=543): aged <21 (n=105; 20.8%); aged 22-26 (n=110; 20.9%); aged >26 (n=328; 58.4%).<br><br>AGE AT HPV VACCINATION ELIGIBILITY:<br>All (n=5246): aged ≤17 (n=2096; 42.7%); aged 18-26 (n=3150; 57.3%).<br>US-born (n=4703): aged ≤17 (n=1933; 43.9%); aged 18-26 (n=2770; 56.1%).<br>Foreign-born (n=543): aged ≤17 (n=163; 33%); aged 18-26 (n=380; 67%). | Both gender. Female (62.5%); Male (37.5%);<br>US-born female (63.4%); US-born male (36.6%);<br>Foreign-born female (55.4%); Foreign-born male (44.6%). | Recipient of vaccine | n/a | Self-report: Initiation of HPV vaccine: 21.7% of total participants (14.6% Foreign-born vs 22.5% US-born); Completion (≥3 doses): 11.5% of total participants (8.5% Foreign-born vs 11.8% US-born). | Low risk |
| [64], Hernandez ND, 2019 | Quantitative, cross-sectional | 2011-2012 | USA | College | N=187 (Unvaccinated Latina college female students in USA) | n=61 (Foreign-born) | 32.6 % (Foreign-born) | Not clear (College students) | NATIVITY/ GENERATIONAL STATUS:<br>Foreign-born (33%); US-born (67%), with foreign-born participants' parents (74%, second generation).<br><br>NATIVITY (PARENT): | AGE: Mean 24.8 years (SD=7.1). | Female (100%) | Recipient of vaccine | Catch-up HPV vaccination | n/a | Low risk |

| Study ID, First author, Publication year | Study type/ design | Year(s) of study | Location/country of study | Setting | Total number of participants/ sample size (study completers) | No. of immigrants/first-generation & descendants/ second-generation | Proportion of immigrants/first-generation & descendants/second-generation | Migratory status/ Reason for migration | Country/Region of origin/ Nativity/ Ethnicity(/race) | Age | Gender/ sex | Participant type | Intervention/ programme | Uptake of HPV vaccine | Study risk of bias scores |
|---|---|---|---|---|---|---|---|---|---|---|---|---|---|---|---|
| | | | | | | | | | Foreign-born, including "Not sure" (74%); US-born (26%). | | | | | | |
| [65], De P, 2017 | Quantitative, cross-sectional | n/a (could not locate - but used NHIS 2013 data) | USA | Household | N = 34,557 | n/a | n/a | Not specified | ETHNICITY/RACE:<br>a) Foreign-born, All – White 60.5% (95% CI: 0.56-0.65), African American 11.2% (0.08-0.14), Asian 24.8% (0.21-0.29), All other race 3.5% (0.02-0.05); b-ii) Foreign-born, Male - White 60.4% (0.54-0.67), African American 10.8% (0.07-0.15), Asian 25.4% (0.19-0.31), All other race 3.3% (0.02-0.05); b-iii) Foreign-born, Female - White 60.5% (0.54-0.67), African American 11.6% (0.07-0.16), Asian 24.2% (0.19-0.30), All other race 3.7% (0.00-0.07).<br>b) US-born, All – White 77.4% (95% CI: 0.75-0.79), African American 15.5% (0.14-0.17), Asian 2.8% (0.02-0.03), All other race 4.2% (0.04-0.05); a-ii) US-born, Male - White 78.9% (0.77-0.81), African American 13.7% (0.12-0.16), Asian 3.1% (0.02-0.04), All other race 4.3% (0.03-0.05); a-iii) US-born, Female - White 76.1% (0.74-0.78), African American 17.1% (0.15-0.19), Asian 2.6% (0.02-0.03), All other race 4.1% (0.03-0.05). | AGE: No average age data available; but those aged 18-26 (current recommended upper-limit for receiving HPV vaccine). | Both gender | Recipient of vaccine (young adults aged 18-26 = current recommended upper-limit for receiving HPV vaccine). | n/a | Self-report: Initiation of HPV vaccine: 17.2% [0.12-0.22] Foreign-born (male 6.4% [0.027-0.11]; female 30.1% [0.22-0.38]); 27.1% [0.25-0.29] US-born (male 8.5% [0.07-0.10]; female 44.9% [0.41-0.49]). | Low risk |
| [66], Escobar B, 2021 | Quantitative, cross-sectional | 2017-2018 Cycle 1: January-May 2017; Cycle 2: | USA | Household | N=4,523 | n=332 | 7.3% | Not specified | NATIVITY: Foreign-born Hispanic (7.3%); US-born Hispanic (11.9%); US-born non-Hispanic white (80.7%).<br><br>NATIVITY/ETHNICITY/COUNTRY OF ORIGIN: | AGE: No average age data available, but all were aged ≥18.<br><br>AGE GROUP by NATIVITY/ETHNI | Both gender. Foreign-born Hispanic (Female 47.99%; Male 52.01%); US-born Hispanic | Not explicitly mentioned, but potentially both Recipient | n/a | n/a | Low risk |

| Study ID, First author, Publication year | Study type/ design | Year(s) of study | Location/country of study | Setting | Total number of participants/ sample size (study completers) | No. of immigrants/first-generation & descendants/ second-generation | Proportion of immigrants/first-generation & descendants/second-generation | Migratory status/ Reason for migration | Country/Region of origin/ Nativity/ Ethnicity(/race) | Age | Gender/ sex | Participant type | Intervention/ programme | Uptake of HPV vaccine | Study risk of bias scores |
|---|---|---|---|---|---|---|---|---|---|---|---|---|---|---|---|
| | | January-May 2018 | | | | | | | Foreign-born Hispanic (Mexican 49.11%; Puerto Rican 5.16%; Cuban 6.21%; Other Hispanic 39.52%); US-born Hispanic (Mexican 48.78%; Puerto Rican 11.26%; Cuban 2.96%; Other Hispanic 36.99%).<br><br>NATIVITY/ETHNICITY/RACE:<br>Foreign-born Hispanic: White (76.78%); Black (5.59%); American Indian (5.74%); Asian (1.71%); Pacific Islander (10.19%).<br>US-born Hispanic: White (79.39%); Black (2.06%); American Indian (8.98%); Asian (3.28%); Pacific Islander (2.1%).<br>Foreign-born non-Hispanic while: White (100%). | CITY:<br>Foreign-born Hispanic: aged 18-34 (n=41; 18.73%); aged 35-49 (n=87; 38.30%); aged 50-64 (n=118; 32.91%); aged 65-74 (n=57; 7.64%); aged 75+ (n=24; 2.42);<br>US-born Hispanic: aged 18-34 (n=117; 36.22%); aged 35-49 (n=157; 34.48%); aged 50-64 (n=141; 20.22%); aged 65-74 (n=74; 5.17%); aged 75+ (n=44; 3.91%);<br>US-born non-Hispanic white: aged 18-34 (n=402; 21.64%); aged 35-49 (n=626; 25.25%); aged 50-64 (n=1,225; 31.21%); aged 65-74 (n=841; 12.96%); aged 75+ (n=519; 8.94%).<br>[X2 p-value, Foreign- vs. US-born Hispanic: <0.001];<br>[X2 p-value, Foreign-born Hispanic vs. non-Hispanic white: <0.001].<br><br>FAMILY AGED 9-27 YEARS (proportion of vaccine-eligible family members).<br>Foreign-born Hispanic: Yes (n=185; 68.75%); No (n=147; 31.25%);<br>US-born Hispanic: Yes (n=283; | (Female 50.90%; Male 49.10%); US-born non-Hispanic white (Female 50.70%; Male 49.30%). | of vaccine and Caregivers with a broad range of age groups (aged ≥18) with data analysed with or without Family member age 9–27 years. | | | |

| Study ID, First author, Publication year | Study type/ design | Year(s) of study | Location/country of study | Setting | Total number of participants/ sample size (study completers) | No. of immigrants/first-generation & descendants/ second-generation | Proportion of immigrants/first-generation & descendants/second-generation | Migratory status/ Reason for migration | Country/Region of origin/ Nativity/ Ethnicity(/race) | Age | Gender/ sex | Participant type | Intervention/ programme | Uptake of HPV vaccine | Study risk of bias scores |
|---|---|---|---|---|---|---|---|---|---|---|---|---|---|---|---|
| | | | | | | | | | | 67.17%); No (n=257; 32.83%); US-born non-Hispanic white: Yes (n=1,186; 45.37%); No (n=2,457; 54.63%). [X2 p-value, Foreign- vs. US-born Hispanic: 0.724]; [X2 p-value, Foreign-born Hispanic vs. non-Hispanic white: <0.001]. | | | | | |
| [67], Barnack-Tavlaris JL, 2016 | Quantitative, cross-sectional | n/a (cannot locate, but used the 2007 CHIS data that has the most recent CHIS database that includes questions about HPV and the HPV vaccine) | USA | Household | N=4,666 (n=1,672 Women, aged 18–27; n=2,994 Mothers, aged 28–65, who had a daughter aged ≥8 [but within the age range for vaccine eligibility] living in their household). | Foreign-born Young adult women, aged 18–27 (n= 425); Foreign-born Mothers, aged 28–65, who had a daughter aged ≥8 [but within the age range for vaccine eligibility] (n= 990). | Foreign-born Young adult women (25.4%); Foreign-born Mothers (33.1%). | Not specified | NATIVITY (Young adult woman, aged 18-27): Foreign-born (25.4%); US-born (74.6%). NATIVITY (Mother of children aged ≥8): Foreign-born (33.1%); US-born (66.9%). ETHNICITY/RACE (Young adult woman, aged 18-27): Foreign-born (White 11.8%; Asian 27.1%; Latina 61.2%); US born (White 62.3%; Asian 9.0%; Latina 28.7%). [p<0.005, White as reference] ETHNICITY/RACE (Mother of children aged ≥8): Foreign-born (White 14.4%; Asian 31.5%; Latina 54.0%); US born (White 84.7%; Asian 2.9%; Latina 12.4%). [p<0.005, White as reference] | AGE: No average age data available.<br><br>AGE GROUP (Young adult woman, aged 18-27): US-born: aged 18-22 (n=607; 48.7%); aged 23-27 (n=640; 51.3%); Foreign-born: aged 18-22 (n=180; 42.4%); aged 23-27 (n=245; 57.6%). [p<0.05] AGE GROUP (Mother of children aged ≥8): US-born: aged 28-42 (n=807; 40.3%); aged 43-65 (n=1197; 59.7%); Foreign-born: aged 28-42 (n=557; 56.3%); aged 43-65 (n=433; 43.7%). [p<0.005] | Female (100%) | Recipient of vaccine (Young adult woman); Parent (Mother of girls aged ≥8) | n/a | n/a | Low risk |
| [68], Budhwani H, 2017 | Quantitative, cross-sectional | 2008 - 2013 (except 2010) data | USA | Household | N=20,040 (adults, aged 18-26) | n=5,962 | 29.75% | Not specified | NATIVITY (Foreign-born) by ETHNICIY/RACE: White 0.08 (95% CI: 0.07-0.09); African American 0.08 (95% CI: 0.07-0.10); Native American 0.14 (95% CI: 0.03-0.25); Asian Indian | AGE: No average age data available. | Both gender: Male 51%; Female 49%. | Potential recipients of vaccine (as early adoption by adults). | n/a | n/a | Low risk |

| Study ID, First author, Publication year | Study type/ design | Year(s) of study | Location/country of study | Setting | Total number of participants/ sample size (study completers) | No. of immigrants/first-generation & descendants/ second-generation | Proportion of immigrants/first-generation & descendants/second-generation | Migratory status/ Reason for migration | Country/Region of origin/ Nativity/ Ethnicity(/race) | Age | Gender/ sex | Participant type | Intervention/ programme | Uptake of HPV vaccine | Study risk of bias scores |
|---|---|---|---|---|---|---|---|---|---|---|---|---|---|---|---|
| | | | | | | | | | 0.72 (95% CI: 0.62-0.82); Chinese 0.55 (95% CI: 0.43-0.66); Filipino 0.36 (95% CI: 0.25-0.46); Other Asian 0.37 (95% CI: 0.29-0.45); All other 0.08 (95% CI: 0.00-0.16). | | | | | | |
| [69], Chu H, 2021 | Quantitative, cross-sectional | October 2017-September 2018 (Evaluation of the interactive education forums). | USA | Community | N=115 (East African mothers with ≥ 1 children aged 11-17) | n=114 (Participating mothers); n=48 (Participants' children). | Participating mothers: 100%; Participants' children: 55.2%. | Not specified | COUNTRY OF ORIGIN (PARTICIPATING MOTHER, East African mothers with ≥ 1children aged 11-17): Somalia (n=92; 80.7%); Ethiopia (n=19; 16.7%); Eritrea (n=3; 2.6%). COUNTRY OF ORIGIN (PARTICIPANT'S CHILDREN): US (49.4%); Somalia (13.8%); Ethiopia (9.2%); Eritrea (1.1%); Other country (31.0%). | AGE: No average age data available. AGE GROUP (Participating mother): aged <30 (n=3; 2.6%); aged 30-39 (n=65; 57.0%); aged 40-49 (n=38; 33.3%); aged 50+ (n=8; 7.0%). AGE GROUP (Participant's child): ≥1 child aged 11-13 (25.2%); ≥1 child aged 14-17 (81.7%). | Participants: Female (100%). Participants' children: Both gender. | Mother (but also data on their child were explored) | Culturally appropriate interactive educational intervention for East African migrant mother | n/a | Low risk |
| [70], Mueller NT, 2012 | Quantitative, cross-sectional | June 2007 - November 2008 | USA | Clinic/Community (safety-net clinics, and their health fairs) | N=1,334 (immigrant Latino aged ≥21 years attending safety-net clinics in 2007–2008) | n=1,334 | 100.0% | Not specified | REGION OF ORIGIN (Immigrant Central and South American Latinos): Central America (64%); South America (27%). | AGE: No average age data available; but all were aged ≥21. AGE GROUP: aged 21-26 (n=188; 14%); aged 27-40 (n=502; 38%); aged >40 (n=644; 48%). | Both gender. Female (55%); Male (45%). | Not mentioned, but potentially both Recipient of vaccine and Parents/caregivers, as those aged ≥21 were included. Note: "Our target population was young and may have been eligible themselves or been parents of | Latino/a migrants aged ≥21 attending safety-net clinics in 2007-2008: Safety-net clinics | n/a | Low risk |

| Study ID, First author, Publication year | Study type/ design | Year(s) of study | Location/country of study | Setting | Total number of participants/ sample size (study completers) | No. of immigrants/first-generation & descendants/ second-generation | Proportion of immigrants/first-generation & descendants/second-generation | Migratory status/ Reason for migration | Country/Region of origin/ Nativity/ Ethnicity(/race) | Age | Gender/ sex | Participant type | Intervention/ programme | Uptake of HPV vaccine | Study risk of bias scores |
|---|---|---|---|---|---|---|---|---|---|---|---|---|---|---|---|
| | | | | | | | | | | | | eligible children." | | | |
| [71], Agénor M, 2018 | Quantitative, cross-sectional | 2011–2015 (data from National Health Interview Survey) | USA | Household | N=15,502 (women, aged 18-31) | n=2,589 | 16.7% | Not specified | NATIVITY: US-born (83.3%); Foreign-born (16.7%).<br>NATIVITY and ETHNICITY/RACE: US-born White (68.2%); US-born Black (17.0%); US-born Latina (12.3%); US-born Asian (2.5%); Foreign-born White (19.2%); Foreign-born Black (0.1%); Foreign-born Latina (47.2%); Foreign-born Asian (24.5%).<br><br>REGION OF ORIGIN and ETHNICITY/RACE and NATIVITY:<br>a) TOTAL - US (86.3%); LAC (6.9%); Europe/Russia/former USSR (1.9%); Africa/Middle East (1.2%); Asia (3.3%); Elsewhere (0.3%).<br>b) US-born White – US (100.0%); US-born Black – US (100.0%); US-born Latina – US (100.0%); US-born Asian – US (100.0%).<br>c) Foreign-born White – LAC (5.0%); Foreign-born White – Europe/Russia/former USSR (63.6%); Foreign-born White – Africa/Middle East (19.1%); Foreign-born White – Asia (2.1%); Foreign-born White – Elsewhere (10.1%).<br>d) Foreign-born Black - LAC (31.6%); Foreign-born Black - Europe/Russia/former USSR (10.7%); Foreign-born Black - Africa/Middle East (55.7%); Foreign-born | AGE GROUP and ETHNICITY/RACE and NATIVITY:<br>AGE GROUP (All): aged 18-26 (53.2%); aged 27-31 (46.8%);<br>AGE GROUP (US-born White): aged 18-26 (53.6%); aged 27-31 (46.4%);<br>AGE GROUP (US-born Black): aged 18-26 (54.7%); aged 27-31 (45.3%);<br>AGE GROUP (US-born Latina): aged 18-26 (60.7%); aged 27-31 (39.4%);<br>AGE GROUP (US-born Asian): aged 18-26 (61.2%); aged 27-31 (38.8%);<br>AGE GROUP (Foreign-born White): aged 18-26 (44.9%); aged 27-31 (55.1%);<br>AGE GROUP (Foreign-born Black): aged 18-26 (44.0%); aged 27-31 (56.0%);<br>AGE GROUP (Foreign-born Latina): aged 18-26 (42.0%); aged 27-31 (58.0%);<br>AGE GROUP (Foreign-born Asia): aged 18-26 (41.8%); aged 27-31 (58.2%). | Female (100%) | Recipient of HPV vaccine | n/a | Self-report: Initiation of HPV vaccination (≥ 1 dose): 33.2% [95%CI: 32.0-34.4] of total participants; White foreign-born 28.4% [22.5-35.1]; Black foreign-born 21.4% [15.6-28.6]; Latina foreign-born 14.0% [12.0-16.3]; Asian foreign-born 14.4% [11.3-18.2]. White US-born 37.6% [35.9-39.3]; Black US-born 30.0% [27.8-32.2]; Latina US-born 32.3% [30.1-34.6]; Asian US-born 40.9% [35.8-46.2]. | Low risk |

| Study ID, First author, Publication year | Study type/ design | Year(s) of study | Location/country of study | Setting | Total number of participants/ sample size (study completers) | No. of immigrants/first-generation & descendants/ second-generation | Proportion of immigrants/first-generation & descendants/second-generation | Migratory status/ Reason for migration | Country/Region of origin/ Nativity/ Ethnicity(/race) | Age | Gender/ sex | Participant type | Intervention/ programme | Uptake of HPV vaccine | Study risk of bias scores |
|---|---|---|---|---|---|---|---|---|---|---|---|---|---|---|---|
| | | | | | | | | | Black - Asia (1.2%); e) Foreign-born Black - Elsewhere (0.8%). f) Foreign-born Latina – LAC (98.2%); Foreign-born Latina – Europe/Russia/former USSR (1.0%); Foreign-born Latina – Africa/Middle East (0/0%); Foreign-born Latina – Asia (0.4%); Foreign-born Latina – Elsewhere (0.5%). g) Foreign-born Asian - LAC (1.2%); Foreign-born Asian - Europe/Russia/former USSR (0.5%); Foreign-born Asian - Africa/Middle East (0.8%); Foreign-born Asian - Asia (96.7%); Foreign-born Asian - Elsewhere (0.9%). | | | | | | |
| [72], Napolitano F, 2018 | Quantitative, cross-sectional | September 2016 - March 2018 (recruited) | Italy | Hospital (waiting room at an Ambulatory centre of the Public General Hospital)<br><br>Intervention/ programme: n/a | N=427 | n=427 | 100.0% | Immigrants and refugees. | REGION OF ORIGIN: Sub-Saharan Africa (64.6%); Eastern Europe (16.6%); South Asia (6.6%); North Africa (4.9%); South America (4.5%); Central Asia (2.8%).<br>COUNTRY OF ORIGIN: Nigeria (17.3%); Senegal (11.5%); Ivory Coast (8.4%). | AGE: Mean 32.4 years (SD=12.8; range 15-73). | Both gender. Female (50.8%); Male (49.2%). | Recipient of vaccine (aged 15 - 26 [although aged 12 - 14 were eligible]); Parents (those who had at least one child aged 12–26). | n/a | Self-report: HPV vaccine receipt: 0.7% of total participant (all foreign-born). | Moderate risk |
| [73], Khodadadi AB, 2022 | Quantitative, cross-sectional | 2013 - 2017 | USA | Community | N=313 Latinx immigrant mothers, who have unvaccinated | n=~313 | ~100% | Not specified | ETHNICITY/COUNTRY OF ORIGIN: Latinx, most from Mexico (no figure is provided). | AGE: Mean 35.00 years (SD=5.59). | Female (100%, mothers) | Parent (mother of unvaccinated girls aged 9-12) | n/a | n/a | Moderate risk |

| Study ID, First author, Publication year | Study type/ design | Year(s) of study | Location/country of study | Setting | Total number of participants/ sample size (study completers) | No. of immigrants/first-generation & descendants/ second-generation | Proportion of immigrants/first-generation & descendants/second-generation | Migratory status/ Reason for migration | Country/Region of origin/ Nativity/ Ethnicity(/race) | Age | Gender/ sex | Participant type | Intervention/ programme | Uptake of HPV vaccine | Study risk of bias scores |
|---|---|---|---|---|---|---|---|---|---|---|---|---|---|---|---|
| | | | | | daughters, aged 9-12. | | | | | | | | | | |
| [74], Becerra MB, 2020 | Quantitative, cross-sectional | n/a (cannot locate, but used data from the 2007 California Health Interview Survey [CHIS] since the main variables to assess health literacy were only available in the 2007 survey) | USA | Household | N=2,050 (Asian adult females, aged 18-65, representing a population size of N = 1,552,710). | n=1,592 | 77.7% | Not specified | NATIVITY: US-born (25.15%); Foreign-born (74.85%). ETHNICITY/RACE (data not disaggregated for migrants): Chinese (31.16%); Filipino (28.21%); South Asian (10.49%); Japanese (5.996%); Korean (10.72%); Vietnamese (13.42%). | AGE: No average age data available. AGE GROUP (data not disaggregated for migrants): aged 18-25 years (19.21%); aged 26-44 years (44.04%); aged 45-65 years (36.74%). | Female (100%) | Recipient of vaccine Note: No questions relating to Parent (mother), although studies age groups would include mothers. | n/a | n/a | Low risk |
| [75], Wemrell M, 2022 | Quantitative, cross-sectional | 2013-2020 (nationwide register data collected) | Sweden | Household | N=311,656 (girls living in Sweden in 2010) | n/a (all 311,656 girls born in Sweden, but both, only one, or neither of their parents were foreign-born) | n/a | Not specified | COUNTRY OF ORIGIN (Girls' Parent): Native Swedish; Mixed; and Migrants (no disaggregated data available). | AGE: Girls aged 10-12, but no detailed information available. | Female (100%, girls) | Recipient of vaccine (= girls, but their parental country of origin was considered in the analyses) | School-based HPV vaccination programme for all girls in the fifth year (aged 10-12) | n/a | Low risk |
| [76], Wiessner C, 2022 | Quantitative, cross-sectional | October 2018 - September 2019 (data collection) | Germany | Household | N=4,955 (population-representative sample from all German federal states) | n=1,120 (first generation migrants, n=636; second generation migrants, n=484). | 22.7% (first generation migrants, 12.9%; second generation migrants, 9.8%). | Not specified | REGION OF ORIGIN/GENERATIONAL STATUS (1st generation migrants): Africa (3.8%); Asia (33.9%); Northern/Western Europe (9.6%); Eastern Europe (39.6%); Southern Europe (13.9%); Northern America (0.6%); Middle/Southern America | AGE (German natives): Mean 48.4 years (SD=15.3); AGE (1st generation migrants): Mean 41.9 years (SD=15.7); AGE (2nd generation migrants): Mean 37.2 years | Both gender. German natives: Female (50.0%); Male (50.0%). 1st generation migrants: Female (49.9%); Male (50.1%). 2nd generation | Recipient of vaccine | n/a | Self-report: HPV vaccine receipt (for those who women who knew preventive measures): 1st generation migrants 15.3% [95% CI: 9.2 - 21.3] vs 2nd generation | Low risk |

| Study ID, First author, Publication year | Study type/ design | Year(s) of study | Location/country of study | Setting | Total number of participants/ sample size (study completers) | No. of immigrants/first-generation & descendants/ second-generation | Proportion of immigrants/first-generation & descendants/second-generation | Migratory status/ Reason for migration | Country/Region of origin/ Nativity/ Ethnicity(/race) | Age | Gender/ sex | Participant type | Intervention/ programme | Uptake of HPV vaccine | Study risk of bias scores |
|---|---|---|---|---|---|---|---|---|---|---|---|---|---|---|---|
| | | | | | | | | | (3.4%); Missing values (1.6%).<br><br>COUNTRY OF ORIGIN: Poland (11.9%), Russia (9.7%); Kazakhstan (6.8%); Turkey (6.3%); Syria (4.7%).<br><br>REGION OF ORIGIN (FATHER)/GENERATAIONAL STATUS (2nd generation migrants): Africa (3.1%); Asia (22.9%); Northern/Western Europe (7.6%); Eastern Europe (19.8%); Southern Europe (16.7%); Northern America (1.0%); Middle/Southern America (1.7%); Germany (27.1%).<br><br>REGION OF ORIGIN (MOTHER)/GENERATIONAL STATUS (2nd generation migrants): Africa (1.7%); Asia (21.7%); Northern/Western Europe (7.4%); Eastern Europe (20.9%); Southern Europe (13.0%); Northern America (0.8%); Middle/Southern America (1.2%); Germany (33.3%).<br><br>TOP 3 REGION OF ORIGIN/GENERATIONAL STATUS (1st generation migrants): REGION OF ORIGIN: Eastern Europe (39.6%); Asia (33.9%); Southern Europe (13.9%). REGION OF ORIGIN (FATHER)/GENERATIONAL STATUS (2nd generation migrants): Germany (27.1%); Asia (22.9%); Eastern Europe (19.8%). | (SD=14.6) [p < 0.001]. | migrants: Female (48.6%); Male (51.4%) [p=0.880]. | | | migrants 35.1% [95% CI: 25.9 - 44.2] vs non-migrants (German native) 47.0% [95% CI: 43.2 - 50.7]. | |

| Study ID, First author, Publication year | Study type/ design | Year(s) of study | Location/country of study | Setting | Total number of participants/ sample size (study completers) | No. of immigrants/first-generation & descendants/ second-generation | Proportion of immigrants/first-generation & descendants/second-generation | Migratory status/ Reason for migration | Country/Region of origin/ Nativity/ Ethnicity(/race) | Age | Gender/ sex | Participant type | Intervention/ programme | Uptake of HPV vaccine | Study risk of bias scores |
|---|---|---|---|---|---|---|---|---|---|---|---|---|---|---|---|
| | | | | | | | | | REGION OF ORIGIN (MOTHER)/GENERATIONAL STATUS (2nd generation migrants): Germany (33.3%); Asia (21.7%); Eastern Europe (20.9%).<br><br>ONE-SIDED/TWO-SIDED MIGRATION BACKGROUND: 1st generation migrants: One-sided (6.9%); Two-sided (93.1%); Missing values (n=112);<br>2nd generation migrants: One-sided (55.3%); Two-sided (44.7%); Missing values (n=16). | | | | | | |
| [77], Bodson J, 2016 | Quantitative, cross-sectional | August - October 2013 (data collection) | USA | Community | N=110 (Hispanic/Latino parents/guardians of adolescents, aged 11-17 [i.e. eligible to receive HPV vaccine]) | n=101; (Parents: n=109) | 92.7% (Parent: 100%) | Not specified | NATIVITY: Foreign-born (91.8%).<br>COUNTRY OF ORIGIN: US (7.3%); Mexico (68.8%); Other* (23.6%).<br>*Other includes Puerto Rico, Brazil, Argentina, Peru, and Portugal.<br>COUNTRY OF ORIGIN (PARENTAL - no mention of mother or father): Mexico (71.8%); Other* (27.3%). | AGE: No average age data available; but the majority 35–50 years old (64.91%).<br>AGE GROUP: aged 16-39 (n=40; 36.4%); aged 40-49 (n=45; 40.9%); aged ≥50 (n=13; 11.8%). | Both gender. Female (83.6%); Male (14.5%). | Parents/guardians (of adolescents, aged 11-17, eligible to receive HPV vaccine). | n/a | n/a | Moderate risk |
| [78], Bhattacharya M, 2021 | Quantitative, cross-sectional | January-May 2017 (Cycle 1 data collection); January-May 2018 (Cycle 2 data collection). | USA | Household | N=2,415 (adults who had ≥ one immediate family member aged 9–27) | n=410 | 17.0% | Not specified | NATIVITY: US-born (83.0%); Foreign-born (17.0%).<br><br>ETHNICITY/RACE:<br>US-born: Non-Hispanic white (64.5%); Non-Hispanic black (10.8%); Hispanic (14.6%); Other/unknown (10.2%);<br>Foreign-born: Non-Hispanic white (14.3%); Non-Hispanic black (10.2%); Hispanic (46.2%); | AGE: No average age data available; but all were aged≥18 with a wide range of age groups. And, adults who had ≥ one immediate family member aged 9–27.<br><br>AGE GROUP (US-born): aged 18-29 (n=292; 27.7%); aged 30-39 (n=207; 10.2%); aged 40-49 (n=457; 25.1%); aged 50-59: (n=575; | Both gender. US-born: Female (53.6%); Male (46.4%). Foreign-born: Female (55.0%); Male (45.0%). [p=0.76] | Not explicitly mentioned; but Recipient of vaccine/Family (adults aged ≥18, with ≥1 immediate family member aged 9–27); | n/a | n/a | Low risk |

| Study ID, First author, Publication year | Study type/ design | Year(s) of study | Location/country of study | Setting | Total number of participants/ sample size (study completers) | No. of immigrants/first-generation & descendants/ second-generation | Proportion of immigrants/first-generation & descendants/second-generation | Migratory status/ Reason for migration | Country/Region of origin/ Nativity/ Ethnicity(/race) | Age | Gender/ sex | Participant type | Intervention/ programme | Uptake of HPV vaccine | Study risk of bias scores |
|---|---|---|---|---|---|---|---|---|---|---|---|---|---|---|---|
| | | | | | | | | | Other/unknown (29.3%) [p<0.01]. | 26.4%); aged 60+ (n=474; 10.5%); AGE GROUP (Foreign-born): aged 18-29 (n=38; 17.7%); aged 30-39 (n=55; 13.8%); aged 40-49 (n=116; 32.8%); aged 50-59 (n=110; 26.2%); aged 60+ (n=91; 9.5%). [p=0.03] | | Recipient of vaccine (Adult respondents' family, aged 9-27). | | | |
| [79], Perez AE, 2018 | Quantitative, cross-sectional | 2011 - 2015 (data collection); February 2017 (data analysis). | USA | Household | N=39,761 (US men aged 18–32, n=15,967; US women aged 18–35, n=23,794) | n=7,379 | 18.6% | Not specified | NATIVITY:<br>Foreign-born (18.6%); US-born (81.4%).<br>Foreign-born, Men (15.13%); US-born, Men (84.87%).<br>Foreign-born, Women (15.44%); US-born, Women (84.56%).<br><br>RACE/ETHNICITY.<br>Total: White (60.6); Black (13.6); Latino/a (17.3); Asian (5.7); Native (0.6); Multiracial (2.1).<br>Foreign-born, Men: White (19.7); Black (8.9); Latino/a (46.2); Asian (24.5); Native (0.3); Multiracial (0.5).<br>US-born, Men: White (70.6); Black (11.6); Latino/a (12.4); Asian (2.6); Native (0.5); Multiracial (2.3).<br>Foreign-born, Women: White (17.9); Black (9.0); Latino/a (48.3); Asian (24.0); Native (0.2); Multiracial (0.6).<br>US-born, Women: White (66.4); Black (16.6); Latino/a (11.5); Asian (2.3); Native (0.7); Multiracial (2.5). | AGE: No average age data available.<br><br>AGE AT TIME OF SURVEY:<br>AGE GROUP (All): aged 18-21 (21.9%); aged 22-26 (35.8%); aged >26 (42.3%).<br>AGE GROUP (Foreign-born, Women): aged 18-21 (12.7%); aged 22-26 (28.1%); aged >26 (59.2%);<br>AGE GROUP (Foreign-born, Men): aged 18-21 (17.8%); aged 22-26 (39.4%); aged >26 (42.8%);<br>AGE GROUP (US-born, Women): aged 18-21 (20.9%); aged 22-26 (32.8%); aged >26 (46.3%);<br>AGE GROUP (US-born, Men): aged 18-21 (26.3%); aged 22-26 (41.0%); aged >26 (32.7%).<br><br>AGE AT HPV VACCINATION ELIGIBILITY.<br>AGE GROUP (All): aged 11-17 (33.0%); | Both gender. Female (59.8%); Male (40.2%). | Recipient of vaccine; Parents/caregivers | n/a | Self-report: Initiation of HPV vaccine (weighted): 18.1% [95% CI: 17.5 - 18.8] of Total participants (Foreign-born 9.5% [8.6 - 10.5] vs. US-born 19.7% [18.9 - 20.4], p<0.0001; Total Men 5.6% [5.0 - 6.2] vs. Total Women 27.1% [26.1 - 28.1]; Foreign-born Men 3.5% [2.7 - 4.5] vs. US-born Men 6.0% [5.4 - 6.7], p<0.0001; Foreign-born Women 13.7% [12.4 - 15.2] vs. US-born Women 29.5% [28.4 - 30.6], p<0.0001). Completion of HPV vaccination (≥3 doses) (weighted): 10.4% [95% CI: 9.9 - 11.0] of Total participants (Foreign-born | Low risk |

| Study ID, First author, Publication year | Study type/ design | Year(s) of study | Location/country of study | Setting | Total number of participants/ sample size (study completers) | No. of immigrants/first-generation & descendants/ second-generation | Proportion of immigrants/first-generation & descendants/second-generation | Migratory status/ Reason for migration | Country/Region of origin/ Nativity/ Ethnicity(/race) | Age | Gender/ sex | Participant type | Intervention/ programme | Uptake of HPV vaccine | Study risk of bias scores |
|---|---|---|---|---|---|---|---|---|---|---|---|---|---|---|---|
| | | | | | | | | | | aged 18-26 (67.0%). AGE GROUP (Foreign-born, Women): aged 11-17 (27.9%); aged 18-26 (72.2%); AGE GROUP (Foreign-born, Men): aged 11-17 (18.9%); aged 18-26 (81.1%); AGE GROUP (US-born, Women): aged 11-17 (40.7%); aged 18-26 (59.3%); AGE GROUP (US-born, Men): aged 11-17 (26.3%); aged 18-26 (73.7%). | | | | 4.5% [3.9- 5.2] vs. US-born 11.5% [10.9 - 12.1], p<0.0001; Total Men 1.6% [1.4 - 1.9] vs. Total Women 16.8% [15.9 - 17.6]; Foreign-born Men 0.8% [0.5 - 1.4] vs. US-born Men 1.8% [1.5 - 2.1], p=0.0001; Foreign-born Women 7.2% [6.1 - 8.4] vs. US-born Women 18.5% [17.5 - 19.4], p<0.0001). | |
| [80], Lee Y-M, 2018 | Quantitative, cross-sectional | n/a | USA | Community | N=74 (Korean American parents - all born in South Korea, first generation, living in USA) | n=74 | 100.0% | Not specified | COUNTRY OF ORIGIN/GENERATIONAL STATUS (first generation): South Korea (100%). | AGE (Parents): Mean 47.16 years (SD=4.07). Note: The figure in the text (Mean 47.21 years (SD=4.01)) not consistent with the figure in Table 1, as shown above.<br><br>AGE (Children): Mean 15.04 years (range 11-18). | Both gender. Female (63.5%); Male (31.1%); Missing (5.4%). | Parents | n/a | Self-report: 72.9% participants (South Korean first generation) had their children initiated and/or completed HPV vaccines. | Low risk |
| [81], Fowler B, 2016 | Quantitative, cross-sectional | July 2013 - June 2014 | USA | Community | N=206 (Latinas, who were overdue for recommended cancer screenings, e.g. cervical, breast, and/or colorectal cancer screenings) | n=199 | 96.6% | Not specified | COUNTRY OF ORIGIN: Mexico (73.79%); Other [either US-born or born in other Latin countries] (24.27%). NATIVITY: Foreign-born (96.6%); US-born (1.5%); No answer (1.9%). | AGE: No average age data available. AGE GROUP: aged ≤26 years (n=10; 4.85%); aged 27-46 years (n=85; 41.26%); aged ≥47 years (n=108; 52.43%). Data are not disaggregated for migrants | Female (100%) | No specific role provided, but judging by age group, it should cover Recipient of vaccine (girls/young women) and (Grand) | n/a | Self-report: HPV vaccine receipt for participants' children 29.6% [37/125] (daughters 35.8% [24/67] vs sons 22.4% [13/58]). | Moderate risk |

| Study ID, First author, Publication year | Study type/ design | Year(s) of study | Location/country of study | Setting | Total number of participants/ sample size (study completers) | No. of immigrants/first-generation & descendants/ second-generation | Proportion of immigrants/first-generation & descendants/second-generation | Migratory status/ Reason for migration | Country/Region of origin/ Nativity/ Ethnicity(/race) | Age | Gender/ sex | Participant type | Intervention/ programme | Uptake of HPV vaccine | Study risk of bias scores |
|---|---|---|---|---|---|---|---|---|---|---|---|---|---|---|---|
| | | | | | | | | | | | | Parent (mother/grandmother). In fact, eligible daughters [N = 67] and sons [N = 58] of participants assessed in Table 5. | | | |
| [82], Tsui J, 2013 | Quantitative, cross-sectional | January - November 2009 (recruited) | USA | Clinic/Household (L.A. County Department of Public Health's Office of Women's Health hotline) | N=468 (mothers/caregivers of girls aged 9-18) | n=410 | 87.6% | Not specified | NATIVITY (MOTHER/CAREGIVER): US-born (12.4%); Foreign-born (87.6%). | AGE (Participating mother/caregiver): No average age data available. AGE (Daughter [adolescent girl] of participating mother/caregiver): Mean 13.9 years. NB: No disaggregated data available for migrants and non-migrants. | Female (100%) | Parent (Mother/caregiver of girls aged 9-18). | Safety-net clinics (Vaccines for Children [VFC] programme which is free-of-charge or low cost for low-income children); Title 317 funding for the provision os vaccines for underinsured populations) | Self-report: HPV vaccine initiation (≥1 dose) was 25.2% of total mothers/caregivers of girls aged 9-18 (Foreign-born 25.9% vs US-born 20.7%; p=0.39). | Low risk |
| [83], McElfish PA, 2021 | Quantitative, cross- | 2014 (data collection) | USA | Household | N=4,880 (young adults, aged 18-26) | n=661 | 13.5% | Not specified | NATIVITY: US-born (85.8%); US territory-born (0.7%); Foreign-born [= neither US-born nor US territory born] (13.5%). | AGE: No average age data available. AGE: 18-26 years | Both gender. Female (50.9%); Male (49.1%). | Recipient of vaccine | n/a | Self-report: Receipt of HPV vaccine (1 dose) (weighted % and 95% CI): Total | Low risk |

| Study ID, First author, Publication year | Study type/ design | Year(s) of study | Location/country of study | Setting | Total number of participants/ sample size (study completers) | No. of immigrants/first-generation & descendants/ second-generation | Proportion of immigrants/first-generation & descendants/second-generation | Migratory status/ Reason for migration | Country/Region of origin/ Nativity/ Ethnicity(/race) | Age | Gender/ sex | Participant type | Intervention/ programme | Uptake of HPV vaccine | Study risk of bias scores |
|---|---|---|---|---|---|---|---|---|---|---|---|---|---|---|---|
| | sectional | | | | | | | | RACE/ETHNICITY: White (53.0%); NHPI (5.7%); Hispanic (19.8%); Black [=non-Hispanic Black/African-American] (13.4%); Asian (6.7%); Other (1.3%). | (n=4,880). | | | | participants 25.8% [23.7 - 28.1] (White 28.3% [25.2 - 31.7%] vs NHPI 24.9% [16.0 - 36.7] vs Hispanic 21.9% [18.7 - 25.5] vs Black 24.7% [20.1 - 29.9] vs Asian 15.4% [11.6 - 20.2] vs Other 33.0% [17.2 - 53.8], p=0.003; **Foreign-born 14.3%** [10.9 - 18.5] vs US-born 27.4% [25.0 - 29.9] vs US territory-born 27.7% [10.3 - 56.0], p<0.001; Males 10.1% [8.4 - 12.0] vs Females 41.8% [38.3 - 45.3], p<0.001). | |
| [84], Gelman A, 2013 | Quantitative, cross-sectional | July 2008 - June 2010 (data collection) | USA | Household | N=2,168 (females, aged 15-24) | n=149 | 6.9% | Not specified | NATIVITY: US-born (93.1%); Foreign-born (6.9%).<br><br>ETHNICITY/RACE: White (51.2% [weighted %: 63.6%]); Hispanic US-born (18.7% [weighted %: 13.8%]); Hispanic foreign-born (6.9% [weighted %: 5.3%]); African-American (23.2% [weighted %: 17.2%]).<br>Note: weighted % - weighted to reflect US female household population. | AGE. No average age data available.<br><br>AGE GROUP (All): aged 15-18 (n=872; 40.2%); aged 19-24 (n=1,296; 59.8%).<br>Note: No disaggregated data available for migrants.<br><br>AGE GROUP by ETHNICITY (White): aged 15-18 (35.2%); aged 19-24 (64.8%);<br>AGE GROUP by ETHNICITY (African-American): aged 15-18 (40.3%); aged 19-24 (59.7%); | Female (100%) | Recipient of vaccine | All girls and boys aged 11-12 (3 dose series) and Catch-up aged 13-26 for females (and aged 13-21 for males) | Self-report: Initiation of HPV vaccine (≥1 dose): Total participants 28.4% (White 33.1% vs US-born Hispanic 24.2% vs Foreign-born Hispanic 16.2% vs African American 18.2%). | Low risk |

| Study ID, First author, Publication year | Study type/ design | Year(s) of study | Location/country of study | Setting | Total number of participants/ sample size (study completers) | No. of immigrants/first-generation & descendants/ second-generation | Proportion of immigrants/first-generation & descendants/second-generation | Migratory status/ Reason for migration | Country/Region of origin/ Nativity/ Ethnicity(/race) | Age | Gender/ sex | Participant type | Intervention/ programme | Uptake of HPV vaccine | Study risk of bias scores |
|---|---|---|---|---|---|---|---|---|---|---|---|---|---|---|---|
| | | | | | | | | | | AGE GROUP by NATIVITY/ETHNICITY (Hispanic US-born): aged 15-18 (46.8%); aged 19-24 (53.2%); AGE GROUP by NATIVITY/ETHNICITY (Hispanic foreign-born): aged 15-18 (26.9%); aged 19-24 (73.1%); [p=0.001] | | | | | |
| [85], Minhat HS, 2013 | Quantitative, cross-sectional | April - June 2010 | Malaysia | Community | N=271 (Iranian women aged 18 -60 year olds who were living in Malaysia) | n=271 | 100.0% | Not specified | COUNTRY OF ORIGIN: Iran (100%). | AGE: Mean 35.2 years. AGE GROUP: aged <30 (n=114; 42.1%); aged ≥30 (n=157; 57.9%). | Female (100%) | Not mentioned, but likely to be Caregivers (because Mean age was 35.2 years), but potentially also Recipient of vaccine. | n/a | n/a | Low risk |
| [86], Bastani R, 2011 | Quantitative, cross-sectional | January - November 2009 (data collection through telephone interviews) | USA | Clinic/ Household (L.A. County Department of Public Health's Office of Women's Health hotline) | N=490 (Mothers/caregivers of vaccine-eligible girls aged 9–18 [using the Los Angeles County Department of Public Health, Office of Women's Health service referral hotline]: Mothers of vaccine-eligible girls [85%]; Caregivers of vaccine eligible girls, including grandmothers, | n=432 | 88.2% | Not specified | NATIVITY: US-born (11.8%); Foreign-born (88.2%).<br><br>ETHNICITY/ RACE: Latina (52.0%); Chinese (20.0%); Korean (13.5%); African American (7.8%); Other, including other Asian, Non-Latino White, and Multirace (6.7%). | AGE (Participating mother/caregiver): Mean 43.8 years (no SD data available). No disaggregated data for migrants and non-migrants. . AGE (Adolescent girl of participating mother/caregiver): Mean 13.0 years (no SD data available). No disaggregated data for migrants and non-migrants. . | Female (100%) | Parent (mother of vaccine-eligible girls, aged 9-18: 85%); Caregivers (of vaccine eligible girls, e.g. grandmothers, step-mothers, aunts, or older sisters: 15%). Note: Participants are users | Vaccines for Children [VFC] programme (free-of-charge or low cost for low-income girls) | n/a | Moderate risk |

| Study ID, First author, Publication year | Study type/ design | Year(s) of study | Location/country of study | Setting | Total number of participants/ sample size (study completers) | No. of immigrants/first-generation & descendants/ second-generation | Proportion of immigrants/first-generation & descendants/second-generation | Migratory status/ Reason for migration | Country/Region of origin/ Nativity/ Ethnicity(/race) | Age | Gender/ sex | Participant type | Intervention/ programme | Uptake of HPV vaccine | Study risk of bias scores |
|---|---|---|---|---|---|---|---|---|---|---|---|---|---|---|---|
| | | | | | step-mothers, aunts, or older sisters [15%]) | | | | | | | of the Los Angeles County Department of Public Health, Office of Women's Health service referral hotline | | | |
| [87], Sundaram MK, 2021 | Quantitative, cross-sectional | n/a | UAE | University | N=269 (private university female expatriate students aged 18-26, irrespective of their educational specialisation) | n=269 | 100.0% | University expatriate students | RACE/ETHNICITY: Majority of the students (Asian sub-continent ancestry predominantly from India, Pakistan, Bangladesh); other (African continent or neighbouring Arab nationals). | AGE: No average age data available. AGE: range 18-26. | Female (100%) | Receipt of vaccine | n/a | Self-report: Uptake of HPV vaccine: 5.2% total participants (all were migrants); Completion of HPV vaccine (3 doses) among initiators: 64.3% among initiators. | Moderate risk |
| [88], Khodadadi AB, 2020 | Quantitative, cross-sectional | 2013-2016 | USA | Community | N=317 (Latina immigrant mothers of daughters, aged 9-12) | n=317 | 100% | Not specified | RACE/ETHNICITY: Latina | AGE: Mean 35.13 years (SD=5.81). | Female (100%) | Parent (mother) | n/a | n/a | Low risk |
| [89], Guo Y, 2023 | Quantitative, cross-sectional | 2007 (data collection) | USA | Household | N=784 (first-generation immigrant parents with adolescent girls aged 11-12) | First generation: n=784 | First generation: 100% | Not specified | RACE/ETHNICITY: Non-Hispanic White (30.3%); African (6.0%); Hispanic (39.8%); Asian (26.1%). | AGE: Mean 48 years (SD=16.0). | Both gender. Female (56.8%); Male (43.2%). | Parents (Mother 56.8%; Father 43.2%) | HPV vaccination for children aged 11–12 | n/a | Low risk |
| [90], Ratnasamy P, 2022 | Quantitative, cross-sectional | 2018 | USA | Household | N=17,004 | Foreign-born includes 287 People of Indian Ancestry (POIA) and others. | Foreign-born includes 1.69% People of Indian Ancestry (POIA) and others. | Not specified | RACE/ETHNICITY: POIA (n=287; 1.69%); White; Black; Other Asian; and Those of other/mixed ancestry (no break down figures are available except POIA) | AGE: No average age data available. AGE GROUP: aged 18–25; aged 26–45; and aged 46–64 (no break down figures available). | Both gender. | Recipient of vaccine | n/a | Self-report: Initiation of HPV vaccine: US-born POIA 41.78% vs Foreign-born POIA 4.85%, p=0.0186; Completion of HPV vaccine among initiators (≥1 dose): US- | Moderate risk |

| Study ID, First author, Publication year | Study type/ design | Year(s) of study | Location/country of study | Setting | Total number of participants/ sample size (study completers) | No. of immigrants/first-generation & descendants/ second-generation | Proportion of immigrants/first-generation & descendants/second-generation | Migratory status/ Reason for migration | Country/Region of origin/ Nativity/ Ethnicity(/race) | Age | Gender/ sex | Participant type | Intervention/ programme | Uptake of HPV vaccine | Study risk of bias scores |
|---|---|---|---|---|---|---|---|---|---|---|---|---|---|---|---|
| | | | | | | | | | | | | | | born POIA 27.05% vs Foreign-born POIA 1.36%, p=0.0819. RACE - Initiation of HPV vaccine: POIA 8.18% vs White 12.16%, Black 14.70%, Other Asian 16.07%, Those of other/mixed ancestry 12.41%, p= 0.003. Completion of HPV vaccine amongst initiators (≥1 dose): POIA 3.17% vs White 4.27%, Black 3.51%, Other Asian 4.31%, Those of other/mixed ancestry 5.04%, p= 0.465. Initiation of HPV vaccine in Those born in USA (vs Foreign-born): POIA, p = 0.018; White, p = 0.006; Black, p = 0.029; Other Asian, p = 0.020; Those of other/mixed ancestry, p = 0.019. | |
| [91], Groene EA, 2022 | Quantitative, cross-sectional | January 2015–July 2018 | USA | Household | N=170,256 (adolescents) | n=31,713 | 18.6% | Not specified | NATIVITY by GENDER: US-born (Female 40.1%; Male 39.8%); Non-US born (Female 9.7%; Male 10.4%) NATIVITY (Parent): Both parents non-US-born (n=19852; 11.7%); Both parents US-born (n=126912; 74.5%); Only 1 US-born | AGE: Mean ~12.4 years (no SD data available). | Both gender. GENDER: Female (n=84,092; 49.4%); Male (n=86,164; 50.6%). | Recipient of vaccine (adolescent) | Recommended routine HPV vaccination. Medicaid and Minneso | The State-wide Minnesota Immunization Information Connection (MIIC) (routine vaccination data from ~ 91% of healthcare | Moderate risk |

| Study ID, First author, Publication year | Study type/ design | Year(s) of study | Location/country of study | Setting | Total number of participants/ sample size (study completers) | No. of immigrants/first-generation & descendants/ second-generation | Proportion of immigrants/first-generation & descendants/second-generation | Migratory status/ Reason for migration | Country/Region of origin/ Nativity/ Ethnicity(/race) | Age | Gender/ sex | Participant type | Intervention/ programme | Uptake of HPV vaccine | Study risk of bias scores |
|---|---|---|---|---|---|---|---|---|---|---|---|---|---|---|---|
| | | | | | | | | | parent (n=11861; 7.0%); Missing both parents' nativity (n=11631; 6.8%). REGION OF ORIGIN (Mother): Africa (n=6382; 3.7%); Asia (n=7821; 4.6%); Eastern Europe (n=662; 0.4%); Latin Americana and the Caribbean (n=9324; 5.5%); Oceania and other (n=1475; 0.9%); US (n=142867; 83.9%); Western Europe/Canada (n=1271; 0.7%); Missing (n=454; 0.3%) | | | | ta Vaccines for Children programme (free-of-charge) | providers from the Minnesota Vaccines for Children programme): Missed opportunity is defined as "receipt of any vaccine between ages 11 and 14 without receipt of the HPV vaccine". No missed opportunities (= Receipt of HPV vaccine during routine vaccination visit for "routine adolescent vaccines such as meningococcal, Tdap, or other catch-up or booster vaccines"): Total participants 69.7% (Female 50.6%; Male 49.4%, p<0.001); (Both parents non-US-born 12.2%; Both parents US-born 73.2%; Only 1 US-born parent 7.2%; Missing both parents' nativity 7.4%, p<0.001); (Maternal region of origin: Africa 3.5%; Asia 4.8%; Eastern Europe 0.3%; LAC 6.3%; Oceania and other | |

| Study ID, First author, Publication year | Study type/ design | Year(s) of study | Location/country of study | Setting | Total number of participants/ sample size (study completers) | No. of immigrants/first-generation & descendants/ second-generation | Proportion of immigrants/first-generation & descendants/second-generation | Migratory status/ Reason for migration | Country/Region of origin/ Nativity/ Ethnicity(/race) | Age | Gender/ sex | Participant type | Intervention/ programme | Uptake of HPV vaccine | Study risk of bias scores |
|---|---|---|---|---|---|---|---|---|---|---|---|---|---|---|---|
| | | | | | | | | | | | | | | 0.9%; USA 83.2%; Western Europe/Canada 0.7%; Missing 0.3%, p<0.001). | |
| [92], Alsulami FT, 2023 | Quantitative, cross-sectional | April 2022 (Recruitment) | USA | College | N=376 (foreign-born college students) | n=376 | 100.0% | International college students (32.7%). | RACE/ETHNICITY: White (22.3%); Black (3.5%); Hispanic or Latino (42.3%); Asian (19.7%); Other (12.2%). | AGE: Mean 24.42 years (SD=5.49).<br><br>AGE GROUP (Young adult students): aged 18–26 (n=245; 65.2%); AGE GROUP (Middle-aged students): aged 27–45 (n=131; 34.8%). | Both gender. Female (67%); Male (33%). | Recipient of vaccine | Routine catch-up HPV vaccination for those aged 13-26, and recommended the HPV vaccination for those aged 27-45 (Advisory Committee on Immunization Practices [ACIP]) | Self-report: Receipt of HPV vaccine (≥1 dose): Total participants (Foreign-born) 63%; Female 71.4% vs Male 46%, p<0.001; White 63.1% vs Black 69.2% vs Hispanic/Latino 72.3% vs Asian 55.4% vs Other ethnicity 41.3%, p=0.002; International student 53.7% vs Non-international student 67.6%, p=0.009; Young adult students aged 18-26 72.2% vs Middle-aged students aged 27-45 45.8, p<0.001; Living in the US 10 years or less 56.9% vs More than 10 years 71.5%, p=0.004. | Low risk |
| [93], Berman RS, 2017 | Quantitative, Cross-sectional | 2011–2013 | USA | Clinic (Refugee Health Assessment Program [RHAP] clinics<br><br>) | N=2,269 | n=2,269 | 100.0% | Refugee | RACE/ETHNICITY: Black (n=803; 35%); Asian (n=765; 34%); White (n=679; 30%); Other (n=12; 0.5%); Unknown (n=10; 0.4%).<br><br>COUNTRY OF ORIGIN: Iraq (n=571; 25%); Bhutan (n=538; 24%); Somalia (n=255; 11%). | AGE GROUP: aged 9-12 (n=431; 19%); aged 13-26 (n=1,838; 81%) | GENDER/SEX: Female (n=1,040; 46%); Male (1,228; 54%). | Recipient of vaccine | Refugee Health Assessment Programme (RHAP) at the Massachusetts Department of | Massachusetts Department of Public Health (MDPH)'s web-based surveillance data through Massachusetts Virtual Epidemiologic Network (MAVEN) | Moderate risk |

| Study ID, First author, Publication year | Study type/ design | Year(s) of study | Location/country of study | Setting | Total number of participants/ sample size (study completers) | No. of immigrants/first-generation & descendants/ second-generation | Proportion of immigrants/first-generation & descendants/second-generation | Migratory status/ Reason for migration | Country/Region of origin/ Nativity/ Ethnicity(/race) | Age | Gender/ sex | Participant type | Intervention/ programme | Uptake of HPV vaccine | Study risk of bias scores |
|---|---|---|---|---|---|---|---|---|---|---|---|---|---|---|---|
| | | | | | | | | | REGION OF ORIGIN: Sub-Saharan Africa excluding Somalia (n=481; 21%); East Asia and Pacific (n=214; 9%); Western Hemisphere (n=93; 4%); Europe and Eurasia (n=64; 3%); Near East, excluding Iraq (n=24; 1%); South and Central Asia, excluding Bhutan (n=29; 1%). | | | | Public Health (MDPH) | : Receipt of HPV vaccine (1 dose): 68% refugees aged 13 -17 vs 45% US adolescents aged 13 -17.<br>56% all refugees aged 9-26 at pre/post-arrival. Sub-Saharan Africa excluding Somalia 63% (reference) vs Somalia 51% [p=0.001], Near East excluding Iraq 29% [p=0.002], Iraq 61% [p=0.62], South and Central Asia, excluding Bhutan 41% [p=0.03], Bhutan 56% [p=0.03], East Asia and Pacific 56% [p=0.07], Europe and Eurasia 23% [p<0.001], Western Hemisphere 48% [p=0.009]. | |
| [94], Chen AC-C. 2023 | Quantitative, Cross-sectional (pre-intervention) | Unknown.<br><br>Note: The intervention was developed through web-based DST workshops between July 2021 - January 2022. | USA | Community | N=164 | N=164: Korean American mothers (n=50) and Vietnamese American mothers (n=114), who had ≥1 children aged 9-14 who had not received HPV vaccine | 100.0% | Not specified | Mothers' COUNTRY OF ORIGIN: South Korea and Vietnam (first generation immigrants) | Korean American mothers: Mean 42.8 years (SD=4.8).<br><br>Vietnamese American mothers: Mean 41.5 years (SD=5.4).<br><br>Children of Korean American mothers: Mean 10.6 years (SD=1.8). | Adult women (Mother, aged 18 and over) - All: Female (100%)<br><br>Children (aged 9-14 years old) - All: Male n=70 (43.2%); Female n=92 (56.8%).<br><br>Children of Korean | Mothers (only gender data provided for their children) | Digital storytelling (DST). | n/a | Moderate risk |

| Study ID, First author, Publication year | Study type/ design | Year(s) of study | Location/country of study | Setting | Total number of participants/ sample size (study completers) | No. of immigrants/first-generation & descendants/ second-generation | Proportion of immigrants/first-generation & descendants/second-generation | Migratory status/ Reason for migration | Country/Region of origin/ Nativity/ Ethnicity(/race) | Age | Gender/ sex | Participant type | Intervention/ programme | Uptake of HPV vaccine | Study risk of bias scores |
|---|---|---|---|---|---|---|---|---|---|---|---|---|---|---|---|
| | | | | | | | | | | Children of Vietnamese American mothers: Mean 12.7 years (SD=1).<br><br>Children - All: Mean 12.1 years (SD=1.6). | American mothers: Male n=19 (39.6%); Female n=29 (60.4%).<br><br>Children of Vietnamese American mothers: Male n=70 (43.2%); Female n=92 (56.8%). | | | | |
| [95], Han SH, 2023 | Quantitative, Cross-sectional | June - August 2021 | USA | College | N=133 (Asian immigrant college undergraduate students) | n=133 | 100.0% | Immigrant College Students | COUNTRY OF ORIGIN: China/Taiwan (34.6%); South Korea (25.6%); Tibet (9.0%); Nepal (7.5%); India (5.3%); The Philippines (5.3%); and other (12.7%). | AGE: Mean 25.12 years (SD=5.38, range 18–44) | Women (69.9%); Men (30.1%) | Recipient of vaccine | n/a | Self-reported: Receipt of HPV vaccine (≥1 dose): 47.4 % of participants (all were migrants). | Moderate risk |
| [96], Lee HY, 2023 | Quantitative, Cross-sectional | September 2019 - February 2020 | USA | Community | N=278 | n=278 | 100.0% | Not specified | COUNTRY OF ORIGIN: South Korea | AGE GROUP: aged 18-49 (n= 197; 71.1%); aged ≥50 (n=80; 28.9%). | Female (n=144; 52.0%); Male (n=133; 48.0%). | Potentially recipient of vaccine and parents | n/a | n/a | Moderate risk |
| [97], Lee J-Y, 2024 | Quantitative, Cross-sectional | 17 February 2021 - 14 August 2021 | Republic of Korea | Community | N=262 (immigrant women with children [son or daughter aged 9 – 19]) | N=262.<br><br>Immigrant child: Yes (n=59/262; 22.5%); No (n=203/262; 77.5%) | 100.0% | Not specified | RACE/ETHNICITY: Chinese, Vietnamese, Korean-Chinese women with children.<br><br>Chinese (n=114/262; 43.5%); Vietnamese (n=105/262; 40.1%); Korean-Chinese (n=43/262; 16.4%). | Mothers' AGE: not available.<br><br>Child's AGE: Mean 12.05 years (SD = 2.63) | Immigrant women: Female (100%)<br><br>Children of immigrant women: Both sexes | Mother (but also their child's data, recipient of vaccine, were included) | Free-of-charge HPV vaccination for girls aged 12; mandatory | Self-report: Completion of HPV vaccine among children (fully vaccinated against HPV): 10.7% | High risk |
| [98], Liu CCH, 2024 | Quantitative, Cross-sectional | 2016 – 2019 | USA | University | N=575 (international students/scholars and their families in 2016 [n=118], 2017 [n=163], 2018 [n=193], 2019 [n = 101]) | n=575 | 100.0% | International university students /scholars and their families | RACE/ETHNICITY: 2016 (White n=7, 5.9%; Black n=4, 3.3%; Asian n=105, 88.9%; Missing n=2, 1.6%); 2017 (White n=6, 3.6%; Black n=2, 1.2%; Asian n=64, 39.2%; Missing n=91, 55.8%); 2018 (White n=12, 6.2%; Black n=2, 1%; Asian n=136, 7.4%; Missing n=43, 22.2%); 2019 (White n=3, | AGE (Mean ± SD): 2016 (26.2 ± 12.68); 2017 (23.48 ± 10.29); 2018 (23.83 ± 11.77); 2019 (27.02 ± 13.96). | Female (n=331; 57.6%); Male (n=244; 42.4%). | Recipient of vaccine | Evidence-based university vaccination initiative (a pre-registration event; | Self-report: Receipt of HPV vaccine: Year 2016, 33% vs Year 2017, 36.8% vs Year 2018, 37.8% vs Year 2019, 21.7% [p=0.023]. Aged 0-17, 21.5% vs aged 18-25, 71.8% vs aged 26- | Moderate risk |

| Study ID, First author, Publication year | Study type/ design | Year(s) of study | Location/country of study | Setting | Total number of participants/ sample size (study completers) | No. of immigrants/first-generation & descendants/ second-generation | Proportion of immigrants/first-generation & descendants/second-generation | Migratory status/ Reason for migration | Country/Region of origin/ Nativity/ Ethnicity(/race) | Age | Gender/ sex | Participant type | Intervention/ programme | Uptake of HPV vaccine | Study risk of bias scores |
|---|---|---|---|---|---|---|---|---|---|---|---|---|---|---|---|
| | | | | | | | | | 2.9%; Black n=1, 0.9%; Asian n=19, 18.8%; Missing n=78, 77.2%). | | | | vaccine recommendations from healthcare professionals with a bilingual interpreter; campus-based marketing strategies; reminders via social media; free/affordable vaccines) | 40, 7.7% vs aged 41-70, 0% [p<0.001]. Sex: Male 29.5% vs Female 37.8% [p=0.039]. Race: Asian 36.8% vs White 17.9% vs Black 0% [p=0.034]. | |
| [99], Mullassery D, 2024 | Quantitative, Cross-sectional | Unknown | USA | Community | N=173 (immigrant and first-generation Asian Indian parents of children aged 9-16) | n=173 (Immigrant and first-generation) | 100.0% (Immigrant and first-generation) | Not specified | RACE/ETHNICITY: Asian Indian | AGE: Mean 43.68 years (SD = 5.68) | GENDER OF PARENTS: Female (n=113/173; 65.3%); Male (n=60/173; 34.7%).<br><br>GENDER OF THE CHILD: Female (n=97/173; 56.06%); Male (n=76/173; 43.93%). | Parents (of children aged 9-16) | n/a | Self-report: Receipt of HPV vaccine: 20.2% | Low risk |
| [100], Karki I, 2022 | Quantitative, Cross- | November 2019 - January 2020 | USA | College | N=588 | n = 100 (international students - both | 17.0% | International college students | RACE/ETHNICITY (All): White (n=411; 69.9%); Black/African American (n=23; 3.9%); American Indian/Alaska (n=17; 2.9%); | AGE (All): Mean 21.43 years (SD = 3.12); Age (International students): Mean | GENDER/SEX (All): Female (n=450; 76.5%); Male (n=138; | Recipient of vaccine | n/a | Self-report: Receipt of HPV vaccine: 28% (28/100) of international | High risk |

| Study ID, First author, Publication year | Study type/ design | Year(s) of study | Location/country of study | Setting | Total number of participants/ sample size (study completers) | No. of immigrants/first-generation & descendants/ second-generation | Proportion of immigrants/first-generation & descendants/second-generation | Migratory status/ Reason for migration | Country/Region of origin/ Nativity/ Ethnicity(/race) | Age | Gender/ sex | Participant type | Intervention/ programme | Uptake of HPV vaccine | Study risk of bias scores |
|---|---|---|---|---|---|---|---|---|---|---|---|---|---|---|---|
| | sectional | | | | | undergraduate and graduate) | | | Asian (n=85; 14.5%); Other (n=52; 8.8%).<br><br>RACE/ETHNICITY (International students): White (n=36; 36%); Black/African American (n=10; 10%); American Indian/Alaska (n=0; 0%); Asian (n=39; 39%); Other (n=15; 15%).<br><br>RACE/ETHNICITY (US students): White (n=375; 76.8%); Black/African American (n=13; 2.7%); American Indian/Alaska (n=17; 3.5%); Asian (n=46; 9.4%); Other (n=37; 7.6%).<br><br>COUNTRY OF ORIGIN: 42 countries in total - India (n=10; 10%), China (n=6; 6%), Iran (n=6; 6%), South Korea (n=6; 6%), Pakistan (n=4; 4%), Nigeria (n=4; 4%), Nepal (n=4; 4%), and Brazil (n=4; 4%). | 23.46 years (SD = 3.55); Age (US students): Mean 21.01 years (SD = 2.84). | 23.5%); Gender/Sex (International students): Female (n=65; 65%); Male (n=35; 35%); Gender/Sex (US students): Female (n=385; 78.9%); Male (n=103; 21.1%). | | | students; Completion of HPV vaccine (3 doses) among initiators: International students 85.71% vs 65.38% US students. Place of vaccination among international students: 32.14% in the USA vs 67.85% in their home country. | |
| [101], Amdisen L, 2018 | Quantitative, cohort | February 2017 (data extraction) | Denmark | Household | N=161,528 (girls born between 1999-2003, living in Denmark in February 2017 and residing in Denmark between their 12th -13th birthday: 1999-2000 cohort [n=65,548; 40.6%]; 2001-2003 cohort [n=95,980; 59.4%]) | Descendants and Immigrants: n=6,751 (1999-2000 cohort); n=10,270 (2001-2003 cohort) | Descendants and Immigrants: 10.3% (1999-2000 cohort); 10.7% (2001-2003 cohort) | Not specified | NATIVITY/GENERATIONAL STATUS (Study participant: girl): 1999-2000 cohort: Danish (89.7%); Descendant (7.9%); Immigrant (2.4%). 2001-2003 cohort: Danish (89.3%); Descendant (8.3%); Immigrant (2.4%).<br><br>COUNTRY OF ORIGIN/ETHNICITY/RACE: Not available. | AGE (Mother) by STUDY PARTICIPANT COHORT (Girl): 1999-2000 cohort: aged 12-19 (1.3%); aged 20-24 (11.8%); aged 25-29 (33.9%); aged 30-34 (35.5%); aged 35-39 (14.9%); aged 40-60 (2.6%). 2001-2003 cohort: aged 12-19 (1.2%); aged 20-24 (10.5%); aged 25-29 (32.8%); aged 30-34 (35.8%); aged 35-39 (16.7%); aged 40-60 (3.0%).<br><br>BIRTH ORDER: | Female (100%, girls) | Parent (Mother); Recipient of vaccine (Daughter) | n/a | n/a | Moderate risk |

| Study ID, First author, Publication year | Study type/ design | Year(s) of study | Location/country of study | Setting | Total number of participants/ sample size (study completers) | No. of immigrants/first-generation & descendants/ second-generation | Proportion of immigrants/first-generation & descendants/second-generation | Migratory status/ Reason for migration | Country/Region of origin/ Nativity/ Ethnicity(/race) | Age | Gender/ sex | Participant type | Intervention/ programme | Uptake of HPV vaccine | Study risk of bias scores |
|---|---|---|---|---|---|---|---|---|---|---|---|---|---|---|---|
| | | | | | | | | | | 1999-2000 cohort: 1st (42.4%); 2nd (37.6%); 3rd (14.5%); 4th (3.9%); >5th (1.6%). 2001-2003 cohort: 1st (43.1%); 2nd (37.2%); 3rd (14.3%); 4th (3.8%); >5th (1.6%). | | | | | |
| [102], Hansen BT, 2015 | Quantitative, cohort | 2009-2011 | Norway | Household | N=90,842 (Norwegian girls born 1997–1999 [aged 12-13], eligible for routine school-based HPV vaccination in 2009–2011, and their registered mother and father).<br><br>n=90,540 girl-mother pairs included in vaccination initiation analysis; n=88,565 girl-father pairs included in vaccination initiation analysis. n=70,870 girl-mother pairs included in vaccinatio9n completion analysis; n=69,306 girl-father pairs included in vaccination | n=12,541 | 14.0% | Not specified | NATIVITY by Parent gender (Mother) and by HPV vaccination (Initiation): Norway-born (n=76256; 85.0%); Foreign-born (n=13430; 15.0%). NATIVITY by Parent gender (Mother) and by HPV vaccination (Completion): Norway-born (n=59862; 85.2%); Foreign-born (n=10423; 14.8%). NATIVITY by Parent gender (Father) and by HPV vaccination (Initiation): Norway-born (n=74745; 86.5%); Foreign-born (n=11652; 13.5%). NATIVITY by Parent gender (Father) and by HPV vaccination (Completion): Norway-born (n=58706; 86.7%); Foreign-bon (n=8969; 13.3%).<br><br>COUNTRY/REGION OF ORIGIN by Parent gender (Mother) and by HPV vaccination (Initiation): Norway (n=76256; 85.0%); Old EU [Countries who joined the EU before 2004]/EEA/EFTA, USA, Canada, Australia, NZ (n=3309; 3.7%); Newer EU & other Europe [Countries who joined the EU in 2004/2007 & European | AGE: No age average data available.<br><br>AGE GROUP by Parent gender and by HPV vaccination (Mother, HPV vaccination initiation): aged <35 (n=9,854; 10.9%); aged 35-39 (n=26,012; 28.7%); aged 40-44 (n=32,872; 36.3%); aged 45-49 (n=16,993; 18.8%); aged ≥50 (n=9,854; 5.3%). AGE GROUP by Parent gender and by HPV vaccination (Mother, HPV vaccination completion): aged <35 (n=7,888; 11.1%); aged 35-39 (n=20,767; 29.3%); aged 40-44 (n=25,995; 36.7%); aged 45-49 (n=12,831; 18.1%); aged ≥50 (n=3,389; 4.8%). AGE GROUP by Parent gender and by HPV vaccination (Father, HPV | Girls: Female (100%). Girls' mothers and fathers: Both gender. Note: N=90,540 girl-mother pairs included in initiation analysis; N=88,565 girl-father pairs included in initiation analysis. N=70,870 girl-mother pairs included in completion analysis; N=69,306 girl-father pairs included in completion analysis. | Recipient of vaccine (girl); Parents (mothers & fathers). | Publicly funded, school-based routine HPV vaccination programme | Norwegian immunisation registry (SYSVAK): Initiation of HPV vaccine: Maternal country of birth – Norway (host) 78.5% vs Old EU/EEA/EFTA, USA, Canada, Australia, NZ 73.5% vs Newer EU & other Europe 77.8% vs Africa 72.2% vs Asia 82.5% vs Central and South America 73.1%; Paternal country of birth – Norway (host) 78.5% vs Old EU/EEA/EFTA, USA, Canada, Australia, NZ 73.8% vs Newer EU & other Europe 78.2% vs Africa 71.6% vs Asia 81.4% vs Central and South America 72.6%. Completion of HPV vaccine among initiators: Maternal country of birth – Norway | Low risk |

| Study ID, First author, Publication year | Study type/ design | Year(s) of study | Location/country of study | Setting | Total number of participants/ sample size (study completers) | No. of immigrants/first-generation & descendants/ second-generation | Proportion of immigrants/first-generation & descendants/second-generation | Migratory status/ Reason for migration | Country/Region of origin/ Nativity/ Ethnicity(/race) | Age | Gender/ sex | Participant type | Intervention/ programme | Uptake of HPV vaccine | Study risk of bias scores |
|---|---|---|---|---|---|---|---|---|---|---|---|---|---|---|---|
| | | | | | completion analysis. | | | | countries outside of EU/EEA/EFTA] (n=2752; 3.1%); Africa (n=1793; 2.0%); Asia (n=5104; 5.7%); Central and South America (n=472; 0.5%). COUNTRY/REGION OF ORIGIN by Parent gender (Mother) and by HPV vaccination (Completion): Norway (n=59862; 85.2%); Old EU [Countries who joined the EU before 2004]/EEA/EFTA, USA, Canada, Australia, NZ (n=2433; 3.5%); Newer EU & other Europe [Countries who joined the EU in 2004/2007 & European countries outside of EU/EEA/EFTA] (n=2140; 3.0%); Africa (n=1194; 1.8%); Asia (n=4211; 6.0%); Central and South America (n=345; 0.5%). COUNTRY/REGION OF ORIGIN by Parent gender (Father) and by HPV vaccination (Initiation): Norway (n=74745; 86.5%); Old EU [Countries who joined the EU before 2004]/EEA/EFTA, USA, Canada, Australia, NZ (n=3316; 3.8%); Newer EU & other Europe [Countries who joined the EU in 2004/2007 & European countries outside of EU/EEA/EFTA] (n=2165; 2.5%); Africa (n=1662; 1.9%); Asia (n=5104; 5.7%); Central and South America (n=4170; 4.8%). COUNTRY/REGION OF ORIGIN by Parent gender (Father) and by HPV vaccination (Completion): Norway (n=58706; 86.7%); | vaccination initiation): aged <35 (n=3,344; 3.8%); aged 35-39 (n=16,810; 19.0%); aged 40-44 (n=31,070; 35.1%); aged 45-49 (n=22,678; 25.6%); aged ≥50 (n=14,663; 16.6%). AGE GROUP by Parent gender and by HPV vaccination (Father, HPV vaccination completion): aged <35 (n=2,675; 3.9%); aged 35-39 (n=13,456; 19.4%); aged 40-44 (n=24,584; 35.5%); aged 45-49 (n=17,703; 25.5%); aged ≥50 (n=10,888; 15.7%). DAUGHTER'S YEAR OF BIRTH by Parent gender (Mother) and by HPV vaccination (Initiation): 1997 (n=30,420; 33.6%); 1998 (n=29,904; 33.0%); 1999 (n=30,316; 33.4%). DAUGHTER'S YEAR OF BIRTH by Parent gender (Mother) and by HPV vaccination (Completion): 1997 (n=21,895; 30.9%); 1998 (n=24,045; 33.9%); 1999 (n=24,930; 35.2%). DAUGHTER'S YEAR OF BIRTH | | | | (host) 95.4% vs Old EU/EEA/EFTA, USA, Canada, Australia, NZ 95.2% vs Newer EU & other Europe 94.9% vs Africa 95.4% vs Asia 94.9% vs Central and South America 93.3%; Paternal country of birth – Norway (host) 95.3% vs Old EU/EEA/EFTA, USA, Canada, Australia, NZ 95.3% vs Newer EU & other Europe 95.8% vs Africa 95.7% vs Asia 95.2% vs Central and South America 94.3%. Initiation of HPV vaccine: Mothers – Vietnam 90.6% [86.6-93.5], Thailand 87.8% [83.5-91.1], Kosovo 87.3% [82.1-91.2], Afghanistan 85.8% [80.0-90.2], Pakistan 84.3% [80.7-87.3], Turkey 84.1% [78.9-88.2], Iran 81.6% [75.2-86.7], Iraq 81.1% [76.8-84.8], Philippines 80.6% [75.4-84.9], Poland 80.5% [76.4-84.1], Sri Lanka 80.5% [75.5- | |

| Study ID, First author, Publication year | Study type/ design | Year(s) of study | Location/country of study | Setting | Total number of participants/ sample size (study completers) | No. of immigrants/first-generation & descendants/ second-generation | Proportion of immigrants/first-generation & descendants/second-generation | Migratory status/ Reason for migration | Country/Region of origin/ Nativity/ Ethnicity(/race) | Age | Gender/ sex | Participant type | Intervention/ programme | Uptake of HPV vaccine | Study risk of bias scores |
|---|---|---|---|---|---|---|---|---|---|---|---|---|---|---|---|
| | | | | | | | | | Old EU [Countries who joined the EU before 2004]/EEA/EFTA, USA, Canada, Australia, NZ (n=2447; 3.6%); Newer EU & other Europe [Countries who joined the EU in 2004/2007 & European countries outside of EU/EEA/EFTA] (n=1692; 2.5%); Africa (n=1190; 1.8%); Asia (n=3394; 5.0%); Central and South America (n=246; 0.4%). | by Parent gender (Father) and by HPV vaccination (Initiation): 1997 (n=29788; 33.6%); 1998 (n=29242; 33.0%); 1999 (n=29535; 33.3%). DAUGHTER'S YEAR OF BIRTH by Parent gender (Father) and by HPV vaccination (Completion): 1997 (n=21414; 30.9%); 1998 (n=23508; 33.9%); 1999 (n=24384; 35.2%). | | | | 84.7], Sweden 79.1% [75.4-82.4], Norway 78.5% [78.1-78.9], Denmark 77.8% [72.7-82.1], Bosnia-Herzegovina 75.1% [67.5-81.4], US 74.9% [69.2-80.0], Costa Rica 71.1% [64.6-76.8], UK 69.7% [61.7-76.6], Germany 65.1% [58.6-71.0], Somalia 63.9% [59.3-68.3]; Fathers - Vietnam 90.2% [86.1-93.5], Thailand 73.7% [41.8-93.8] , Kosovo 85.6% [80.6-89.8], Afghanistan 88.7% [83.1-93.0], Pakistan 81.7% [78.1-85.0], Turkey 80.5% [75.3-85.0], Iran 77.6% [72.0-82.6], Iraq 81.4% 77.2-85.2], Philippines 84.9% [73.9-92.6], Poland 79.8% [74.7-84.4], Sri Lanka 79.5% [74.7-83.8], Sweden 76.0% [71.7-79.8], Norway 78.5% [78.2-78.9], Denmark 77.8% [72.4-82.7], Bosnia-Herzegovina 75.1% [67.6-81.6], US 70.4% | |

| Study ID, First author, Publication year | Study type/ design | Year(s) of study | Location/country of study | Setting | Total number of participants/ sample size (study completers) | No. of immigrants/first-generation & descendants/ second-generation | Proportion of immigrants/first-generation & descendants/second-generation | Migratory status/ Reason for migration | Country/Region of origin/ Nativity/ Ethnicity(/race) | Age | Gender/ sex | Participant type | Intervention/ programme | Uptake of HPV vaccine | Study risk of bias scores |
|---|---|---|---|---|---|---|---|---|---|---|---|---|---|---|---|
| | | | | | | | | | | | | | | [64.4-76.0], Costa Rica 69.7% [59.3-78.8], UK 76.6% [70.8-81.8], Germany 76.1% [70.1-81.4], Somalia 62.9% [57.7-67.9]. | |
| [103], Tung IL, 2016 | Quantitative, cohort | September 2011 - December 2014 (recruitment surveys); 2015 (additional supplementary surveys) | Australia | Community | N=417 (adolescent girls and young women, both unvaccinated and vaccinated [recruitment/supplementary surveys]) | n=53 (Study participant - adolescent girls and young women).<br><br>(Participant's parents: n=152). | 12.7% (Study participant - adolescent girls and young women).<br><br>(Participant's parents: 37.0%). | Not specified | NATIVITY (Participating adolescent girls and young women): Australia-born (87.2%); Foreign-born (12.8%).<br><br>Note: "Other countries of birth include New Zealand, China, Fiji, Finland, Germany, Hong Kong, India, Indonesia, Japan, Kenya, Malaysia, Singapore, Serbia, South Africa, Sri Lanka, Sweden, United Kingdom, US, Vietnam".<br><br>NATIVITY (Parent): Both Australia-born (63.0%); One parent born overseas (19.5%); Both parents born overseas (17.5%).<br>Note: "Overseas countries correspond to New Zealand, Bangladesh, Brunei, Canada, Chile, China, Egypt, Fiji, Finland, France, Germany, Hong Kong, India, Indonesia, Israel, Italy, Kenya, Latvia, Lebanon, Malaysia, Malta, Mauritius, Netherlands, Pakistan, Papua New Guinea, Philippines, Poland, Republic of Malawi, Sweden, Singapore, Spain, South Africa, Sri Lanka, Tanzania, United Kingdom, US, Vietnam, Zimbabwe." | NB. No disaggregated data available for migrants and non-migrants.<br><br>AGE: Median 24 years (interquartile range [IQR]: 22–25). Note: "Overall, the majority of women (77%) were under 18 years of age at the commencement of the program".<br><br>AGE AT PROGRAMME COMMENCEMENT: aged 11-17 (n=320; 76.7%); aged 18-21 (n=97; 23.3%).<br>BIRTH COHORT 1996-1994 (n=46; 11.0%); 1993-1992 (n=95; 22.8%); 1991-1990 (n=124; 29.7%); 1989 (n=108; 25.9%); 1987-1986 (n=44; 10.7%). | Female (100%) | Recipient of vaccine (adolescent girls and young women) | Free-of-charge, school-based National HPV Vaccination Programme for all girls aged 12-18 between 2007-2009 and Catch-up in the community for women aged ≤26 | National HPV Vaccination Program Register (NHVPR) and Self-report: Fully vaccinated: 81% of total participants. The 1st vaccine dose receipt amongst the vaccinated: 68% at school vs 32% at a GP. Fully vaccinated: Country of birth: Australia (host) 92.2% vs Other 7.8%, p<0.001; Parental country of birth: Both Australian born 66.5% vs One parent born overseas 20.4% vs Both parents born overseas 13.2%, p<0.001; Childhood vaccinations: Incomplete 3.0% vs Complete 97.0%, p<0.001. | Moderate risk |

| Study ID, First author, Publication year | Study type/ design | Year(s) of study | Location/country of study | Setting | Total number of participants/ sample size (study completers) | No. of immigrants/first-generation & descendants/ second-generation | Proportion of immigrants/first-generation & descendants/second-generation | Migratory status/ Reason for migration | Country/Region of origin/ Nativity/ Ethnicity(/race) | Age | Gender/ sex | Participant type | Intervention/ programme | Uptake of HPV vaccine | Study risk of bias scores |
|---|---|---|---|---|---|---|---|---|---|---|---|---|---|---|---|
| [104], Wang J, 2019 | Quantitative, cohort | 2007-2014 | Sweden | Household | N=689,676 (girls born between 1990-2003 [with 537,151 biological/ foster mothers and 526,106 fathers = 1,063,257]) | n=137,227 (Girls with foreign-born mother: 139,938; Girls with foreign-born father: 134,516). | 19.9% (Girls with foreign-born mother: 20.3%; Girls with foreign-born father: 19.5%). | Not specified | NATIVITY by Parent gender (Mother): Sweden-born (n=534,479; 77.5%); Other (n=139,938; 20.3%); Missing (n=15,259; 2.2%). NATIVITY by Parent gender (Father): Sweden-born (n=526,509; 76.3%); Other (n=134,516; 19.5%); Missing (n=28,651; 4.2%).<br><br>COUNTRY OF ORIGIN/ETHNICITY/RACE: Not available. | AGE: No average age data available. Notes: i)Subsidised vaccination for girls aged 13-17, implemented from second half of 2007 to 2012. ii) Catch-up (free of charge) vaccination for girls birth year 1993-1998, up to aged 17, implemented between 2012 and 2015. iii)School-based (free of charge) vaccination for girls with birth year 1999-2001, 2000-2002, 2001-2003, implemented between 2012 and 2015. | Girls: Female (100%, recipient of vaccine); Girl's Parents: Both gender (537,151 biological/ foster mothers and 526,106 fathers). | Recipient of vaccine (girl); Parents (biological /foster mother and father). | Subsidised opportunistic; Free-of-charge catch-up outside-school; and Free-of-charge school-based HPV vaccination | n/a | Low risk |
| [105], de Casadevante VF, 2016 | Quantitative, cohort | August 2012-December 2013 | Denmark | Household | N=274,154 (women, aged 19-28 years) | Whole cohort: n=76,135. Cohort after excluding the immigrant population living in Denmark for <6 years: 49,362. Cohort who could have initiated the free-of-charge HPVV programme (230,032 women born between 1985 - 1992): 71,061. | Whole cohort: 27.8%. Cohort after excluding the immigrant population living in Denmark for <6 years: 20.0%. Cohort who could have initiated the free-of-charge HPVV programme (230,032 women born between 1985 - 1992): 30.9%. | Not specified | NATIVITY: Denmark-born women both parents Denmark-born (n=197415; 72.0%); Denmark-born women one parent Denmark-born (n=16780; 6.1%); Descendants [Denmark-born women of foreign descent] (n=9860; 3.6%); Migrants (n=49495; 18.1%).<br><br>COUNTRY OF ORIGIN (Young woman participant): Native: Denmark-born (n=214195/274195; 78.1%); Descendants: Turkey Descendant (n=3469; 1.3%); Pakistan Descendant (n=1014; 0.4%); Lebanon Descendant (n=956; 0.3%); Migrants: Norway (n=2572; | AGE: No average age data available; but age range 19-28 years. | Female (100%) | Receipt of vaccine | i)Temporary free-of-charge HPV vaccine catch-up programme (27 August 2012 – 31 December 2013) for all female Danish citizens born in 1985 – 1992; ii) Self- | Danish Vaccination Registry (DDV): Initiation of HPV vaccine (1st dose) - During the cost-free programme: Denmark-born with 2 parents Denmark-born, 79.74% vs Denmark-born with 1 parent Denmark-born 75.67% vs Descendants 65.87% vs Immigrants 58.87%; Under self-payment: Denmark-born with 2 parents | Low risk |

| Study ID, First author, Publication year | Study type/ design | Year(s) of study | Location/country of study | Setting | Total number of participants/ sample size (study completers) | No. of immigrants/first-generation & descendants/ second-generation | Proportion of immigrants/first-generation & descendants/second-generation | Migratory status/ Reason for migration | Country/Region of origin/ Nativity/ Ethnicity(/race) | Age | Gender/ sex | Participant type | Intervention/ programme | Uptake of HPV vaccine | Study risk of bias scores |
|---|---|---|---|---|---|---|---|---|---|---|---|---|---|---|---|
| | | | | | | | | | 0.9%); Sweden (n=1655; 0.6%); Faroe Islands (n=1349; 0.5%); Greenland (n=1020; 0.4%); Germany (n=2394; 0.9%); Spain (n=895; 0.3%); France (n=832; 0.3%); USA (n=104; 0.0%); Poland (n=2817; 1.0%); Romania (n=2372; 0.9%); Lithuania (n=1624; 0.6%); Bosnia-Herzegovina (n=1394; 0.5%); Latvia (n=814; 0.3%); Ukraine (n=1183; 0.4%); The Philippines (n=2554; 0.9%); China (n=1358; 0.5%); South Korea (n=980; 0.4%); India (n=851; 0.3%); Iraq (n=1692; 0.6%); Afghanistan (n=1220; 0.4%); Turkey (n=1076; 0.4%); Somalia (n=959; 0.3%).<br>NB. The figures provided here are about the whole cohort, before excluding migrants living in Denmark for <6 years. | | | | payment, voluntary HPV vaccine programme (2006 – 26 August 2012; 2014 - ) for women born in 1985-1992 | Denmark-born, 19.77% vs Denmark-born with 1 parent Denmark-born 16.82% vs Descendants 5.54% vs Immigrants 7.16% (Immigrant population residing in Denmark for < 6 years excluded). Norway 67.7%, Sweden 63.9%, Faroe Islands 75.4%, Greenland 63.1%, Germany 48.9%, Spain 44.5%, France 40.6%, USA 51.0%, Poland 42.2%, Romania 40.5%, Lithuania 41.0%, Bosnia-Herzegovina 69.2%, Latvia 39.2%, Ukraine 45.3%, The Philippines 57.3%, China 56.4%, South Korea 80.1%, India 79.2%, Iraq 56.8%, Afghanistan 68.3%, Turkey 48.9%, Somalia 34.9% (Immigrant population residing in Denmark for < 6 years excluded). | |
| [106], Møller SP, 2018 | Quantitative, cohort | October 2008 - May 2012 | Denmark | Household | N=22,848 (n=3,264 refugee girls; n=19,584 | n=3,264 | 14.3% | Legally refugee (but girls | NATIVITY: Refugee girls (14.3%) (who gained residency permits in Denmark between 1 January | AGE: No average age data available.<br><br>AGE AT OFFER OF | Female (100%) | Receipt of vaccine (girl - ordinary | Ordinary HPV immunisation | Danish National Health Service Register: n/a | Low risk |

| Study ID, First author, Publication year | Study type/ design | Year(s) of study | Location/country of study | Setting | Total number of participants/ sample size (study completers) | No. of immigrants/first-generation & descendants/ second-generation | Proportion of immigrants/first-generation & descendants/second-generation | Migratory status/ Reason for migration | Country/Region of origin/ Nativity/ Ethnicity(/race) | Age | Gender/ sex | Participant type | Intervention/ programme | Uptake of HPV vaccine | Study risk of bias scores |
|---|---|---|---|---|---|---|---|---|---|---|---|---|---|---|---|
| | | | | | Danish-born girls) | | | living in asylum centres [eligible for free-of-charge vaccination] were excluded). Ordinary programme: Asylum seekers (85%); Quota-refugees (15%). Catch-up: Asylum seekers (89%); Quota-refugees (11%). | 1994 -31 December 2010; and matched on age and sex with Danish-born girls (85.7%).<br><br>NATIVITY (Refugee girls): Ordinary programme (39.7%), Catch-up (60.3%). NATIVITY (Danish-born girls): Ordinary programme (39.7%), Catch-up (60.3%).<br><br>REGION OF ORIGIN (refugee participants ONLY):<br>a) Ordinary programme: Afghanistan (18%); Asia (9%); Bosnia-Herzegovina (5%); Former Yugoslavia (13%); Iraq (22%); MENA (5%); Somalia (9%); Stateless Palestinian (10%); Sub-Sahara (7%); Eastern Europe (2%).<br>b)Catch-up: Afghanistan (13%); Asia (8%); Bosnia-Herzegovina (19%); Former Yugoslavia (11%); Iraq (18%); MENA (2%); Somalia (8%); Stateless Palestinian (15%); Sub-Sahara (4%); Eastern Europe (1%). | IMMUNISATION:<br>a) Ordinary programme (Refugee): aged 12 (n=1,295; 100%); aged 13 (n/a); aged 14 (n/a); aged 15 (n/a);<br>Ordinary programme (Danish-born): aged 12 (n=7,770; 100%); aged 13 (n/a); aged 14 (n/a); aged 15 (n/a).<br><br>b)Catch-up (Refugee): aged 12 (n=113; 6%); aged 13 (n=651; 33%); aged 14 (n=716; 36%); aged 15 (n=489; 25%);<br>Catch-up (Danish-born): aged 12 (n=829; 7%); aged 13 (n=3,781; 32%); aged 14 (n=4,102; 35%); aged 15 (n=3,102; 26%). | | and catch-up programmes) | and Catch-up programmes | | |
| [107], Hertzum-Larsen R, 2020 | Quantitative, cohort | 2009-2015/17 | Denmark | Household | N=260,251 | n=26,539 | 10.2% | Not specified | NATIVITY/GENERATION: Native Danish-born girls (89.8%); Descendant girls (7.9%); Immigrant girls (2.3%).<br><br>REGION OF ORIGIN (mother's country of origin if known; otherwise father's):<br>Natives: Denmark (100%).<br>Descendants: North Africa and Western Asia (50%); Mid and Eastern Asia (20%); Eastern Europe | AGE: No average age data available (but, all girls born in 1996–2003 identified). | Female (100%) | Recipient of vaccine (girl, but some parental information gathered). | Free-of-charge, routine HPV vaccination for girls aged 12 since 1 January 2009, which is clinic-based (mainly | National Health Service Register: HPV vaccination (≥1 dose within 2 years after the eligibility for routine vaccination).<br>Girls born in 1996-2003 - Denmark 85% (95% CI: 85-86) vs Descendants 84% (95% CI: 83-84) vs Immigrants | Low risk |

| Study ID, First author, Publication year | Study type/ design | Year(s) of study | Location/country of study | Setting | Total number of participants/ sample size (study completers) | No. of immigrants/first-generation & descendants/ second-generation | Proportion of immigrants/first-generation & descendants/second-generation | Migratory status/ Reason for migration | Country/Region of origin/ Nativity/ Ethnicity(/race) | Age | Gender/ sex | Participant type | Intervention/ programme | Uptake of HPV vaccine | Study risk of bias scores |
|---|---|---|---|---|---|---|---|---|---|---|---|---|---|---|---|
| | | | | | | | | | (14%); Sub-Saharan Africa (10%); Western countries (5%); South and Central America (0%). Immigrants: Mid and Eastern Asia (24%); Eastern Europe (24%); North Africa and Western Asia (22%); Western countries (16%); Sub-Saharan Africa (12%); South and Central America (2%). | | | | provided by GPs) | 74% (95% CI: 73-75). Descendants – Mid and Eastern Asia 87% (86-88), Vietnam 91% (89-93), Sri Lanka 90% (88-92), Afghanistan 86% (83-89), Pakistan 82% (80-84), Other countries in Mid and Eastern Asia 87% (84-89), North Africa and Western Asia 85% (84-86), Turkey 87% (86-88), Lebanon 85% (83-86), Iran 85% (82-88), Iraq 84% (82-86), Syria 82% (77-86), Morocco 79% (76-82), Other countries in North Africa and Western Asia 85% (82-87), Eastern Europe 82% (81-84), Bosnia and Herzegovina 84% (82-86), Macedonia 85% (81-89), Yugoslavia 84% (81-86), Poland 77% (71-83), Other countries in Eastern Europe 78% (74-81), South and Central America 77% (67-85), Sub-Saharan Africa 76% (74-78), Somalia 73% (71-75), Other countries in Sub-Saharan Africa 87% (83-89), | |

| Study ID, First author, Publication year | Study type/ design | Year(s) of study | Location/country of study | Setting | Total number of participants/ sample size (study completers) | No. of immigrants/first-generation & descendants/ second-generation | Proportion of immigrants/first-generation & descendants/second-generation | Migratory status/ Reason for migration | Country/Region of origin/ Nativity/ Ethnicity(/race) | Age | Gender/ sex | Participant type | Intervention/ programme | Uptake of HPV vaccine | Study risk of bias scores |
|---|---|---|---|---|---|---|---|---|---|---|---|---|---|---|---|
| | | | | | | | | | | | | | | Western countries 75% (72-77), Germany 68% (62-74), Other Western countries 77% (74-80). Immigrants – Mid and Eastern Asia 78% (76-80), Afghanistan 82% (78-85), Thailand 79% (74-84), Other countries in Mid and Eastern Asia 76% (72-79), North Africa and Western Asia 81% (79-83), Iraq 82% (79-85), Other countries in North Africa and Western Asia 79% (75-82), Eastern Europe 67% (64-69), Poland 65% (60-69), Other countries in Eastern Europe 68% (65-71), South and Central America 69% (59-78), Sub-Saharan Africa 72% (69-75), Somalia 68% (63-73), Other countries in Sub-Saharan Africa 75% (71-79), Western countries 72% (69-75), Iceland 80% (74-85), Germany 65% (60-70), Other Western countries 74% (69-78). Girls birth cohort 1996-2000: Region of origin – | |

| Study ID, First author, Publication year | Study type/ design | Year(s) of study | Location/country of study | Setting | Total number of participants/ sample size (study completers) | No. of immigrants/first-generation & descendants/ second-generation | Proportion of immigrants/first-generation & descendants/second-generation | Migratory status/ Reason for migration | Country/Region of origin/ Nativity/ Ethnicity(/race) | Age | Gender/ sex | Participant type | Intervention/ programme | Uptake of HPV vaccine | Study risk of bias scores |
|---|---|---|---|---|---|---|---|---|---|---|---|---|---|---|---|
| | | | | | | | | | | | | | | Denmark: 93% vs Descendants 89% vs Immigrants 79%. Descendants – Mid and Eastern Asia 92%, North Africa and Western Asia 90%, Eastern Europe 88%, South and Central America 94%, Sub-Saharan Africa 82%, Western countries 81%. Immigrants -– Mid and Eastern Asia 83%, North Africa and Western Asia 83%, Eastern Europe 74%, South and Central America 76%, Sub-Saharan Africa 75%, Western countries 78%.<br>Girls birth cohort 2001-2003: Region of origin – Denmark: 73% vs Descendants 76% vs Immigrants 63%. Descendants – Mid and Eastern Asia 80%, North Africa and Western Asia 77%, Eastern Europe 73%, South and Central America 56%, Sub-Saharan Africa 70%, Western countries 65%. Immigrants -– Mid and Eastern Asia 67%, North | |

| Study ID, First author, Publication year | Study type/ design | Year(s) of study | Location/country of study | Setting | Total number of participants/ sample size (study completers) | No. of immigrants/first-generation & descendants/ second-generation | Proportion of immigrants/first-generation & descendants/second-generation | Migratory status/ Reason for migration | Country/Region of origin/ Nativity/ Ethnicity(/race) | Age | Gender/ sex | Participant type | Intervention/ programme | Uptake of HPV vaccine | Study risk of bias scores |
|---|---|---|---|---|---|---|---|---|---|---|---|---|---|---|---|
| | | | | | | | | | | | | | | Africa and Western Asia 73%, Eastern Europe 56%, South and Central America 55%, Sub-Saharan Africa 65%, Western countries 62%. | |
| [108], Dong L, 2021 | Quantitative, cohort | October 2006–June 2018 overall. Note: October 2006–June 2018 (vaccinated opportunistically, self-paid, at one's own initiative), and November 2016–June 2018 (vaccinated free-of-charge in a catch-up vaccination programme). | Norway | Household | N=839,251 (Female residents born in Norway between 1975-1996 and resident any time Oct 2006-Jun 2018: Opportunistic vaccination cohort [n=839,251]; Catch-up vaccination cohort [n=201,326]) | HPV vaccination initiation, Opportunistic: n= 289,089; HPV vaccination initiation, Catch-up: n= 48,933. | HPV vaccination initiation, Opportunistic: 34.4%; HPV vaccination initiation, Catch-up: 24.3%. | Not specified | NB. Data on population of interest's Country of origin is not available, but only their parent's data. HPV VACCINATION INITIATION – OPPORTUNISTIC SETTING. OVERALL: 839,251 NATIVITY (Parent): Two Norwegian-born parents (63.5%); Two immigrant parents (29.6%); One foreign-born and one Norwegian-born parent (4.8%). HPV VACCINATION COMPLETION – OPPORTUNISTIC SETTING. OVERALL: 15,211 NATIVITY (Parent): Two Norwegian-born parents (83.7%); Two immigrant parents (7.7%); One foreign-born and one Norwegian-born parent (8.6%). HPV VACCINATION INITIATION – CATCH-UP SETTING. OVERALL: 201,326 NATIVITY (Parent): Two Norwegian-born parents (72.7%); Two immigrant parents (17.5%); One foreign-born and one Norwegian-born parent (6.9%). | AGE: No average data available (range: 10-43 years). Note: "We identified all women in the National Registry who were born between 1975 and 1996 and were resident in Norway at any time during October 2006 to June 2018". AGE AT INITIATION OF HPV VACCINATION (Opportunistic): Median 21.3 years (interquartile range: 16.4–27.6 years). AGE AT INITIATION OF HPV VACCINATION (Catch-up): Median 23.3 years (interquartile range: 21.8–24.8 years). | Female (100%, adolescent and adult) | Receipt of vaccine (but also parental data were explored) | Self-paid opportunistic and Free-of-charge catch-up HPV vaccination programmes | Norwegian Immunization Registry (SYSVAK) and Prescription Registry (for dates of vaccination and prescription for each dose, respectively): Initiation of HP vaccine (≥1 dose of any HPV vaccine e.g. 2v, 4v, 9v for opportunistic vaccination vs ≥1 dose of 2v HPV vaccine for catch-up vaccination): Opportunistic vaccination - 2.2%; Catch-up vaccination (during the first 20 months) - 46.2%. Completion of HPV vaccine (≥3 doses of the same HPV vaccine within 1 year) among initiators: Opportunistic vaccination – 72.1%; Catch-up vaccination 73.0%. | Moderate risk |

| Study ID, First author, Publication year | Study type/ design | Year(s) of study | Location/country of study | Setting | Total number of participants/ sample size (study completers) | No. of immigrants/first-generation & descendants/ second-generation | Proportion of immigrants/first-generation & descendants/second-generation | Migratory status/ Reason for migration | Country/Region of origin/ Nativity/ Ethnicity(/race) | Age | Gender/ sex | Participant type | Intervention/ programme | Uptake of HPV vaccine | Study risk of bias scores |
|---|---|---|---|---|---|---|---|---|---|---|---|---|---|---|---|
| | | | | | | | | | HPV VACCINATION COMPLETION – CATCH-UP SETTING.<br>OVERALL: 63,440<br>NATIVITY (Parent): Two Norwegian-born parents (86.2%); Two immigrant parents (7.3%); One foreign-born and one Norwegian-born parent (6.4%). | | | | | Initiation of HPV vaccine: Opportunistic vaccination – Overall 2.2% (Year of birth: 1975-1978: 0.4% vs 1979-1981: 0.8% vs 1982-1984: 1.2% vs 1985-1987: 1.6% vs 1988-1990: 3.6% vs 1991-1993: 3.2% vs 1994-1996: 5.5%; Parental country of birth: Two Norwegian-born parents: 2.9% vs Two immigrant parents: 0.6% vs One foreign-born and one Norwegian-born parent: 3.8%).<br><br>Completion of HPV vaccine among initiators: Opportunistic vaccination – Overall 72.1% (Year of birth: 1975-1978: 56.5% vs 1979-1981: 65.9% vs 1982-1984: 69.7% vs 1985-1987: 62.6% vs 1988-1990: 64.7% vs 1991-1993: 75.7% vs 1994-1996: 79.3%; Parental country of birth: Two Norwegian-born parents: 72.9% vs Two immigrant parents: 65.2% vs One foreign-born | |

| Study ID, First author, Publication year | Study type/ design | Year(s) of study | Location/country of study | Setting | Total number of participants/ sample size (study completers) | No. of immigrants/first-generation & descendants/ second-generation | Proportion of immigrants/first-generation & descendants/second-generation | Migratory status/ Reason for migration | Country/Region of origin/ Nativity/ Ethnicity(/race) | Age | Gender/ sex | Participant type | Intervention/ programme | Uptake of HPV vaccine | Study risk of bias scores |
|---|---|---|---|---|---|---|---|---|---|---|---|---|---|---|---|
| | | | | | | | | | | | | | | and one Norwegian-born parent: 71.0%).<br><br>Initiation of HPV vaccine: Catch-up vaccination – Overall 46.2% (Year of birth: 1991-1992: 43.2% vs 1993-1994: 46.6% vs 1995-1996: 48.9%; Parental country of birth: Two Norwegian-born parents: 53.9% vs Two immigrant parents: 21.1% vs One foreign-born and one Norwegian-born parent: 45.1%).<br><br>Completion of HPV vaccine among initiators: Catch-up vaccination – Overall 73.0% (Year of birth: 1991-1992: 73.4% vs 1993-1994: 72.9% vs 1995-1996: 72.7%; Parental country of birth: Two Norwegian-born parents: 73.5% vs Two immigrant parents: 68.3% vs One foreign-born and one Norwegian-born parent: 71.6%). | |

| Study ID, First author, Publication year | Study type/ design | Year(s) of study | Location/country of study | Setting | Total number of participants/ sample size (study completers) | No. of immigrants/first-generation & descendants/ second-generation | Proportion of immigrants/first-generation & descendants/second-generation | Migratory status/ Reason for migration | Country/Region of origin/ Nativity/ Ethnicity(/race) | Age | Gender/ sex | Participant type | Intervention/ programme | Uptake of HPV vaccine | Study risk of bias scores |
|---|---|---|---|---|---|---|---|---|---|---|---|---|---|---|---|
| [109], Suppli CH, 2018 | Quantitative, cohort | 15 May 2014–14 May 2015 | Denmark | Household | N=9,692 (girls) | n=1,386 | 14.3% | Not specified | REGION OF ORIGIN (Mother): Danish-born (85.7%); Western immigrant (2.4%); Non-Western immigrant (11.9%). | AGE (Girl as main target group): 14 years old (in the study period: between 15 May 2014 - 14 May 2015). Note: No disaggregated data available for migrants and non-migrants. AGE GROUP (Mother): aged <25 (n=1,531; 15.8%); aged 25-34 (n=6,580; 67.9%); aged >35 (n=1,580; 16.3%). Note: No disaggregated data available for migrants and non-migrants. | Female (100%, girls) | Recipient of vaccine (girls aged 14); Their mothers' data are also explored as predictors for responding to a personalised reminder and subsequent uptake of HPV vaccine. | A personalised reminder for HPV and/or MMR vaccination | n/a | Low risk |
| [110], Slåttelid Schreiber SM, 2015 | Quantitative, cohort | 2009-2012 | Denmark | Household | N=127,088 (two birth cohorts: girls born in 1996 or 1997) | n=11,779 (Immigrants n=8,972; n=2,807). | 9.3% (Immigrants + Descendants: 11,779/127,088). | Not specified | NATIVITY/GENERATION (Girl): Denmark-born (90.5%); Descendants (7.1%); Immigrants (2.2%); Unknown (0.2%). | AGE: No average age data available. | Girls: Female (100%). Girl's guardians: Both gender | Receipt of vaccine; Parent (Guardians) | Free-of-charge, HPV vaccination (national HPV childhood vaccination programme), provided by GPs | Danish National Health Insurance Service Register and National Prescription Registry (outside the programme, e.g. all pharmacy-purchased HPV vaccines): Initiation of HPV vaccine (≥1 dose): Overall 92.8% of girls (Birth cohort 1996: 92.1% vs 1997: 93.4%) (Danish 93.7% vs Descendant 88.6% vs Immigrant 84.4% vs Unknown 2.5%); Completion of HPV vaccine (3 doses) among initiators: Overall 83.6% (Birth | Low risk |

| Study ID, First author, Publication year | Study type/ design | Year(s) of study | Location/country of study | Setting | Total number of participants/ sample size (study completers) | No. of immigrants/first-generation & descendants/ second-generation | Proportion of immigrants/first-generation & descendants/second-generation | Migratory status/ Reason for migration | Country/Region of origin/ Nativity/ Ethnicity(/race) | Age | Gender/ sex | Participant type | Intervention/ programme | Uptake of HPV vaccine | Study risk of bias scores |
|---|---|---|---|---|---|---|---|---|---|---|---|---|---|---|---|
| | | | | | | | | | | | | | | cohort 1996: 82.9% vs 1997: 84.3%) (Danish 84.1% vs Descendant 78.7% vs Immigrant 75.3% vs Unknown 16.7%). | |
| [111], Bollerup S, 2017 | Quantitative, cohort | 2006 - 2014 (data collection) | Denmark | Household | N=809,656 (boys and men, aged 9-26 years in 2006-2013) | n=78,222 (Immigrant n=43,539; Descendant: n=34,683). | 9.7% (Immigrant + Descendant: 78,222/803,403). | Not specified | NATIVITY/GENERATON (Boy/Man): Danish (n=714,133; 88.2%); Descendant (n=43,539; 5.4%); Immigrant (n=34,683; 4.3%); Unknown (n=11,048; 1.4%). Note: The sum of the above figures (803,403) do not match with the denominator (809,656). BIRTH COHORT: 1979-1984 (n=147,252; 18.2%); 1984-1989 (n=152,819; 18.9%); 1989-1994 (n=173,592; 21.4%); 1994-1999 (n=172,793; 21.3%); 1999-2001 (n=66,362; 8.2%); 2001-2004 (n=96,838; 12.0%). | AGE: No average age data available. AGE GROUP (at initiation of HPV vaccination): aged <12 (3%); aged 12–15 (49%); aged 16-18 (24%); aged 19-22 (14%); aged 23-26 (10%). | Male (100%) | Recipient of vaccine (boys and men aged 9–26, living in Denmark in 2006–2014) | Self-paid HPV vaccination (for men) | National Prescription Registry: Initiation of HPV vaccine (≥1 dose): 0.8% ; Completion of HPV Vaccine (3 doses): 0.5%. Initiation of HPV vaccine: Birth cohort 1979-1984 (0.03%) vs 1984-1989 (0.4%) vs 1989-1994 (0.8%) vs 1994-1999 (1.9%) vs 1999-2001 (1.1%) vs 2001-2004 (0.1%); Boy's or man's ethnicity: Danish (both mother and son born in Denmark) 0.9% vs Descendant (mother born outside Denmark, son born in Denmark) 0.1% vs Immigrant (son born outside Denmark) 0.8% vs Unknown 0.1%. Age at initiation of vaccination: aged 12-15 (49%) vs aged 16-18 (24%) vs aged 19-22 (14%) vs aged | Low risk |

| Study ID, First author, Publication year | Study type/ design | Year(s) of study | Location/country of study | Setting | Total number of participants/ sample size (study completers) | No. of immigrants/first-generation & descendants/ second-generation | Proportion of immigrants/first-generation & descendants/second-generation | Migratory status/ Reason for migration | Country/Region of origin/ Nativity/ Ethnicity(/race) | Age | Gender/ sex | Participant type | Intervention/ programme | Uptake of HPV vaccine | Study risk of bias scores |
|---|---|---|---|---|---|---|---|---|---|---|---|---|---|---|---|
| | | | | | | | | | | | | | | 23-26 (10%) vs aged <12 (3%). | |
| [112], Bjerke RD, 2021 | Quantitative, cohort | 2009-2014 | Norway | Household | N=177,387 (girls in the first six birth cohorts eligible for HPV vaccination) | n=18,649 | 10.5% | Not specified | NATIVITY (Participating girls): Foreign-born (n=18,649; 10.5%); Norway-born (n=158,738; 89.5%).<br><br>REGION OF ORIGIN (Participating girls): Norway (n=158,738; 89.5%); Western Europe (n=2,072; 1.2%); Central and Eastern Europe (n=3,887; 2.2%); MENA (n=3,729; 2.1%); South Asia (n=3,355; 1.9%); East/ South East Asia (n=2,236; 1.3%); Sub-Saharan Africa (n=2,775; 1.6%); America and Oceania (n=595; 0.3%).<br>Note: "The percentage of girls in each of the other country background categories was 1–2%."<br><br>REGION OF ORIGIN (Parent): Norway (n=73,023; 46.0%); Europe, America, and Oceania (n=2,576; 39.3%); Middle East and Africa (n=1,121; 17.2%); Asia (n=1,091; 19.5%). | AGE: No average age data available; but "Girls in the first six birth cohorts (1997–2002) eligible for HPV vaccination" were targeted.<br><br>YEAR OF BIRTH (PROGRAMME YEAR*): 1997 (2009; n=30,209; 17.0%); 1998 (2010; n=29,719; 16.8%); 1999 (2011; n=30,100; 17.0%); 2000 (2012; n=30,098; 17.0%); 2001 (2013; n=28,932; 16.3%); 2002 (2014; n=28,329; 16.0%).<br>*"Each programme year, the vaccine was offered to only 1 birth cohort".<br>Note: Data are not disaggregated for migrants and non-migrants. | Female (100%, girls) | Recipient of vaccine | Free-of-charge, school-based HPV vaccination programme for all Norwegian girls aged 12 as part of the Norwegian Childhood Immunisation Programme (NCIP) since 2009. Girls in the first six birth cohorts eligible for HPV vaccination | Norwegian Immunisation Registry: Initiation of HPV vaccine (≥1 dose), 2009-2014: Total girls 82.5% (Girls' country background: Norway 82.6% vs non-Norwegian 81.6%; Girls' country background: East-/South East Asian 88.9% vs South Asia 87.2% vs Norway 82.6% vs MENA 82.6% vs Central- and Eastern Europe 79.7% vs Sub-Saharan Africa 76.8% vs America and Oceania 76.5% vs Western Europe 74.5%.<br>Initiation of HPV vaccine (≥1 dose): Year of birth (programme year), 2009-2014: 1997 (2009), 72.5 % vs 1998 (2010), 81.1% vs 1999 (2011), 83.5% vs 2000 (2012), 84.6% vs 2001 (2013), 86.8% vs 2002 (2014), 87.3%.<br>Initiation of HPV vaccine (≥1 dose): Region of origin (Year): Norway 72.1% (2009) vs | Low risk |

| Study ID, First author, Publication year | Study type/ design | Year(s) of study | Location/country of study | Setting | Total number of participants/ sample size (study completers) | No. of immigrants/first-generation & descendants/ second-generation | Proportion of immigrants/first-generation & descendants/second-generation | Migratory status/ Reason for migration | Country/Region of origin/ Nativity/ Ethnicity(/race) | Age | Gender/ sex | Participant type | Intervention/ programme | Uptake of HPV vaccine | Study risk of bias scores |
|---|---|---|---|---|---|---|---|---|---|---|---|---|---|---|---|
| | | | | | | | | | | | | | | 87.9% (2014); Europe, America, and Oceania 73.7% (2009) vs 77.8% (2014); Middle East and Africa 75.2% (2009) vs 81.8% (2014); Asia 80.7% (2009) vs 90.2% (2014). | |
| [113], Algren MH, 2024 | Quantitative, cohort | 2018 | Denmark | Household | N=296,461 (girls birth cohorts 1996-2005; daughter-mother dyads);<br><br>N=291,025 (daughter-father dyads) | Girls' mothers: n=38,219 (Immigrants: n=36,214; Descendants: n=2,005).<br><br>Girls' fathers: n=37,718 (Immigrants: n=36,041; Descendants: n=1,677). | Girls' mothers: 12.9% (Immigrants: 12.2%; Descendants: 0.7%).<br><br>Girls' fathers: 13.0% (Immigrants: 12.4%; Descendants: 0.6%). | Not specified | NATIVITY/GENERATION (Girls' mothers): Denmark-born (n=258,242; 87.1%); Immigrants (n=36,214; 12.2%); Descendants (n=2,005; 0.7%).<br><br>NATIVITY/GENERATION (Girls' fathers): Denmark-born (n=253,307; 87.0%); Immigrants (n=36,041; 12.4%); Descendants (n=1,677; 0.6%). | AGE: No average age data available, but girls at least aged 13 in year 2018 were targeted. | Female (100%, girls).<br><br>Girl's guardians: Both sexes. | Recipient of vaccine (but also their mothers'/ fathers' and older sister's data were also explored). Parents were defined as "girls' guardians at their 12th year of life". | Free-of-charge HPV vaccination as part of Childhood vaccination programme between 2009-2018 (for birth cohorts: 1996–2005) | n/a | Moderate risk |
| [114], Charania NA, 2023 | Quantitative, cohort | December 2021 (uptake); June 2022 (influencing parental factors) | New Zealand | Household | N=88,326 | n=23,226 (Children of migrant parents) | 26.3% | Children of migrants and refugees. PARENT VISA GROUP (N=18,132). Family (n=1,317; 7.3%); No visa | ETHNICITY (Children of migrants: N=19,410) (Children of non-migrants: N=49,929). Māori (Children of migrants: n=1,851; 9.5%) (Children of non-migrants: n=17,904; 35.9%); Pacific (Children of migrants: n=2,733; 14.1%) (Children of non-migrants: n=4,275; 8.6%); Asian (Children of migrants: n=5,880; 30.3%) (Children of non-migrants: n=1,647; 3.3%); MELAA [Middle Eastern, Latin | AGE: No average age data available.<br><br>Vaccine uptake data: Children born in NZ aged under 18 and who had spent at least 6 months in NZ as of December 2021. | Children: Female (100%) | Recipient of vaccine (female only), but also their parents' data were also explored. | Publicly-funded routine vaccination as part of National Immunisation Schedule (NIS) mainly through school-based immunis | Ministry of Health National Immunisation Register (NIR): Timeliness of vaccination (HPV 1st dose received after aged 11, and 2nd dose received before aged 14 (female children only).<br>Receipt of HPV vaccine: Children of migrant parents 83.8% vs Children | Low risk |

| Study ID, First author, Publication year | Study type/ design | Year(s) of study | Location/country of study | Setting | Total number of participants/ sample size (study completers) | No. of immigrants/first-generation & descendants/ second-generation | Proportion of immigrants/first-generation & descendants/second-generation | Migratory status/ Reason for migration | Country/Region of origin/ Nativity/ Ethnicity(/race) | Age | Gender/ sex | Participant type | Intervention/ programme | Uptake of HPV vaccine | Study risk of bias scores |
|---|---|---|---|---|---|---|---|---|---|---|---|---|---|---|---|
| | | | | | | | | required (n=6,543; 36.1%); Student (n=1,422; 7.8%); Visitor (n=4,029; 22.2%); Work (n=4,821; 26.6%). | American and African] (Children of migrants: n=480; 2.5%) (Children of non-migrants: n/a); Other (Children of non-migrants: n/a) (Children of non-migrants: n=174; 0.3%); European (Children of migrants: n=8,466; 43.6%) (Children of non-migrants: n=25,929; 51.9%).<br><br>UN REGION (N=19,482). AFRICA (n=771; 4.0%); AMERICAS (n=651; 3.3%): North America (n=471; 2.4%); South America (n/a); Central America/ Caribbean/Latin America (n/a); ASIA (n=5,241; 26.9%): Eastern Asia (n=2,463; 12.6%); Southern Asia (n=1,563; 8.0%); South-East Asia (n=1,083; 5.6%); Central and Western Asia (n/a); EUROPE (n=3,507; 18.0%): Northern Europe (n=2,841; 14.6%); Rest of Europe (n=672; 3.4%). OCEANIA (n=9,312; 47.8%): Australia and New Zealand (n=6,342; 32.6%); Micronesia and Melanesia (n=1,080; 5.5%); Polynesia (n=1,893; 9.7%). UNKNOWN (n/a). | | | | ation programme with Initial Catch-up programme at schools and primary care since 1 September 2008 (for all children aged <18, irrespective of their immigration or citizenship status) | of non-migrant parents 76.9%; Receipt of HPV vaccine on time: Children of migrant parents 82.6% vs Children of non-migrant parents 75.7%; Receipt of HPV vaccine but not on time: Children of migrant parents 1.2% vs Children of non-migrant parents 1.2%; Partial receipt of HPV vaccine or not receipt: Children of migrant parents 16.2% vs Children of non-migrant parents 23.1%. Receipt of HPV vaccine on time (full): Children of migrant parents 98.6% vs Children of non-migrant parents 98.4%. | |
| [115], Du C, 2024 | Quantitative, cohort | 2008-18 | Canada | Household | N=346,749 (2008-18); N=232,293 (2014-18). | Migrants n=31,656 (2008-18).<br><br>Migrants n=24,045 (2014-18). | Migrants 31,656 in 2008-18 (9.1% = 31,656/346,749)<br><br>Migrants 24,045 in 2014-18 (10.4% | Migrants and refugees were included, but no further details | REGION OF ORIGIN: North America (other than Canada), South America, Europe, Middle East, East Asia, Southeast Asia, South Asia, Africa and Oceania | AGE: No average age data available. "Foreign-born immigrants and refugees who arrived in Alberta from outside of Canada | Both sexes | Recipient of vaccine | Publicly-funded Alberta Health Care Insurance Plan (AHCIP | Immunization and Adverse Reaction to Immunization (Imm/ARI) database: HPV vaccine coverage (receipt of 3 doses by aged 12): HPV | Low risk |

| Study ID, First author, Publication year | Study type/ design | Year(s) of study | Location/country of study | Setting | Total number of participants/ sample size (study completers) | No. of immigrants/first-generation & descendants/ second-generation | Proportion of immigrants/first-generation & descendants/second-generation | Migratory status/ Reason for migration | Country/Region of origin/ Nativity/ Ethnicity(/race) | Age | Gender/ sex | Participant type | Intervention/ programme | Uptake of HPV vaccine | Study risk of bias scores |
|---|---|---|---|---|---|---|---|---|---|---|---|---|---|---|---|
| | | | | | | | = 24,045/232,293). | were provided. | | prior to 9 y of age" were included. | | | ) (99% of population). The HPV vaccination for those aged ≥9. Routine, school-based HPV immunization programme for females since 2008 and for males since 2014; but no school-based HPV vaccination programme in the 2018–2019 school year. | vaccine coverage (receipt of 3 doses by aged 12): During 2014-2018 (both sexes were eligible), On average – Immigrant adolescents 52.58% (95% CI: 52.03 - 53.13) vs Non-immigrants 47.41% (95% CI: 47.24 - 47.59); Sex - Female Immigrants 66.90% vs Female Non-immigrants 63.51% (p<0.0001); Male Immigrants 44.72% vs Male Non-Immigrants 39.76% (p<0.0001); Region – Southeast Asia 68.78% > South Asia 62.78% > East Asia 61.77% > Africa 60.25% > Middle East 52.77% > Europe 51.35% > South America 48.36% > Unknown/missing 46.07% > Oceania 44.83% > North American (outside Canada) 39.97%. | |
| [116], Voss SS, 2023 | Quantitative, cohort | September 2019 | Denmark | Household | N=127,661 | n=13,485 (immigrant girls, aged 14: n=3,048; descendant | 10.6% | Not specified | COUNTRY OF ORIGIN: 118 different countries in the study population, but only 13 countries (with ≤50 girls aged 14); Syria (n=758); Poland (n=271); Greenland (n=229); Germany (n=131); | AGE: No average age data available, but those aged 14 (born in 2001–2004) were targeted. | Female (100%, girls). | Recipient of vaccine (but also their parents' data were also | Free-of-charge HPV vaccination mainly provide | Danish Vaccination Register (DVR): The HPV1 vaccination at aged 14 (1st dose): Overall | Low risk |

| Study ID, First author, Publication year | Study type/ design | Year(s) of study | Location/country of study | Setting | Total number of participants/ sample size (study completers) | No. of immigrants/first-generation & descendants/ second-generation | Proportion of immigrants/first-generation & descendants/second-generation | Migratory status/ Reason for migration | Country/Region of origin/ Nativity/ Ethnicity(/race) | Age | Gender/ sex | Participant type | Intervention/ programme | Uptake of HPV vaccine | Study risk of bias scores |
|---|---|---|---|---|---|---|---|---|---|---|---|---|---|---|---|
| | | | | | | girls: n=10,437 girls). | | | Romania (n=104); Afghanistan (n=89); Iceland (n=84); Pakistan (n=65); Lithuania (n=63); Bulgaria (n=61); Iran (n=58); Somalia (n=56); and Iraq (n=54). | | | explored as part of assessment of determinants) | d by GPs as part of Danish childhood vaccination programme in 2009 (no school vaccinations during the study period) | 72%; Country of origin - Syria 28% vs Greenland 22% vs Poland 45%.; Girl's ancestry – Dane 73% vs Descendant 73% vs Immigrant 43%.<br><br>Full HPV vaccinated (2nd or 3rd depending on age and interval between vaccinations): Girl's ancestry – Dane 53% vs Descendant 49% vs Immigrant 27%. | |
| [117], Scarinci IC, 2020 | Quantitative, RCT | May 2013 - October 2017 (enrolment) | USA | Community | N=278 (Latinx immigrant mothers with daughters aged 9–12 [a total of 278 mother-daughter dyads]) | n=278 | 100.0% | Not specified | Not mentioned but only Latinx. | AGE (Mother): i) HPV vaccination arm – Mean 35.4 years (SD=5.9); ii) Control arm - Mean 34.8 years (SD=5.1) [p=0.302]. All: Mean 35 years.<br>AGE (Daughter): i) HPV vaccination arm – Mean 9.8 years (SD=0.9); ii) Control arm - Mean 9.8 years (SD=1.0) [p=0.813]. | Female (100%) | Parent (Mother); Recipient of vaccine (Daughter). | Community- and theory-based, culturally appropriate intervention to enhance HPV vaccination uptake for daughters of Latina migrants | Self-report: Completion of dose in daughters 7 months after the intervention (Community- and theory-based, culturally appropriate intervention): 1 dose 52.2%; 2nd dose 40.4%; 3rd dose 32.3%. | Moderate risk |

**Table S6. A summary of qualitative and quantitative findings**

| Theme | Sub-theme | Code | Qualitative studies/ components | | | | Quantitative studies/ components | | | |
|---|---|---|---|---|---|---|---|---|---|---|
| | | | Study ID number | | Total No. of participants (No. of immigrants/first-generation & descendants/second-generation) | | Study ID number | | Total No. of participants (No. of immigrants/first-generation & descendants/second-generation) | |
| | | | Negatively/positively influencing | Not predominant | Negatively/ positively influencing | Not predominant | Negatively/positively influencing | Not significant | Negatively/positively influencing | Not significant |
| **Thoughts and feelings** | **1-A) Trust/confidence** | 1-a) Confidence in vaccine benefits? | [1]; [2]; [4]; [6]; [8]; [10]; [15]; [16]; [17]; [19]; [23]; [24]; [25]; [26]; [27]; [28]; [32]; [35]; [36]. | [8]. | 772 (540) | 40 (22) | [1]; [5]; [72]; [80]; [92]. | [4]; [57]; [92]; [97]; [99]; [100]. | 958 (958) | 1,852 (1,159) |
| | | 1-b) Confidence in vaccine safety? | [1]; [2]; [4]; [9]; [10]; [11]; [12]; [14]; [15]; [16]; [17]; [19]; [21]; [24]; [25]; [27]; [28]; [31]; [32]; [35]; [36]; [37]. | [21]. | 647 (524) | 36 (36) | [1]; [5]; [80]; [92]; [94]; [95]; [97]; [99]; [100]. | [4]; [5]; [7]; [57]; [69]. | 1,851 (1,363) | 780 (575) |
| | | 1-c) Trust in vaccine providers? | [1]; [2]; [8]; [9]; [10]; [19]; [20]; [24]; [30]; [31]; [32]; [36]; [37]; [38]. | n/a | 364 (238) | n/a (n/a) | [4]. | n/a | 70 (51) | n/a (n/a) |
| | **1-B) Perceived parental responsibility/self-efficacy in action** | 1-d) Perceived parental responsibility? | [4]; [6]; [11]; [12]; [13]; [14]; [23]; [25]; [28]; [32]; [35]; [37]. | [28]; [35]. | 400 (282) | 55 (55) | [10]; [94]. | n/a | 172 (172) | n/a (n/a) |
| | | 1-e) Confidence in engaging in preventive action? | [8]; [21]; [23]. | n/a | 87 (69) | n/a (n/a) | [69]; [73]; [88]; [92]; [95]. | [92]; [95]; [97]. | 1,254 (1,254) | 771 (771) |
| | **1-C) Perceived risks; perceptions of vaccination unnecessary** | 1-f) Perceived risk (self)? | [11]; [18]; [23]; [31]; [34] | n/a | 112 (105) | n/a (n/a) | [73]; [88]. | [70]; [73]; [88]; [117]. | 630 (630) | 2,242 (2,242) |
| | | 1-g) Perceived risk (child)? | [4]; [11]; [12]; [19]; [20]; [28]; [32]. | n/a | 165 (106) | n/a (n/a) | [73]. | [88]; [117]. | 317 (317) | 595 (595) |
| | | 1-h) Vaccination unnecessary? | [2]; [4]; [7]; [9]; [11]; [13]; [15]; [18]; [20]; [22]; [23]; [24]; [27]; [30]; [31]; [34]; [37]. | n/a | 662 (535) | n/a (n/a) | [1]; [2]; [5]; [80]; [92]; [94]; [97]. | [4]; [7]; [80]; [88]; [92]; [99].; [100]. | 998 (987) | 1,760 (1,253) |
| | **1-D) Perceived severity** | 1-i) Perceived severity? | [15]; [17]; [18]; [19]; [23]. | n/a | 150 (115) | n/a (n/a) | [72]; [92]; [97]. | [92]; [99]; [100]. | 1,065 (1,065) | 1,137 (649) |

| | | | | | | | | | | |
|---|---|---|---|---|---|---|---|---|---|---|
| | **1-E) Perceived uncertainty** | 1-J) Perceived uncertainty (perceived information needs)? | [1]; [2]; [4]; [6]; [8]; [9]; [11]; [13]; [15]; [16]; [17]; [18]; [19]; [20]; [23]; [25]; [27]; [28]; [32]; [36]; [37]. | N/A | 738 (547) | n/a (n/a) | [7]; [69]; [70]; [72]; [80]; [97]; [99]. | [80]. | 2,547 (2,547) | 74 (74) |
| | **1-F) Worries/fears** | 1-k) Worry about future HPV infection? | [28]. | N/A | 25 (25) | n/a (n/a) | [73]; [88]. | [70]; [88]; [117]. | 630 (630) | 1,929 (1,929) |
| | | 1-L) Fear of pain/needles? | [10]; [16]; [25]; [27]; [37]. | [8]. | 122 (107) | 40 (22) | [99]. | [7]. | 173 (173) | 162 (162) |
| | **1-G) Cultural beliefs vs. Reality (children's sexual activity)** | 1-m) Cultural/religious beliefs/values? | [2]; [4]; [7]; [10]; [11]; [13]; [15]; [16]; [18]; [19]; [20]; [23]; [24]; [25]; [26]; [27]; [30]; [32]; [34]; [35]; [37]. | [9]. | 886 (667) | 31 (31) | [1]; [69]; [80]; [94]; [97]. | [5]; [7]. | 646 (646) | 212 (212) |
| | | 1-n) More realistic understanding of children's sexual activity? | [6]; [21]; [28]; [30]. | n/a | 168 (94) | n/a (n/a) | n/a | n/a | n/a (n/a) | n/a (n/a) |
| | **1-H) Perceived priority or normality** | 1-o) Perceived priority or normality of HPV vaccine (user/*provider)? | [7]; [9]; [10]; [13]; [14]; [19]; [20]; [21]; [25]; [28]; [30]; [32]; [36]; [37]. | n/a | 548 (438) | n/a (n/a) | [49]. | [87]. | 47 (47) | 269 (269) |
| | **1-I) Honoured feeling** | 1-p) Feel honoured to receive HPV vaccination? | [6]; [10]; [32]. | n/a | 94 (41) | n/a (n/a) | n/a | n/a | n/a (n/a) | n/a (n/a) |
| | **1-J) Awareness** | 1-q) Awareness of HPV/HPV vaccine | [7]; [9]; [10]; [11]; [17]; [18]; [19]; [20]; [21]; [23]; [24]; [25]; [26]; [33]; [36]; [37]. | [14]; [24]. | 703 (549) | 55 (48) | [4]; [5]; [7]; [39]; [43]; [49]; [51]; [55]; [57]; [58]; [60]; [66]; 64]; [70]; [72]; [73]; [74]; [76]; [78]; [81]; [87]; [89]; [96]; [100]. | [5]; [45]; [49];[50]; [51]; [55]; [58]; [66]; [70]; [72]; [76]; [77]; [78]; [81]; [87]; [88]; [96]. | 25,562 (9,978) | 17,285 (6,205) |
| | **1-K) Knowledge** | 1-r) Knowledge/health literacy of HPV/HPV vaccine | [1]; [2]; [7]; [8]; [9]; [10]; [11]; [13]; [14]; [15]; [17]; [18]; [19]; [20]; [21]; [22]; [23]; [24]; [25]; [26]; [27]; [28]; [32]; [34]; [35]; [36]; [37]; [38]. | [14]; [27]. | 1,100 (871) | 77 (77) | [1]; [2]; [3]; [4]; [5]; [7]; [9]; [39]; [41]; [54]; [55]; [61]; [66]; [69]; [72]; [74]; [80]; [81]; [85]; [87]; [88]; [92]; [95]; [96]; [97]; [99]. | [39]; [41]; [51]; [54]; [55]; [57]; [61]; [77]; [78]; [80]; [81]; [85]; [87]; [89]; [92]; [94]; [97]; [100]. | 11,351 (7,567) | 6,228 (3,969) |
| **Social processes** | **2-A) Champion/encouragement** | 2-a) Influential others support vaccination? | [29]; [31]; [32]. | n/a | 47 (13) | n/a (n/a) | n/a | n/a | n/a (n/a) | n/a (n/a) |
| | | 2-b) Heard experience of those vaccinated? | [7]; [12]; [15]; [24]; [30]; [35]. | n/a | 282 (230) | n/a (n/a) | n/a | n/a | n/a (n/a) | n/a (n/a) |
| | **2-B) Social norms/institutional recommendations** | 2-c) Social norms? | [14]. | n/a | 36 (36) | n/a (n/a) | [69]; [95]; [97]; [99]. | n/a | 683 (683) | n/a (n/a) |
| | | *2-d) Institutional recommendations? | [14]; [16]; [26]; [33]; [36]; [38]. | n/a | 336 (247) | n/a (n/a) | n/a | n/a | n/a (n/a) | n/a (n/a) |
| | **2-C) Decision autonomy** | 2-e) Decision autonomy? | [1]; [4]; [5]; [10]; [11]; [12]; [14]; [17]; [24]; [32]; [34]; [35]; [37]. | n/a | 403 (304) | n/a (n/a) | [73]. | [88]. | 313 (313) | 317 (317) |

| | | | | | | | | | | |
|---|---|---|---|---|---|---|---|---|---|---|
| | **2-D) Message framing** | *2-f) Message framing? | [9]; [15]; [16]; [20]; [28]; [32]; [36]. | n/a | 187 (154) | n/a (n/a) | n/a | n/a | n/a (n/a) | n/a (n/a) |
| | **2-E) Information via healthcare providers/schools/commercial (educational materials, brochures)** | *2-g) Information on HPV vaccine available (educational materials, brochures)? | [9]; [14]; [17]; [19]; [21]; [32]; [35]; [36]; [38]. | n/a | 295 (235) | n/a (n/a) | [1]; [39]; [87]. | [41]. | 816 (344) | 200 (200) |
| | | *2-h) Information via healthcare providers (/schools)? | [1]; [3]; [7]; [9]; [10]; [11]; [14]; [16]; [21]; [25]; [26]; [31]; [35]; [36]; [38]. | [27]. | 876 (713) | 41 (41) | [1]; [3]; [4]; [39]; [41]; [49]; [51]; [70]; [72]; [80]; [85]; [99]. | [3]; [5]; [51]; [70]; [94]. | 4,001 (2,896) | 2,406 (1,792) |
| | | 2-i) Information via commercial? | [18]; [25]. | n/a | 57 (57) | n/a (n/a) | [1]; [3]; [41]; [80]. | [3]; [49]. | 533 (459) | 275 (201) |
| | **2-F) Information/ support via social network** | 2-j) Information/support via social network? | [7]; [10]; [11]; [12]; [13]; [14]; [16]; [17]; [18]; [21]; [24]; [27]; [29]; [31]; [32]; [35]; [36]; [37]; [38]. | n/a | 711 (585) | n/a (n/a) | [3]; [4]; [51]; [72]; [85]; [99]. | [3]; [5]; [41]; [49]; [51]. | 1,799 (1,166) | 1,155 (541) |
| | **2-G) Information via traditional media (in local/native language)** | 2-k) Information via traditional platforms (local language)? | [10]; [14]; [19]; [25]; [27]; [36]. | n/a | 176 (156) | n/a (n/a) | [3]; [4]; [41]; [49]; [72]; [85]; [92]. | [3]; [49]; [92]; [97]. | 1,619 (1,526) | 913 (839) |
| | | 2-L) Information via traditional platform (native language)? | [12]; [13]; [14]; [17]; [24]; [27]. | n/a | 240 (175) | n/a (n/a) | [70]. | [70]. | 1,334 (1,334) | 1,334 (1,334) |
| | **2-H) Information via internet/social media** | 2-m) Information via internet? | [10]; [11]; [14]; [15]; [19]; [28]; [31]; [36]. | n/a | 190 (170) | n/a (n/a) | [3]; [70]; [80]; [96]. | [3]; [49]. | 1,914 (1,840) | 275 (201) |
| | | 2-n) Information via social media? | [10]; [18]; [24]; [31]; [36]; [37]. | n/a | 172 (138) | n/a (n/a) | n/a | n/a | n/a (n/a) | n/a (n/a) |
| | **2-I) Self-efficacy in finding information** | 2-o) Finding their own information? | [28]; [36]; [37]. | n/a | 113 (86) | n/a (n/a) | [5]; [57]; [70]. | n/a | 1,767 (1,581) | n/a (n/a) |
| | **2-J) Doctor's recommendation** | *2-p) Doctor's recommendation? | [1]; [3]; [4]; [5]; [7]; [9]; [10]; [13]; [15]; [17]; [19]; [20]; [21]; [23]; [24]; [27]; [28]; [30]; [32]; [35]; [36]; [38]. | n/a | 1,040 (818) | n/a (n/a) | [5]; [7]; [60]; [66]; [87]; [88]; [99]. | [4]; [7]; [50]; [67]; [88]; [97]. | 5,554 (1,363) | 5,533 (2,261) |
| | **2-K) (Grand) Mother-daughter interaction** | 2-q) Mother (grandmother)-daughter interaction (including spouse)? | [2]; [4]; [5]; [10]; [11]; [13]; [14]; [15]; [16]; [17]; [19]; [23]; [28]; [34]; [35]; [37]; [38]. | [28]; [32]. | 558 (451) | 38 (25) | [1]; [97]; [99]. | n/a | 466 (466) | n/a (n/a) |
| | **2-L) Self-efficacy in patient-provider interaction** | *2-r) Self-confidence in answering question (patient-provider communication)? | [20]. | n/a | 10 (2) | n/a (n/a) | [2]. | n/a | 41 (30) | n/a (n/a) |
| | **2-M) Social stigma** | 2-s) Social stigma? | [1]; [2]; [8]; [9]; [14]; [18]; [20]; [32]; [34]. | n/a | 280 (224) | n/a (n/a) | [94]; [100]. | [97]. | 752 (264) | 262 (262) |

| | | | | | | | | | | |
|---|---|---|---|---|---|---|---|---|---|---|
| | **2-N) Cultural/racial/gender concordance, gender equality** | *2-t) Cultural/racial/gender concordance? | [1]; [20]; [30]; [32]; [35]; [36]. | n/a | 176 (109) | n/a (n/a) | n/a | [64]. | n/a (n/a) | 187 (61) |
| | | 2-u) Gender equality? | [7]; [9]; [10]; [11]; [20]; [24]. | n/a | 240 (225) | n/a (n/a) | [94]. | n/a | 164 (164) | n/a (n/a) |
| | **2-O) Communication methods** | 2-v) Communication methods? | [15]; [20]; [27]; [35]; [36]; [37]. | n/a | 189 (154) | n/a (n/a) | n/a | n/a | n/a (n/a) | n/a (n/a) |
| | **2-P) Non-factual/negative/misinformation** | *2-w) Non-factual information? | [32]; [36] | n/a | 71 (46) | n/a (n/a) | n/a | n/a | n/a (n/a) | n/a (n/a) |
| | | 2-x) Seeing negative information? | [7]; [13]; [14]; [31]; [32]. | n/a | 296 (263) | n/a (n/a) | n/a | n/a | n/a (n/a) | n/a (n/a) |
| | | 2-y) Spread of misinformation? | [2]; [7]; [10]; [11]; [23]; [32]; [36]; [37]. | n/a | 333 (282) | n/a (n/a) | n/a | n/a | n/a (n/a) | n/a (n/a) |
| **Motivation** | **3-A) Attitude toward HPV vaccination** | 3-a) Willingness to vaccinate in the near future? | [1]; [5]; [8]; [10]; [12]; [17]; [21]; [23]; [24]; [28]; [29]; [30]; [31]; [33]; [34]; [36]; | [21]. | 474 (337) | 36 (36) | [1]; [5]; [7]; [39]; [60]; [69]; [72]; [88]; [89]; [99]. | [5]; [7]; [50]; [51]; [89]. | 2,635 (2,163) | 1,682 (1,140) |
| | | 3-b) Intend to vaccinate? | [27]; [37]. | n/a | 71 (56) | n/a (n/a) | [59]; [69]; [73]; [88]; [94]; [95]. | [4]; [59]; [88]; [94]. | 1,270 (1,196) | 779 (686) |
| | | 3-c) Hesitant to vaccinate? | [10]; [34]; [36]. | n/a | 100 (81) | n/a (n/a) | [1]; [5]; [7]; [73]; [88]; [95]; [101]. | [95]; [101]. | 162,534 (18,027) | 161,661 (17,154) |
| | **3-B) Willingness to communicate/consult** | 3-d) Willingness to communicate with child/spouse/parents for decision-making? | [15]; [17]; [24]; [37]. | n/a | 124 (75) | n/a (n/a) | n/a | n/a | n/a (n/a) | n/a (n/a) |
| | | 3-e) Willingness to consult with health providers? | [1]; [17]; [32]; [37]. | n/a | 129 (74) | n/a (n/a) | [69]; [87]. | n/a | 384 (384) | n/a (n/a) |
| | **3-C) Willingness to recommend** | *3-f) Willingness to recommend HPV vaccines? | [7]; [32]. | n/a | 175 (162) | n/a (n/a) | [2]. | n/a | 41 (30) | n/a (n/a) |
| | **3-D) Willingness to learn about HPV/HPV vaccines** | 3-g) Willingness to learn about HPV/HPV vaccines? | [1]; [14]; [17]; [36]; [37]. | n/a | 210 (156) | n/a (n/a) | n/a | n/a | n/a (n/a) | n/a (n/a) |
| **Practical issues** | **4-A) Prior practical knowledge/experience** | 4-a) Know where vaccines (/info) are available? | [10]; [20]; [32]; [36]. | n/a | 89 (56) | n/a (n/a) | [57]; [69]; [72]; [80]; [100]. | n/a | 1,587 (912) | n/a (n/a) |
| | | 4-b) Previous uptake of vaccination (incl. home country)? | [14]; [24]; [25]; [36]; [37]. | n/a | 156 (122) | n/a (n/a) | [101]; [109]. | [101]; [109]. | 171,220 (18,407) | 171,220 (18,407) |
| | **4-B) Existing (/strength of) connection to healthcare** | 4-c) Having a usual place to seek care? Number of healthcare office visits in the past year? | [19]; [20]; [21]; [23]; [32]; [34]; [37]. | n/a | 154 (103) | n/a (n/a) | [52]; [53]. | [52]; [53]; [61]; [88]; [96]. | 21,644 (3,510) | 22,482 (4,348) |

| | | | | | | | | | |
|---|---|---|---|---|---|---|---|---|---|
| | **4-C) Accessibility** | 4-d) Ease of access (user/*provider)? | [5]; [9]; [19]; [20]; [25]; [28]; [32]; [36]; [37]. | n/a | 250 (194) | n/a (n/a) | [70]; [80]. | [87]. | 1,408 (1,408) | 269 (269) |
| | **4-D) Appropriateness** | *4-e) Native language speaking personnel/translation available? Capability of interpreters? | [7]; [9]; [19]; [25]; [30]; [31]; [32]; [34]; [36]; [37]; [38]. | n/a | 424 (335) | n/a (n/a) | [39]. | n/a | 516 (44) | n/a (n/a) |
| | **4-E) Acceptability** | 4-f) Preferred site/branded vaccines? Quality of care? | [19]; [31]; [36]. | n/a | 91 (71) | n/a (n/a) | [97]. | n/a | 262 (262) | n/a (n/a) |
| | **4-F) Availability** | *4-g) Availability of on-site vaccination (incl. home country)? | [7]; [19]; [25]; [32]; [33]; [38]. | n/a | 246 (225) | n/a (n/a) | n/a | n/a | n/a (n/a) | n/a (n/a) |
| | **4-G) Affordability** | 4-h) Cost (user/*provider)? | [8]; [10]; [17]; [19]; [20]; [21]; [23]; [27]; [29]; [31]; [32]; [33]; [36]; [37]. | [21]. | 378 (256) | 36 (36) | [57]; [60]; [80]; [97]; [100]. | [5]; [51]; [87]. | 1,367 (693) | 949 (409) |
| | **4-H) Coordination/compatibility/continuity of care** | *4-i) Coordination/compatibility (e.g. insurance, vaccine schedules) or continuity of care? | [25]; [31]; [32]; [36]. | n/a | 97 (72) | n/a (n/a) | [96]. | n/a | 278 (278) | n/a (n/a) |
| | **4-I) Vaccine reminder/registry systems** | *4-J) Vaccine reminder/tracking systems in place? | [25]; [32]. | n/a | 26 (13) | n/a (n/a) | [109]. | [109]. | 9,692 (1,386) | 9,692 (1,386) |
| | | *4-k) Immunisation registry system in place? | [15]; [29]; [32]; [36]; [38]. | n/a | 128 (82) | n/a (n/a) | n/a | n/a | n/a (n/a) | n/a (n/a) |
| **Socio-demographic and other factors** | **5-A) Nativity** | 5-a) Nativity | [6]; [17]; [26]; [30]; [32]; [36]. | [6]; [17]; [26]; [37]. | 407 (204) | 332 (173) | [3]; [6]; [8]; [39]; [40]; [42]; [46]; [48]; [51]; [52]; [53]; [54]; [55]; [56]; [57]; [59]; [62]; [63]; [65]; [66]; [67]; [68]; [71]; [74]; [75]; [78]; [79]; [83]; [90]; [91]; [93]; [101]; [103]; [104]; [105]; [106]; [107]; [108]; [110]; [111]; [113]; [115]; [116]. | [3]; [6]; [39]; [44]; [48]; [51]; [52]; [53]; [54]; [56]; [57]; [58]; [59]; [62]; [63]; [64]; [65]; [66]; [67]; [68]; [77]; [78]; [82]; [83]; [84]; [86]; [90]; [101]; [103]; [104]; [106]; [108]; [112]; [114]. | 4,868,848 (841,210) | 2,321,940 (556,391) |
| | **5-B) Generational status** | 5-b) Generational status | [14]; [16]; [22]. | [26]. | 86 (80) | 174 (97) | [76]; [101]; [105]; [107]; [110]; [113]; [116]. | [3]; [45]; [76]; [101]; [107]; [111]. | 1,249,380 (184,048) | 1,237,568 (123,647) |
| | **5-C) Country (/region) of origin** | 5-c) (Parental) Country (/region) of origin | [4]; [6]; [13]; [17]; [20]; [24]; [26]; [35]. | [4]; [17]; [26]. | 503 (292) | 299 (176) | [4]; [40]; [43]; [47]; [48]; [51]; [53]; [56]; [62]; [63]; [68]; [72]; [81]; [91]; [93]; [102]; [104]; [105]; [106]; [107]; [109]; [112]; [114]; [115]; [116]. | [4]; [5]; [44];[48]; [51]; [56]; [63]; [77]; [81]; [93]; [102]; [105]; [109]; [112]; [114]. | 2,740,378 (422,546) | 654,140 (135,904) |
| | **5-D) Race/ethnicity** | 5-d) (Parental) Race/ethnicity | [6]; [13]; [17]; [30]; [32]. | [17]. | 247 (113) | 55 (28) | [6]; [52]; [58]; [62]; [66]; [67]; [68]; [71]; [89]; [92]; [114]. | [6]; [40]; [52]; [53]; [57]; [66]; [67]; [84]; [89]; [94]; [98]; [114]. | 157,368 (39,090) | 157,831 (36,994) |
| | **5-E) Migratory** | 5-e) Migrant status | [15]; [19]; [32]; [36]. | [32]. | 111 (78) | 13 (n/a) | [92]; [114]. | [5]; [106]; [114]. | 88,702 (23,602) | 111,224 (26,540) |

| | | | | | | | | | | |
|---|---|---|---|---|---|---|---|---|---|---|
| | **issues/conditions** | 5-f) Duration of residence | [13]; [29]. | [11]. | 93 (52) | 10 (10) | [3]; [7]; [8]; [43]; [48]; [56]; [58]; [59]; [62]; [70]; [79]; [81]; [92]; [105]; [106]; [114]. | [3]; [40]; [41]; [48]; [53]; [55]; [59]; [72]; [77]; [81]; [86]; [88]; [89]; [90]; [106]. | 448,351 (116,423) | 96,618 (15,968) |
| | | 5-g) Migrant mobility | [19]; [25]; [26]; [28]; [29]; [31]; [32]; [37]; [38]. | n/a | 325 (191) | n/a (n/a) | [107]. | n/a | 260,251 (26,539) | n/a (n/a) |
| | | 5-h) Language skills (user/parents) | [3]; [7]; [13]; [19]; [20]; [22]; [28]; [31]; [32]; [34]; [36]. | n/a | 655 (507) | n/a (n/a) | [3]; [7]; [39]; [55]; [56]; [61]; [114]; [116]. | [3]; [5]; [7]; [45]; [56]; [94]; [96]. | 217,477 (37,539) | 1,945 (1,470) |
| | | 5-i) Acculturation | [14]; [16]; [18]; [28]. | n/a | 135 (135) | n/a (n/a) | [3]; [43]; [81]; [92]. | [3]; [4]; [42]; [44]; [49]; [50]; [70]; [78]; [81]; [92]; [97]; [99]. | 928 (847) | 5,445 (3,267) |
| | | 5-J) Citizenship | [32]. | [32]. | 13 (n/a) | 13 (n/a) | [40]; [62]. | [53]. | 49,525 (9,416) | 14,056 (2,396) |
| | | 5-k) Health insurance status | [10]; [19]; [21]; [25]; [32]; [37]. | [32]. | 120 (84) | 13 (n/a) | [52]; [88]. | [52]; [53]; [61]; [88]; [94]. | 7,905 (1,431) | 22,368 (4,234) |
| | **5-F) Education, income, occupation** | 5-L) Educational attainment | [14]; [30]; [32]. | n/a | 83 (36) | n/a (n/a) | [5]; [7]; [52]; [61]; [63]; [70]; [72]; [73]; [75]; [112]; [114]. | [5]; [7]; [41]; [49]; [53]; [70]; [85]; [87]; [88]; [89]; [92]; [94]; [99]; [114]. | 592,732 (46,061) | 106,529 (29,769) |
| | | *5-m) Course specialty, Provider specialty | n/a | n/a | n/a (n/a) | n/a (n/a) | n/a | [87]; [92]. | n/a (n/a) | 645 (645) |
| | | 5-n) Income | [21]; [30]; [32]. | n/a | 83 (36) | n/a (n/a) | [7]; [49]; [52]; [75]; [112]; [114]. | [7]; [49]; [52]; [61]; [65]; [85]; [87]; [88]; [89]; [96]; [99]; [112]; [115]. | 585,166 (43,198) | 568,825 (53,963) |
| | | 5-o) Occupation | n/a | n/a | n/a (n/a) | n/a (n/a) | [72]. | [41]; [85]; [88]; [94]. | 427 (427) | 952 (952) |
| | **5-G) Age** | 5-p) Age | [18]; [20]; [27]. | n/a | 95 (87) | n/a (n/a) | [39]; [61]; [62]; [63]; [70]; [72]; [89]; [92]; [93]; [96]; [98]; [103]; [111]. | [5]; [41]; [52]; [53]; [70]; [85]; [87]; [88]; [92]; [97]; [103]. | 837,121 (88,005) | 25,140 (6,642) |
| | **5-H) Gender** | 5-q) Gender/sex | [11]; [13]; [18]; [19]; [24]; [25]; [26]; [27]; [34]. | [21]; [34]. | 427 (308) | 70 (63) | [41]; [43]; [49]; [52]; [59]; [63]; [65]; [70]; [72]; [73]; [79]; [81]; [83]; [91]; [92]; [93]; [96]; [102]. | [5]; [41]; [49]; [52]; [59]; [69]; [81]; [83]; [89]; [94]; [97]; [98]; [99]; [102]; [104]; [113]; [115]. | 358,926 (59,666) | 1,436,282 (223,891) |
| | **5-I) Sexually active; number of children** | 5-r) Marriage status, sexually active | [6]; [18]; [23]; [27]; [34]. | n/a | 203 (156) | n/a (n/a) | [5]; [63]; [72]; [73]; [85]; [89]; [96]; [114]. | [52]; [53]; [61]; [70]; [88]. | 95,695 (25,892) | 23,538 (5,404) |
| | | 5-s) Risky sexual behaviours? | [27]. | n/a | 41 (41) | n/a (n/a) | [72]. | [99]. | 427 (427) | 173 (173) |
| | | 5-t) Number of children? | n/a | n/a | n/a (n/a) | n/a (n/a) | [72]. | [85]; [88]. | 427 (427) | 588 (588) |
| | **5-J) Recent pap test or (family) history of vaccine preventable diseases** | 5-u) Received a pap test (e.g. in the past 3 years/ 12 months)? | [23]. | n/a | 11 (11) | n/a (n/a) | [39]; [70]. | [39]. | 1,850 (1,378) | 516 (44) |
| | | 5-v) Previous experience/family history with vaccine preventable diseases (e.g. cervical | [6]; [10]; [12]; [24]; [29]; [30]; [32]. | n/a | 185 (59) | n/a (n/a) | [4]; | [61]; [87]; [94]; [96]; [99]. | 70 (51) | 1,127 (1,127) |

| | | | | | | | | | | |
|---|---|---|---|---|---|---|---|---|---|---|
| | | cancer) or abnormal pap test results? | | | | | | | | |
| | **5-K) Health status** | 5-w) Health status | [23]; [37]. | n/a | 41 (26) | n/a (n/a) | [61]. | [53]; [96]. | 243 (243) | 14,334 (2,674) |
| | **5-L) Birth cohort** | 5-x) Birth cohort | [18]. | n/a | 44 (44) | n/a (n/a) | [101]; [107]; [115]. | [110]. | 768,528 (75,216) | 127,088 (11,779) |
| | **5-M) Place of residence** | 5-y) Place of residence | [31]. | n/a | 13 (13) | n/a (n/a) | [41]; [52]; [53]; [114]; [115]. | [52]; [53]; [114]. | 456,919 (58,592) | 109,970 (26,736) |
| | **5-N) Religion** | 5-z) Religion | [34]; [36]. | n/a | 92 (73) | n/a (n/a) | [7]. | [7]; [99]. | 162 (162) | 335 (335) |
| **HPV vaccination programme design and delivery methods** | **6-A) Timing of HPV vaccination** | *6-a) HPV vaccine catch-up | [9]; [20]; [23]. | n/a | 52 (44) | n/a (n/a) | [42]; [47]; [48]; [103]; [104]; [105]; [106]; [108]; [114]. | [42]; [104]; [106]; [114]. | 2,506,034 (603,309) | 801,050 (163,871) |
| | | *6-b) Opportunistic | n/a | n/a | n/a (n/a) | n/a (n/a) | [39]; [104]; [108]. | [108]. | 1,730,769 (475,293) | 1,040,577 (338,002) |
| | | *6-c) Routine/ordinary | n/a | n/a | n/a (n/a) | n/a (n/a) | [6]; [42]; [102]; [106]; [107]; [114]; [115]. | [42]; [106]; [114]; [115]. | 809,289 (97,413) | 458,123 (58,300) |
| | **6-B) Payment mode of HPV vaccination** | *6-d) Subsidised | [32]; [36]. | n/a | 71 (46) | n/a (n/a) | [39]; [104]. | n/a | 690,192 (137,271) | n/a (n/a) |
| | | *6-e) Free of charge/safety net | [4]; [9]; [20]; [32]. | n/a | 124 (84) | n/a (n/a) | [4]; [46]; [48]; [70]; [102]; [103]; [104]; [105]; [107]; [108]; [112]. | [51]; [82]; [104]; [107]; [113]. | 2,540,498 (611,207) | 1,244,768 (202,235) |
| | | *6-f) Self-paid | [21]; [33]; [36]. | [21]. | 116 (104) | 36 (36) | [39]; [108]; [111]. | [105]. | 1,850,749 (416,288) | 274,154 (76,135) |
| | **6-C) Venue type for HPV vaccination** | *6-g) School-based | [6]; [9]; [20]; [26]; [38]. | n/a | 304 (179) | n/a (n/a) | [6]; [75]; [102]; [103]; [104]; [112]; [114]; [115]; [116]. | [104]; [114]; [115]. | 1,832,787 (236,870) | 1,124,751 (192,109) |
| | **6-D) Legality of HPV vaccination** | *6-h) Mandatory | [6]; [12]; [34]. | n/a | 124 (53) | n/a (n/a) | [6]. | n/a | 73 (33) | n/a (n/a) |
| | | *6-i) Optional | [20]; [32]; [36]; | n/a | 81 (48) | n/a (n/a) | n/a | n/a | n/a (n/a) | n/a (n/a) |
| **HPV vaccination outcomes** | **7-A) Demand/seek to initiate HPV vaccination** | 7-a) Demand on HPV vaccination | [29]; [30]; [31], | n/a | 68 (13) | n/a (n/a) | [6]; [60]. | n/a | 133 (93) | n/a (n/a) |
| | | 7-b) Seeking behaviour for HPV vaccination | [12]; [21] | n/a | 53 (42) | n/a (n/a) | n/a | n/a | n/a (n/a) | n/a (n/a) |
| | **7-B) Acceptance/ delay/refusal** | 7-c) Acceptance/delay/refusal of HPV vaccines | [5]; [7]; [10]; [11]; [12]; [13]; [14]; [15]; [16]; [17]; [18]; [20]; [21]; [22]; [24]; [26]; [27]; [30]; [31]; [32]; [34]; [35]; [36]; [37]; [38]. | [11]; [14]; [21]. | 1,032 (795) | 82 (82) | [1]; [6]; [7]; [10]; [39]; [60]; [67]; [87]; [97]; [99]. | [4]; [45]; [57]; [67]; [97]; [99]. | 6,220 (2,4457) | 6,504 (2,689) |
| | **7-C) HPV vaccine uptake/initiation/completion** | 7-d) HPV vaccination behaviour (initiation) | [12]; [25]; [34]. | n/a | 64 (46) | n/a (n/a) | [41]; [42]; [43]; [46]; [47]; [52]; [53]; [54]; [60]; [62]; [63]; [65]; [68]; [71]; [72]; [75]; [79]; [87]; [90]; [92]; [93]; [95]; [97]; [100]; [101]; [102]; [103]; [104]; | [41]; [42]; [44]; [52]; [53]; [63]; [65]; [82]; [84]; [86]; [90]; [92]; [93]; [95]; [97]; [101]; [103]; [105]; [106]; [107]; [109]; [111]; [112]; [113]; [115]. | 5,303,628 (856,511) | 2,441,520 (299,672) |

| | | | | | | | | | |
|---|---|---|---|---|---|---|---|---|---|
| | | | | | | | [105]; [106]; [107]; [108]; [109]; [110]; [111]; [112]; [113]; [115]; [116]; [117]. | | | |
| | | 7-e) HPV vaccination behaviour (completion of series) | [12]; [19]; [25]; [34]; [37]. | n/a | 114 (73) | n/a (n/a) | [43]; [48];[52]; [53]; [63]; [79]; [87]; [100]; [103]; [104]; [108]; [110]; [113]; [114]; [115]; [116]; [117]. | [42]; [48]; [52]; [53]; [62]; [90]; [97]; [102]; [103]; [104]; [108]; [113]; [114]; [115]; [117]. | 2,787,108 (606,154) | 2,609,685 (588,582) |
| | | 7-f) HPV vaccination behaviour (partial completion) | n/a | n/a | n/a (n/a) | n/a (n/a) | [48]; [97]; [117]. | [48]; [97]; [117]. | 5,507 (1,080) | 5,507 (1,080) |
| | | 7-g) HPV vaccine uptake (if no details mentioned) | [2]; [4];[7]; [8]; [9]; [10]; [13]; [15]; [17]; [18]; [19]; [20]; [23]; [26]; [28]; [31]; [32]; [33]; [35]; [36]; [37]; [38]. | [8]; [9]; [28]. | 965 (737) | 96 (78) | [6]; [7]; [8]; [40]; [56]; [59]; [72]; [76]; [80]; [81]; [83]; [87]; [91]; [97]; [98]; [103]. | [4]; [6]; [40]; [50]; [56]; [59]; [64]; [80]; [81]; [83]; [87]; [98]; [103]. | 217,462 (42,354) | 41,673 (8,814) |
| | **7-D) HPV vaccine delivery** | *7-h) HPV vaccine delivery | [7]; [16]; [19]; [20]; [24]; [25]; [29]; [31]; [32]; [33]; [36]; [37]. | n/a | 411 (327) | n/a (n/a) | [7]. | [7]. | 162 (162) | 162 (162) |

## References


[1] Stephens DP, Thomas TL. Cultural values influencing immigrant Haitian mothers' attitudes toward human papillomavirus vaccination for daughters. *Journal of Black Psychology* 2013; **39**(2): 156–68.

[2] McFadden SAM, Ko LK, Shankar M, et al. Development and evaluation of an online continuing education course to increase healthcare provider self-efficacy to make strong HPV vaccine recommendations to East African immigrant families. *Tumour Virus Res* 2021; **11**: 200214. DOI:10.1016/j.tvr.2021.200214.

[3] Lai D, Bodson J, Davis FA, et al. Diverse families' experiences with HPV vaccine information sources: a community-based participatory approach. *J Community Health* 2017; **42**: 400–12.

[4] Joseph NP, Clark JA, Bauchner H, et al. Knowledge, attitudes, and beliefs regarding HPV vaccination: ethnic and cultural differences between African-American and Haitian immigrant women. *Women's Health Issues* 2012; **22**(6): e571-9. DOI:10.1016/j.whi.2012.09.003.

[5] Wilson LA, Quan AML, Bota AB, et al. Newcomer knowledge, attitudes, and beliefs about human papillomavirus (HPV) vaccination. *BMC Fam Pract* 2021; **22**(1): 17. DOI:10.1186/s12875-020-01360-1.

[6] Perkins RB, Pierre-Joseph N, Marquez C, Iloka S, Clark JA. Parents' opinions of mandatory human papillomavirus vaccination: Does ethnicity matter? *Women's Health Issues* 2010; **20**: 420–6.

[7] Ayash C, Raad N, Finik J, et al. Arab American mothers' HPV vaccination knowledge and beliefs. *J Community Health* 2022; **47**: 716–25.

[8] Adegboyega A, Obielodan O, Wiggins AT, Dignan M, Williams LB. Beliefs and knowledge related to human papillomavirus (HPV) vaccine among African Americans and African immigrants young adults. *Cancer Causes and Control* 2023; **34**: 479–89.

[9] Khan A, Abonyi S, Neudorf C, Galea S, Ahmed S. Barriers to and facilitators of human papillomavirus vaccination in an ethnically diverse group of migrant parents: a qualitative mixed methods study. *Hum Vaccin Immunother* 2023; **19**(3): 2269721. doi: 10.1080/21645515.2023.2269721.

[10] Kim SW, Chen AC, Ou L, Larkey L, Todd M, Han Y. Developing a culturally and linguistically congruent digital storytelling intervention in Vietnamese and Korean American mothers of human papillomavirus-vaccinated children: feasibility and acceptability study. *JMIR Form Res* 2023; **7**: e45696. doi: 10.2196/45696.

[11] Mupandawana ET, Cross R. Attitudes towards human papillomavirus vaccination among African parents in a city in the north of England: a qualitative study. *Reprod Health* 2016; **13**(1): 97. DOI:10.1186/s12978-016-0209-x.

[12] Ramírez M, Jessop AB, Leader A, Crespo CJ. Acceptability of the human papillomavirus vaccine among diverse hispanic mothers and grandmothers. *Hispanic Health Care International* 2014; **12**: 24–33.

[13] Walter D, Atzpodien K, Pins C, Wichmann O, Reiter S. Einflussfaktoren auf die Inanspruchnahme von Impfungen durch Jugendliche mit Migrationshintergrund Eine qualitative Untersuchung mit Jugendlichen, Müttern und Ärzten [Factors influencing the uptake of vaccines by adolescents with migration background. A qualitative study of adolescents, mothers, and physicians]. *Bundesgesundheitsblatt Gesundheitsforschung Gesundheitsschutz* 2013; **56**: 1368–75.

[14] Zeraiq L, Nielsen D, Sodemann M. Attitudes towards human papillomavirus vaccination among Arab ethnic minority in Denmark: a qualitative study. *Scand J Public Health* 2015; **43**: 408–14.

[15] Dailey PM, Krieger JL. Communication and US-Somali Immigrant human papillomavirus (HPV) vaccine decision-making. *Journal of Cancer Education* 2017; **32**: 516–21.

[16] Celentano I, Winer RL, Jang SH, et al. Development of a theory-based HPV vaccine promotion comic book for East African adolescents in the US. *BMC Public Health* 2021; **21**(1): 1137. DOI:10.1186/s12889-021-11005-2.

[17] Scarinci IC, Garcés-Palacio IC, Partridge EE. An examination of acceptability of HPV vaccination among African American women and Latina immigrants. *J Womens Health* 2007; **16**: 1224–33.

[18] Gao H, Okoror TA, Hyner GC. Focus group study of Chinese international students' knowledge and beliefs about HPV vaccination, before and after reading an informational pamphlet about Gardasil®. *J Immigr Minor Health* 2016; **18**: 1085–92.

[19] Luque JS, Raychowdhury S, Weaver M. Health care provider challenges for reaching Hispanic immigrants with HPV vaccination in rural Georgia. *Rural Remote Health* 2012; **12**(2): 1975.

[20] Rubens-Augustson T, Wilson LA, Murphy MSQ, et al. Healthcare provider perspectives on the uptake of the human papillomavirus vaccine among newcomers to Canada: a qualitative study. *Hum Vaccin Immunother* 2019; **15**: 1697–707.

[21] Aragones A, Genoff M, Gonzalez C, Shuk E, Gany F. HPV vaccine and Latino immigrant parents: if they offer it, we will get it. *J Immigr Minor Health* 2016; **18**: 1060–5.

[22] Kim M, Lee H, Kiang P, Kim D. Human papillomavirus: a qualitative study of Korean American female college students' attitudes toward vaccination. *Clin J Oncol Nurs* 2017; **21**: E239–47.

[23] McComb E, Ramsden V, Olatunbosun O, Williams-Roberts H. Knowledge, attitudes and barriers to human papillomavirus (HPV) Vaccine uptake among an immigrant and refugee catch-up group in a Western Canadian province. *J Immigr Minor Health* 2018; **20**: 1424–8.

[24] Lindsay AC, Delgado D, Valdez MJ, Granberry P. Latinx fathers report low awareness and knowledge of the human papillomavirus vaccine, but high willingness to vaccinate their children if recommended by a healthcare provider: a qualitative study. *Glob Public Health* 2022; **17**: 2883–97.

[25] Vamos CA, Vázquez-Otero C, Kline N, et al. Multi-level determinants to HPV vaccination among Hispanic farmworker families in Florida. *Ethn Health* 2021; **26**: 319–36.

[26] Jackson C, Bedford H, Cheater FM, et al. Needles, jabs and jags: a qualitative exploration of barriers and facilitators to child and adult immunisation uptake among Gypsies, Travellers and Roma. *BMC Public Health* 2017; **17**(1): 254. DOI:10.1186/s12889-017-4178-y.

[27] Kobetz E, Menard J, Hazan G, et al. Perceptions of HPV and cervical cancer among haitian immigrant women: implications for vaccine acceptability. *Educ Health* 2011; **24**(3): 479.

[28] Burke NJ, Do HH, Talbot J, Sos C, Ros S, Taylor VM. Protecting our Khmer daughters: ghosts of the past, uncertain futures, and the human papillomavirus vaccine. *Ethn Health* 2015; **20**: 376–90.

[29] Javanbakht M, Stahlman S, Walker S, et al. Provider perceptions of barriers and facilitators of HPV vaccination in a high-risk community. *Vaccine* 2012; **30**: 4511–6.

[30] Perkins RB, Clark JA. Providers' perceptions of parental concerns about HPV vaccination. *J Health Care Poor Underserved* 2013; **24**(2): 828-39. doi: 10.1353/hpu.2013.0080.

[31] Gorman DR, Bielecki K, Willocks LJ, Pollock KG. A qualitative study of vaccination behaviour amongst female Polish migrants in Edinburgh, Scotland. *Vaccine* 2019; **37**: 2741–7.

[32] Vamos CA, Kline N, Vázquez-Otero C, et al. Stakeholders' perspectives on system-level barriers to and facilitators of HPV vaccination among Hispanic migrant farmworkers. *Ethn Health* 2022; **27**: 1442–64.

[33] Ganczak M, Bielecki K, Drozd-Dąbrowska M, et al. Vaccination concerns, beliefs and practices among Ukrainian migrants in Poland: a qualitative study. *BMC Public Health* 2021; **21**(1): 93. DOI:10.1186/s12889-020-10105-9.

[34] Pratt R, Njau SW, Ndagire C, et al. "We are Muslims and these diseases don't happen to us": a qualitative study of the views of young Somali men and women concerning HPV immunization. *Vaccine* 2019; **37**: 2043–50.

[35] Ko LK, Taylor VM, Mohamed FB, et al. "We brought our culture here with us": a qualitative study of perceptions of HPV vaccine and vaccine uptake among East African immigrant mothers. *Papillomavirus Research* 2019; **7**: 21–5.

[36] Ganczak M, Kalinowski P, Pasek O, et al. Health system barriers to child mandatory and optional vaccination among Ukrainian migrants in Poland in the context of MMR and HPV vaccines—a qualitative study. *Int J Environ Res Public Health* 2023; **20**(1): 712. DOI:10.3390/ijerph20010712.

[37] Garcia S, Hopfer S, Amaro H, Tanjasiri S. HPV vaccine delay and refusal among unvaccinated Mexican American young adult women: a qualitative investigation of Mexican-born and US-born HPV vaccine decision narratives. *J Behav Med* 2023; **46**: 88–99.

[38] Netfa F, King C, Davies C, et al. Perceived facilitators and barriers to the uptake of the human papillomavirus (HPV) vaccine among adolescents of Arabic-speaking mothers in NSW, Australia: a qualitative study. *Vaccine X* 2023; **14**: 100335. doi: 10.1016/j.jvacx.2023.100335.

[39] Chadenier GMC, Colzani E, Faccini M, Borriello CR, Bonazzi C. Assessment of the first HPV vaccination campaign in two northern Italian health districts. *Vaccine* 2011; **29**: 4405–8.

[40] Lu PJ, Rodriguez-Lainz A, O'Halloran A, Greby S, Williams WW. Adult vaccination disparities among foreign-born populations in the U.S., 2012. *Am J Prev Med* 2014; **47**: 722–33.

[41] Furgurson KF, Sandberg JC, Hsu FC, Mora DC, Quandt SA, Arcury TA. HPV knowledge and vaccine initiation among Mexican-born farmworkers in North Carolina. *Health Promot Pract* 2019; **20**: 445–54.

[42] Gerend MA, Zapata C, Reyes E. Predictors of human papillomavirus vaccination among daughters of low-income Latina mothers: the role of acculturation. *Journal of Adolescent Health* 2013; **53**: 623–9.

[43] Kepka D, Ding Q, Bodson J, Warner EL, Mooney K. Latino parents' awareness and receipt of the hpv vaccine for sons and daughters in a State with low three-dose completion. *J Cancer Educ* 2015; **30**(4): 808-12. doi: 10.1007/s13187-014-0781-0.

[44] Kepka DL, Ulrich AK, Coronado GD. Low knowledge of the three-dose HPV vaccine series among mothers of rural Hispanic adolescents. *J Health Care Poor Underserved* 2012; **23**(2): 626-35. doi: 10.1353/hpu.2012.0040.

[45] Marlow LA, Wardle J, Forster AS, Waller JJ. Ethnic differences in human papillomavirus awareness and vaccine acceptability. *Epidemiol Community Health* 2009; **63**(12): 1010-5. doi: 10.1136/jech.2008.085886.

[46] Remschmidt C, Fesenfeld M, Kaufmann AM, Deleré Y. Sexual behavior and factors associated with young age at first intercourse and HPV vaccine uptake among young women in Germany: implications for HPV vaccination policies. *BMC Public Health* 2014; **14**: 1248. doi: 10.1186/1471-2458-14-1248.

[47] Rondy M, van Lier A, van de Kassteele J, Rust L, de Melker H. Determinants for HPV vaccine uptake in the Netherlands: a multilevel study. *Vaccine* 2010; **28**(9): 2070-5. doi: 10.1016/j.vaccine.2009.12.042.

[48] van Boetzelaer E, Daae A, Winje BA, Vestrheim DF, Steens A, Stefanoff P. Sociodemographic determinants of catch-up HPV vaccination completion between 2016-2019 in Norway. *Hum Vaccin Immunother* 2022; **18**(1): 1976035. DOI:10.1080/21645515.2021.1976035

[49] Lindsay AC, Greaney ML, Rabello LM, Kim YY, Wallington SF. Brazilian immigrant parents' awareness of HPV and the HPV vaccine and interest in participating in future HPV-related cancer prevention study: an exploratory cross-sectional study conducted in the USA. *J Racial Ethn Health Disparities* 2020; **7**(5): 829-837. doi: 10.1007/s40615-020-00704-y.

[50] Lindsay AC, Pineda JA, Valdez MJ, Idalí Torres M, Granberry PJ. Central American immigrant parents' awareness, acceptability, and willingness to vaccinate their adolescent children against human papillomavirus: a pilot cross-sectional study. *Int J Environ Res Public Health* 2020; **17**(8): 2869. DOI:10.3390/ijerph17082869.

[51] Bhatta MP, Johnson DC, Lama M, Maharjan B, Lhaki P, Shrestha S. Cervical cancer and human papillomavirus vaccine awareness among married Bhutanese refugee and Nepali women in Eastern Nepal. *J Community Health* 2020; **45**: 516–25.

[52] Adjei Boakye E, Lew D, Muthukrishnan M, et al. Correlates of human papillomavirus (HPV) vaccination initiation and completion among 18-26 year olds in the United States. *Hum Vaccin Immunother* 2018; **14**: 2016–24.

[53] Adjei Boakye E, Zeng W, Governor S, et al. Differences in human papillomavirus (HPV) vaccine uptake by nativity status among men aged 18–34 years. *Prev Med Rep* 2019; **16**: 101010. DOI:10.1016/j.pmedr.2019.101010.

[54] Anuforo B, McGee-Avila JK, Toler L, et al. Disparities in HPV vaccine knowledge and adolescent HPV vaccine uptake by parental nativity among diverse multiethnic parents in New Jersey. *BMC Public Health* 2022; **22**(1): 195. doi: 10.1186/s12889-022-12573-7.

[55] Kepka D, Bodson J, Lai D, et al. Diverse caregivers' HPV vaccine-related awareness and knowledge. *Ethn Health* 2021; **26**: 811–26.

[56] Yi JK, Anderson KO, Le YC, Escobar-Chaves SL, Reyes-Gibby CC. English proficiency, knowledge, and receipt of HPV vaccine in Vietnamese-American Women. *J Community Health* 2013; **38**: 805–11.

[57] Ashing KT, Carrington A, Ragin C, Roach V. Examining HPV- and HPV vaccine-related cognitions and acceptability among US-born and immigrant hispanics and US-born and immigrant non-Hispanic Blacks: a preliminary catchment area study. *Cancer Causes and Control* 2017; **28**: 1341–7.

[58] Glenn BA, Tsui J, Singhal R, et al. Factors associated with HPV awareness among mothers of low-income ethnic minority adolescent girls in Los Angeles. *Vaccine* 2015; **33**(2): 289-93. doi: 10.1016/j.vaccine.2014.11.032.

[59] Kepka D, Bodson J, Lai D, et al. Factors associated with human papillomavirus vaccination among diverse adolescents in a region with low human papillomavirus vaccination rates. *Health Equity* 2018; **2**: 223–32.

[60] Colón-López V, Quiñones V, Del Toro-Mejías LM, et al. HPV awareness and vaccine willingness among Dominican immigrant parents attending a federal qualified health clinic in Puerto Rico. *J Immigr Minor Health* 2015; **17**: 1086–90.

[61] Lee HY, Choi YJ, Yoon YJ, Oh J. The role of English proficiency in Korean American immigrant women. *Clin J Oncol Nurs* 2018; **22**(3): E64-E70. doi: 10.1188/18.CJON.E64-E70.

[62] Cofie LE, Hirth JM, Guo F, Berenson AB, Markides K, Wong R. HPV vaccination among foreign-born women: examining the National Health Interview Survey 2013–2015. *Am J Prev Med* 2018; **54**: 20–7.

[63] Cofie LE, Tailor HD, Lee MH, Xu L. HPV vaccination uptake among foreign-born Blacks in the US: insights from the National Health Interview Survey 2013–2017. *Cancer Causes and Control* 2022; **33**: 583–91.

[64] Hernandez ND, Daley EM, Young L, et al. HPV vaccine recommendations: does a health care provider's gender and ethnicity matter to unvaccinated Latina college women? *Ethn Health* 2019; **24**: 645–61.

[65] De P, Budhwani H. [65] #240 - De 2017 Human papillomavirus (HPV) vaccine initiation in minority Americans. *Public Health* 2017; **144**: 86–91.

[66] Escobar B, Amboree TL, Sonawane K, et al. Human papillomavirus awareness among foreign- and US-born Hispanics, United States, 2017-2018. *Prev Med Rep* 2021; **22**: 101379. doi: 10.1016/j.pmedr.2021.101379.

[67] Barnack-Tavlaris JL, Garcini LM, Macera CA, Brodine S, Klonoff EA. Human papillomavirus vaccination awareness and acceptability among U.S.-born and U.S. foreign-born women living in California. *Health Care Women Int* 2016; **37**(4): 444-62. doi: 10.1080/07399332.2014.954702.

[68] Budhwani H, De P. Human papillomavirus vaccine initiation in Asian Indians and Asian subpopulations: a case for examining disaggregated data in public health research. *Public Health* 2017; **153**: 111–7.

[69] Chu H, Ko LK, Ibrahim A, et al. The impact of an educational forum intervention on East African mothers' HPV vaccine-related knowledge, attitudes, and intentions to vaccinate their adolescent children. *Vaccine* 2021; **39**: 3767–76.

[70] Mueller NT, Noone AM, Luta G, Wallington SF, Huerta EE, Mandelblatt JS. Information channels associated with awareness of human papillomavirus infections and vaccination among Latino immigrants from safety net clinics. *J Immigr Minor Health* 2012; **14**: 183–8.

[71] Agénor M, Abboud S, Delgadillo JG, Pérez AE, Peitzmeier SM, Borrero S. Intersectional nativity and racial/ethnic disparities in human papillomavirus vaccination initiation among U.S. women: a national population-based study. *Cancer Causes Control* 2018; **29**(10): 927-936. doi: 10.1007/s10552-018-1069-1.

[72] Napolitano F, Gualdieri L, Santagati G, Angelillo IF. Knowledge and attitudes toward HPV infection and vaccination among immigrants and refugees in Italy. *Vaccine* 2018; **36**: 7536–41.

[73] Khodadadi AB, Hansen B, Kim Y il, Scarinci IC. Latinx immigrant mothers' perceived self-efficacy and intentions regarding human papillomavirus vaccination of their daughters. *Women's Health Issues* 2022; **32**: 293–300.

[74] Becerra MB, Avina RM, Mshigeni S, Becerra BJ. Low human papillomavirus literacy among Asian-American women in California: an analysis of the California Health Interview Survey. *J Racial Ethn Health Disparities* 2020; **7**(4): 678-686. doi: 10.1007/s40615-020-00698-7.

[75] Wemrell M, Vicente RP, Merlo J. Mapping sociodemographic and geographical differences in human papillomavirus non-vaccination among young girls in Sweden. *Scand J Public Health* 2023; **51**: 288–95.

[76] Wiessner C, von dem Knesebeck O, Gerlich MG, Briken P, Becher H. Migration and sexual health services use – results from the German Health and Sexuality Survey (GeSiD). *Sexuality Research and Social Policy* 2022; **19**: 1383–1394. https://doi.org/10.1007/s13178-022-00691-1.

[77] Bodson J, Warner EL, Kepka D. Moderate awareness and limited knowledge relating to cervical cancer, HPV, and the HPV vaccine among Hispanics/Latinos in Utah. *Health Promot Pract* 2016; **17**: 548–56.

[78] Bhattacharya M, Xiong S, McRee AL. Nativity differences in awareness and knowledge about HPV infection and vaccination among U.S. adults: findings from a national population-based sample. *J Immigr Minor Health* 2022; **24**: 794–8.

[79] Pérez AE, Agénor M, Gamarel KE, Operario D. Nativity disparities in human papillomavirus vaccination among U.S. adults. *Am J Prev Med* 2018; **54**(2): 248-258. doi: 10.1016/j.amepre.2017.10.019.

[80] Lee YM, Riesche L, Lee H, Shim K. Parental HPV knowledge and perceptions of HPV vaccines among Korean American parents. *Applied Nursing Research* 2018; **44**: 54–9.

[81] Fowler B, Bodson J, Warner EL, Dyer J, Kepka D. Poor HPV vaccine-related awareness and knowledge among Utah Latinas overdue for recommended cancer screenings. *J Community Health* 2016; **41**: 825–37.

[82] Tsui J, Singhal R, Rodriguez HP, Gee GC, Glenn BA, Bastani R. Proximity to safety-net clinics and HPV vaccine uptake among low-income, ethnic minority girls. *Vaccine* 2013; **31**(16): 2028-34. doi: 10.1016/j.vaccine.2013.02.046.

[83] McElfish PA, Narcisse MR, Felix HC, et al. Race, nativity, and sex disparities in human papillomavirus vaccination among young adults in the USA. *J Racial Ethn Health Disparities* 2021; **8**(5): 1260-1266. doi: 10.1007/s40615-020-00886-5.

[84] Gelman A, Miller E, Schwarz EB, Akers AY, Jeong K, Borrero S. Racial disparities in human papillomavirus vaccination: does access matter?. *J Adolesc Health* 2013; **53**(6): 756-62. doi: 10.1016/j.jadohealth.2013.07.002

[85] Minhat HS, Mustafa J, Sarabi S. Sociodemographic determinants of knowledge on HPV vaccination among Iranian women living in Malaysia. *Academic Journal of Cancer Research* 2013; **6**(1): 8-12.

[86] Bastani R, Glenn BA, Tsui J, et al. Understanding suboptimal human papillomavirus vaccine uptake among ethnic minority girls. *Cancer Epidemiology Biomarkers and Prevention* 2011; **20**: 1463–72.

[87] Sundaram MK, Almutary AG, Haque S, SM F, Hussain A. Awareness of human papilloma virus and its association with cervical cancer among female university students: a study from United Arab Emirates. *Oncologie* 2021; **23**: 269–77.

[88] Khodadadi AB, Redden DT, Scarinci IC. HPV vaccination hesitancy among Latina immigrant mothers despite physician recommendation. *Ethn Dis* 2020; **30**: 661–70.

[89] Guo Y, Sims OT, Li Q, Yang F. Factors associated with first-generation immigrant parents' unwillingness to vaccinate their daughters for HPV. *J Migr Health* 2023; **7**: 100161. DOI:10.1016/j.jmh.2023.100161.

[90] Ratnasamy P, Chagpar AB. HPV vaccination and factors influencing vaccine uptake among people of Indian ancestry living in the United States. *Epidemiol Infect* 2022; **150:** e152. DOI:10.1017/S0950268822001315.

[91] Groene EA, Horvath KJ, Yared N, et al. Missed opportunities for human papillomavirus vaccination by parental nativity, Minnesota, 2015-2018. *Public Health Rep* 2022; **137**(5): 867-877. doi: 10.1177/00333549211027244.

[92] Alsulami FT, Sanchez J, Rabionet SE, Popovici I, Baraka MA. Predictor of HPV vaccination uptake among foreign-born college students in the U.S.: an exploration of the role of acculturation and the Health Belief Model. *Vaccines* 2023; **11**(2): 422. DOI:10.3390/vaccines11020422.

[93] Berman RS, Smock L, Bair-Merritt MH, Cochran J, Geltman PL. Giving it our best shot? human papillomavirus and hepatitis B virus immunization among refugees, Massachusetts, 2011-2013. *Prev Chronic Dis* 2017; **14**: E50. doi: 10.5888/pcd14.160442.

[94] Chen AC, Kim SW, Ou L, Todd M, Larkey L. Digital storytelling intervention to promote human papillomavirus vaccination among at-risk Asian immigrant populations: pilot intervention study. *JMIR Form Res* 2023; **7**: e46951. doi: 10.2196/46951.

[95] Han SH, Kang HS, Eum K, Seo JY, Karsten K. Factors influencing human papillomavirus vaccination among Asian immigrant college students during the COVID-19 pandemic. *SAGE Open 2023*; **13**(4). DOI:10.1177/21582440231207451

[96] Lee HY, Won CR, Luo Y, Lee MH. Human papillomavirus (HPV) and HPV vaccine awareness among Korean American immigrants in Alabama: would internet use improve health awareness? *Asian Pac J Cancer Prev* 2023; **24**(8): 2575-2581. doi: 10.31557/APJCP.2023.24.8.2575.

[97] Lee JY, Lee H, Kim S, Park S, Chae D, Hong HC. HPV vaccination stages and associated factors among children of immigrant women. *Public Health Nurs* 2024. doi: 10.1111/phn.13431.

[98] Liu CH, Ling J, Liu C, Schrader K, Ammigan R, Mclntire E. Vaccination rates among international students: insights from a university health vaccination initiative. *J Am Coll Health* 2024; **72**(9): 3322-3329. doi: 10.1080/07448481.2022.2155470.

[99] Mullassery D, Posmontier B. The effects of health beliefs and acculturation on the acceptance of human papillomavirus vaccination among Asian Indian parents in the United States. *J Am Assoc Nurse Pract* 2024; **36**(11): 637-646. doi: 10.1097/JXX.0000000000001068.

[100] Karki I, Dobbs PD, Larson D, Maness SB. Human papillomavirus (HPV) knowledge, beliefs, and vaccine uptake among United States and international college students. *J Am Coll Health* 2022; **70**(8): 2483-2490. doi: 10.1080/07448481.2020.1865982.

[101] Amdisen L, Kristensen ML, Rytter D, Mølbak K, Valentiner-Branth P. Identification of determinants associated with uptake of the first dose of the human papillomavirus vaccine in Denmark. *Vaccine* 2018; **36**: 5747–53.

[102] Hansen BT, Campbell S, Burger E, Nygård M. Correlates of HPV vaccine uptake in school-based routine vaccination of preadolescent girls in Norway: a register-based study of 90,000 girls and their parents. *Prev Med (Baltim)* 2015; **77**: 4–10.

[103] Tung ILY, Machalek DA, Garland SM. Attitudes, knowledge and factors associated with Human Papillomavirus (HPV) vaccine uptake in adolescent girls and young women in Victoria, Australia. *PLoS One* 2016; **11**(8): e0161846. DOI:10.1371/journal.pone.0161846.

[104] Wang J, Ploner A, Sparén P, et al. Mode of HPV vaccination delivery and equity in vaccine uptake: A nationwide cohort study. *Prev Med (Baltim)* 2019; **120**: 26–33.

[105] Fernández de Casadevante V, Cantarero-Arévalo L, Cuesta JG, Valentiner-Branth P. Ethnic background and human papillomavirus vaccine uptake in Denmark: a countrywide retrospective cohort study including 274,154 women aged 19-28 years. *Papillomavirus Research* 2016; **2**: 78–84.

[106] Møller SP, Kristiansen M, Norredam M. Human papillomavirus immunization uptake among girls with a refugee background compared with Danish-born girls: a national register-based cohort study. *European Journal of Cancer Prevention* 2018; **27**: 42–5.

[107] Hertzum-Larsen R, Thomsen LT, Frederiksen K, Kjær SK. Human papillomavirus vaccination in immigrants and descendants of immigrants in Denmark. *European Journal of Cancer Prevention* 2020; **29**: 149–56.

[108] Dong L, Nygård M, Hansen BT. Sociodemographic correlates of human papillomavirus vaccine uptake: opportunistic and catch-up vaccination in Norway. *Cancers (Basel)* 2021; **13**(14): 3483. DOI:10.3390/cancers13143483.

[109] Suppli CH, Dreier JW, Rasmussen M, et al. Sociodemographic predictors are associated with compliance to a vaccination-reminder in 9692 girls age 14, Denmark 2014–2015. *Prev Med Rep* 2018; **10**: 93–9.

[110] Slåttelid Schreiber SM, Juul KE, Dehlendorff C, Kjær SK. Socioeconomic predictors of human papillomavirus vaccination among girls in the Danish childhood immunization program. *J Adolesc Health* 2015; **56**(4): 402-7. doi: 10.1016/j.jadohealth.2014.12.008.

[111] Bollerup S, Baldur-Felskov B, Dehlendorff C, Kjaer SK. Socioeconomic predictors of human papillomavirus vaccination in Danish men – A nationwide study. *Papillomavirus Research* 2017; **3**: 18–23.

[112] Bjerke RD, Laake I, Feiring B, Aamodt G, Trogstad L. Time trends in HPV vaccination according to country background: a nationwide register-based study among girls in Norway. *BMC Public Health* 2021; **21**(1): 854. DOI:10.1186/s12889-021-10877-8.

[113] Algren MH, Gazibara T, Valentiner-Branth P, Timmermann A, Thygesen LC, Tolstrup JS. Characteristics associated with non-initiation and non-completion of human papillomavirus vaccination among Danish girls: a nationwide register-based cohort study. *Scand J Public Health* 2024: 14034948241232462. doi: 10.1177/14034948241232462.

[114] Charania NA, Kirkpatrick L, Paynter J, Turner N. Childhood vaccination uptake among children born in Aotearoa New Zealand based on parental nationality. *Hum Vaccin Immunother* 2023; **19**(2): 2240688. doi: 10.1080/21645515.2023.2240688.

[115] Du C, Voaklander D, Meherali S, Paudel YR, MacDonald SE. Human papillomavirus vaccine coverage among immigrant adolescents in Alberta: a population-based cohort study. *Int Health* 2024: ihae038. doi: 10.1093/inthealth/ihae038.

[116] Voss SS, Nørgaard SK, Valentiner-Branth P. Identification of subgroups in the Danish population for targeted human papillomavirus vaccination efforts. *Vaccine* 2023; **41**(23): 3525-3533. doi: 10.1016/j.vaccine.2023.04.060.

[117] Scarinci IC, Hansen B, Kim YI. HPV vaccine uptake among daughters of Latinx immigrant mothers: Findings from a cluster randomized controlled trial of a community-based, culturally relevant intervention. *Vaccine* 2020; **38**(25): 4125-4134. doi: 10.1016/j.vaccine.2020.03.052.
